# Monoamine Reuptake Inhibitors in Parkinson's Disease

**DOI:** 10.1155/2015/609428

**Published:** 2015-02-25

**Authors:** Philippe Huot, Susan H. Fox, Jonathan M. Brotchie

**Affiliations:** ^1^Toronto Western Research Institute, Toronto Western Hospital, University Health Network, 399 Bathurst Street, Toronto, ON, Canada M5T 2S8; ^2^Division of Neurology, Movement Disorder Clinic, Toronto Western Hospital, University Health Network, University of Toronto, 399 Bathurst Street, Toronto, ON, Canada M5T 2S8; ^3^Department of Pharmacology and Division of Neurology, Faculty of Medicine, Université de Montréal and Centre Hospitalier de l'Université de Montréal, Montréal, QC, Canada

## Abstract

The motor manifestations of Parkinson's disease (PD) are secondary to a dopamine deficiency in the striatum. However, the degenerative process in PD is not limited to the dopaminergic system and also affects serotonergic and noradrenergic neurons. Because they can increase monoamine levels throughout the brain, monoamine reuptake inhibitors (MAUIs) represent potential therapeutic agents in PD. However, they are seldom used in clinical practice other than as antidepressants and wake-promoting agents. This review article summarises all of the available literature on use of 50 MAUIs in PD. The compounds are divided according to their relative potency for each of the monoamine transporters. Despite wide discrepancy in the methodology of the studies reviewed, the following conclusions can be drawn: (1) selective serotonin transporter (SERT), selective noradrenaline transporter (NET), and dual SERT/NET inhibitors are effective against PD depression; (2) selective dopamine transporter (DAT) and dual DAT/NET inhibitors exert an anti-Parkinsonian effect when administered as monotherapy but do not enhance the anti-Parkinsonian actions of L-3,4-dihydroxyphenylalanine (L-DOPA); (3) dual DAT/SERT inhibitors might enhance the anti-Parkinsonian actions of L-DOPA without worsening dyskinesia; (4) triple DAT/NET/SERT inhibitors might exert an anti-Parkinsonian action as monotherapy and might enhance the anti-Parkinsonian effects of L-DOPA, though at the expense of worsening dyskinesia.

## 1. Introduction

The cardinal manifestations of Parkinson's disease (PD) are secondary to a degeneration of dopaminergic neurons of the substantia nigra (SN), which causes a deficiency of dopamine in the striatum [1–9]. In addition to this striatal dopamine deficiency, there is also loss of dopamine in the cerebral cortex [10]. The serotonergic [4, 10–14] and noradrenergic [4, 10, 15] systems also undergo degeneration in PD, leading to decreased levels of serotonin (5-hydroxytryptamine, 5-HT) and noradrenaline in both striatal and extrastriatal structures.

Thus, in PD, degenerative changes extend beyond the dopaminergic system and the interactions described between the dopaminergic, serotonergic, and noradrenergic systems are perturbed. Currently, dopamine replacement therapy with L-3,4-dihydroxyphenylalanine in combination with an aromatic L-amino acid decarboxylase (AADC) inhibitor such as benserazide or carbidopa (henceforth referred to as L-DOPA) is the mainstay of PD treatment [16, 17]. However, L-DOPA targets mainly the dopamine-related pathology of PD and fails to address the decreases in both 5-HT and noradrenaline. In addition, with increasing duration of L-DOPA therapy, a range of motor and nonmotor complications, encompassing dyskinesia, wearing-off, and psychiatric manifestations, develop [18, 19].

Because they can increase the levels of monoamine in the synaptic cleft by inhibiting the action of the monoamine transporters, monoamine reuptake inhibitors (MAUIs) represent potential agents in the therapy of PD. As will be discussed in this review article, their uses extend beyond the motor symptoms of the disease. Several of these compounds, with different affinities and pharmacological profiles, have been tested in animal models of PD and idiopathic PD. Such assessments have been made against different manifestations of the disease, sometimes with contradictory results. In interpreting the findings described we feel that some benefits of MAUIs might be mitigated by the fact that the great majority of these compounds display affinity not only for the monoamine transporters, but also for a myriad of neurotransmitter receptors. Indeed, this makes interpretation of individual datasets difficult but, in summary, we feel the actions related directly to specific transporters become clearer. In reviewing data, we also note that many of the studies published are case-reports or nonrandomised, unblinded, uncontrolled trials. In many cases we believe that the “ideal” pharmacological profile against a particular symptom of the disease has not been discovered yet or that the clinical use of the currently available drugs is not optimal based on their pharmacological profile. Clearly, a better understanding of the effects of MAUIs in PD based on their selectivity profile will lead to development of better anti-Parkinsonian drugs and to an improvement of patient care; this is one goal of this review.

This review article summarises the studies involving MAUIs that were performed in idiopathic PD and animal models of PD. The aim of this review is to provide an overview of the effects of MAUIs against different symptoms of PD and to establish what the optimal monoamine reuptake profile might be in order to target specific manifestations of the disease, either as monotherapy or as an adjunct to L-DOPA therapy.

## 2. Methods

**Figure 1:**
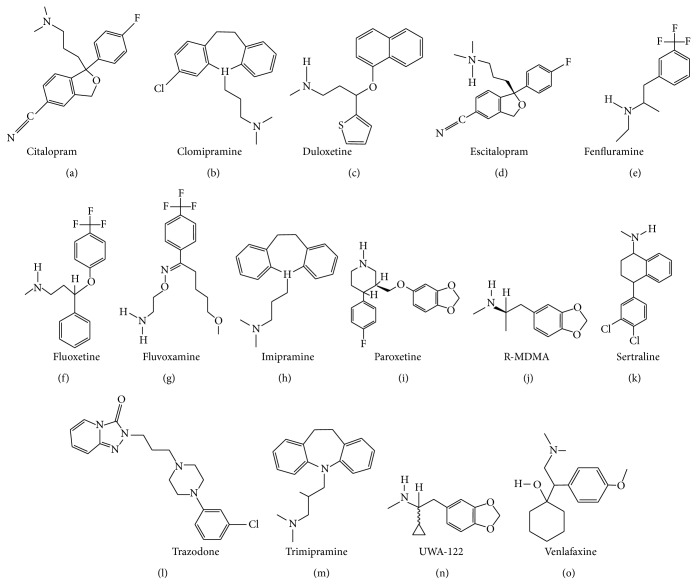
The chemical formulae of the selective SERT inhibitors that were studied in PD. Compounds with such a pharmacological profile are citalopram and its S-enantiomer escitalopram, clomipramine, duloxetine, fenfluramine, fluoxetine, fluvoxamine, imipramine, paroxetine, R-MDMA, sertraline, trazodone, trimipramine, UWA-122, and venlafaxine. Chemical formulae (except for UWA-122) were adapted from PubChem (http://pubchem.ncbi.nlm.nih.gov/).

**Figure 2:**
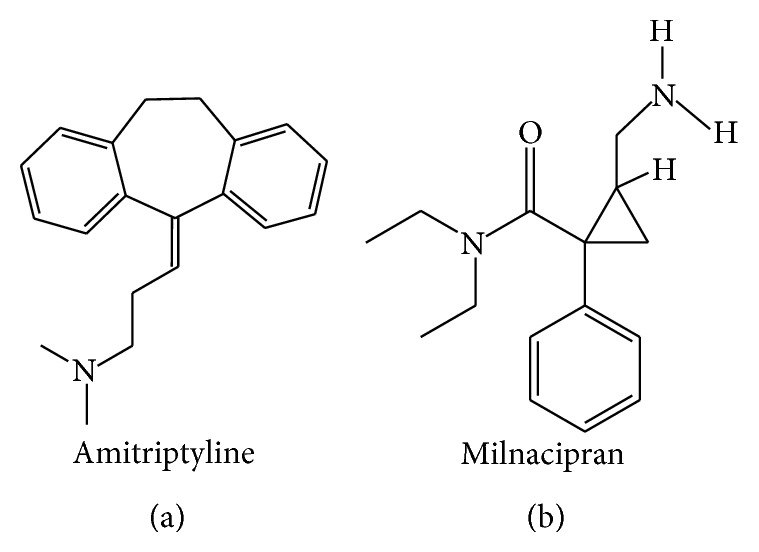
The chemical formulae of the dual SERT = NET inhibitors that were studied in PD. Compounds with such a pharmacological profile are amitriptyline and milnacipran. Chemical formulae were adapted from PubChem (http://pubchem.ncbi.nlm.nih.gov/).

**Figure 3:**
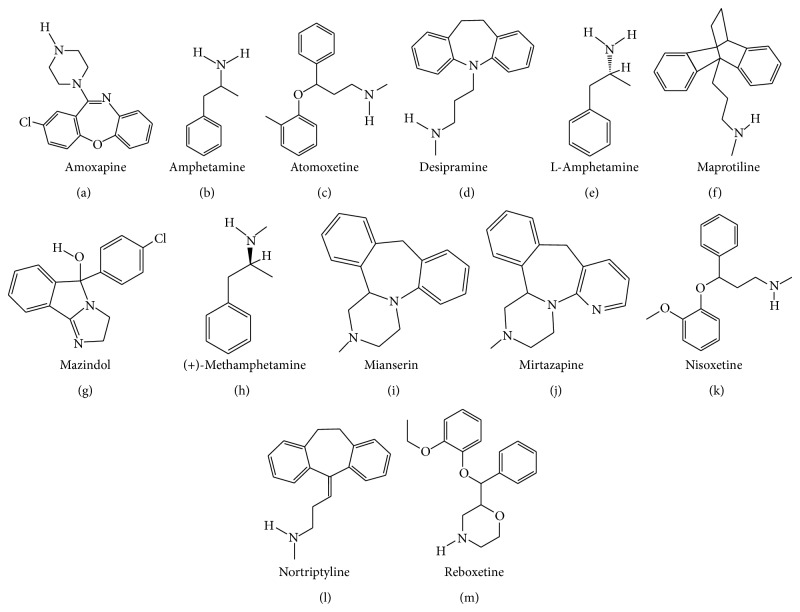
The chemical formulae of the selective NET inhibitors that were studied in PD. Compounds with such a pharmacological profile are amoxapine, amphetamine, atomoxetine, desipramine, L-amphetamine, maprotiline, mazindol, mianserin, mirtazapine, nisoxetine, nortriptyline, and reboxetine. Although (+)-methamphetamine was not studied in PD, its chemical formula is included because the racemate methamphetamine was studied in the disease. Chemical formulae were adapted from PubChem (http://pubchem.ncbi.nlm.nih.gov/).

**Figure 4:**
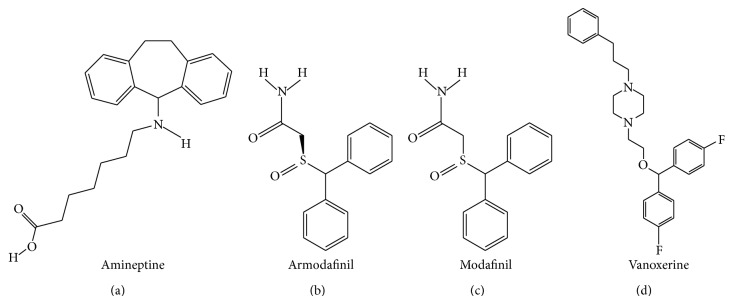
The chemical formulae of the selective DAT inhibitors that were studied in PD. Compounds with such a pharmacological profile are amineptine, modafinil, SEP-228,791, and vanoxerine. The chemical formula of SEP-228,791 has not been disclosed yet and is thus not included in the figure. Chemical formulae were adapted from PubChem (http://pubchem.ncbi.nlm.nih.gov/).

**Figure 5:**
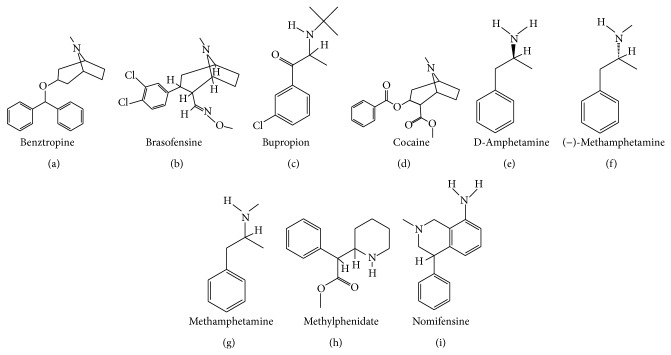
The chemical formulae of the dual DAT = NET inhibitors that were studied in PD. Compounds with such a pharmacological profile are benztropine, brasofensine, bupropion, cocaine, D-amphetamine, methamphetamine, methylphenidate, and nomifensine. Although (−)-methamphetamine was not studied in PD, its chemical formula is included because the racemate methamphetamine was studied in the disease. Chemical formulae were adapted from PubChem (http://pubchem.ncbi.nlm.nih.gov/).

**Figure 6:**
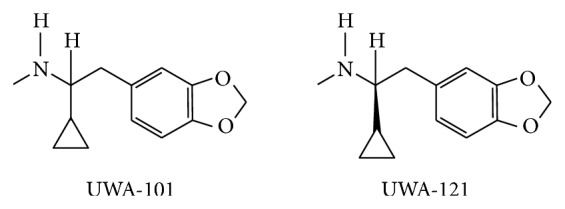
The chemical formulae of dual DAT = SERT inhibitors that were studied in PD. Compounds with such a profile are UWA-101 and its R-enantiomer UWA-121.

**Figure 7:**
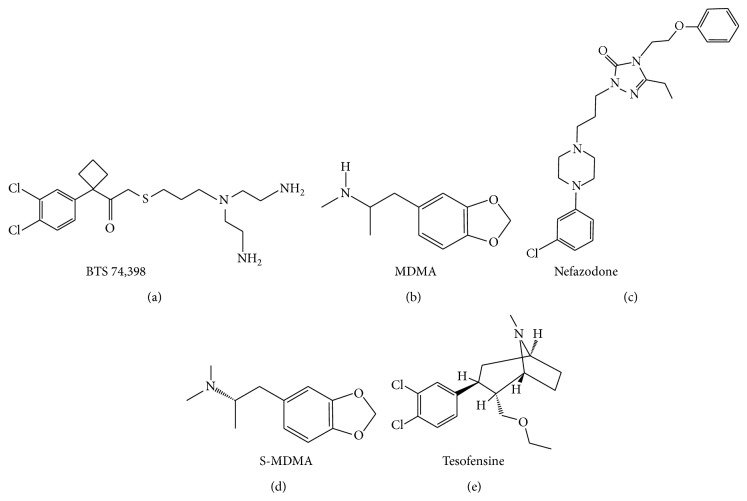
The chemical formulae of non-selective DAT = NET = SERT inhibitors that were studied in PD. Compounds with such a profile are BTS 74,398, MDMA, nefazodone, S-MDMA, and tesofensine. Chemical formulae were adapted from PubChem (http://pubchem.ncbi.nlm.nih.gov/).

**Figure 8:**
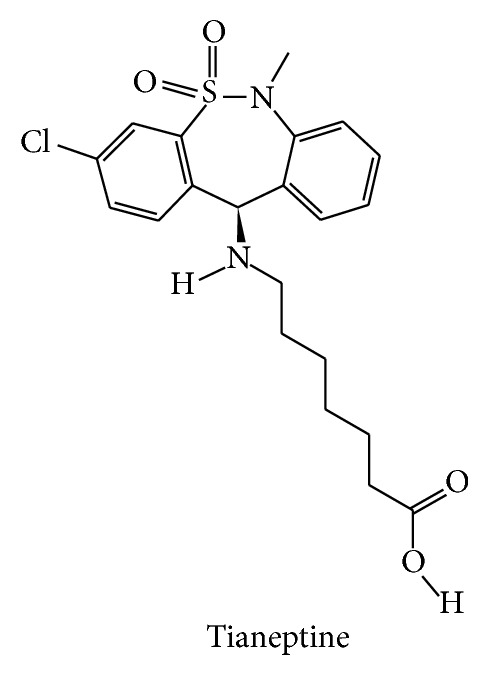
The chemical formula of tianeptine, the only SERT enhancer that was studied in PD. Chemical formula was adapted from PubChem (http://pubchem.ncbi.nlm.nih.gov/).

Literature was searched through PubMed (http://www.ncbi.nlm.nih.gov/PubMed/) and cross-referencing. Extended search was performed using Google (http://www.google.ca). Updates on the ongoing clinical trials were found on the National Institute of Health (http://clinicaltrials.gov/), Parkinson Pipeline Project (http://www.pdpipeline.org/), PD trials (http://www.pdtrials.org/, last accessed 2nd Feb. 2015), PD Online Research (http://www.pdonlineresearch.org/), and Michael J. Fox Foundation (http://www.michaeljfox.org/) websites. Chemical formulae of the compounds (Figures 1–8) were adapted from PubChem (http://pubchem.ncbi.nlm.nih.gov/). Some patents were also included in the search and were retrieved from the United States Patent and Trademark Office (http://patft.uspto.gov/). In addition, abstracts from the American Academy of Neurology (AAN), American Neurological Association, Movement Disorders Society (MDS), Society for Neuroscience, and World Parkinson Congress from the 2007–2014 meetings (included) were reviewed. The key words used for the search are shown as follows: 1-methyl-4-phenyl-1,2,3,6-tetrahydropyridine, 3,4-methylenedioxymethamphetamine, 5-HT, 5-HT syndrome, 5-HT transporter, 5-hydroxytryptamine, 6-hydroxydopamine, 6-OHDA, 6-OHDA-rat, affinity, akinesia, amineptine, amitriptyline, amoxapine, amphetamine, antidepressant, armodafinil, atomoxetine, benztropine, binding, bradykinesia, brasofensine, BTS 74,398, bupropion, citalopram, clomipramine, cocaine, common marmoset, cynomolgus macaque, D-amphetamine, DAT, depression, desipramine, desvenlafaxine, dextroamphetamine, dimepramine, dopamine, dopamine transporter, duloxetine, dyskinesia, EC_50_, Ecstasy, escitalopram, fenfluramine, fluoxetine, fluvoxamine, GBR-12,909, IC_50_, imipramine, Kd, Ki, L-amphetamine, levoamphetamine, macaque, maprotiline, marmoset, mazindol, MDMA, methamphetamine, methylphenidate, mianserin, milnacipran, mirtazapine, modafinil, monkey, monoamine transporter, monoamine uptake, monoamine uptake inhibitors, motor complications, motor fluctuations, mouse, MPTP, MPTP mouse, MPTP-macaque, MPTP-marmoset, MPTP-squirrel monkey, nefazodone, NET, neurotoxicity, nisoxetine, nomifensine, non-motor, noradrenaline, noradrenaline transporter, norepinephrine, norepinephrine transporter, nortriptyline, NS 2214, NS 2330, off-time, on-time, Org 3770, Parkinson, Parkinson disease, Parkinson's disease, Parkinsonian, Parkinsonism, paroxetine, patent, PET, pharmacological, pharmacology, post mortem, potency, propylhexedrine, psychosis, reboxetine, rhesus macaque, rigidity, R-MDMA, R,R-hydroxybupropion, selective serotonin reuptake inhibitor, SEP-226,330, SEP-228,791, serotonin, serotonin syndrome, serotonin transporter, SERT, sertraline, S-MDMA, SPECT, squirrel monkey, S,S-hydroxybupropion, SSRI, TCA, tesofensine, tetracyclic antidepressant, tianeptine, toxicity, trazodone, tremor, trimipramine, tricyclic antidepressant, UPDRS, vanoxerine, venlafaxine, visual hallucinations, and wearing-off.

The affinity of the MAUIs discussed in this review for the three monoamine transporters is presented in Table 1. As can be seen in Table 1, there is discrepancy in the literature as to the relative potency of the compounds for the different monoamine transporters, depending on the methodology used and the way the results are presented in the different studies, for example, half-maximal effective concentration (EC_50_) versus dissociation constant (Kd). Whereas both the EC_50_ and the Kd (in other instances, the half-maximal inhibitory concentration [IC_50_] and the inhibitory constant [Ki]) indicate the interaction of a compound with a target, the EC_50_ and the IC_50_ are indicators of the compound's biological activity, while the Kd and Ki represent its affinity [20–23]. Although a broad range of affinities at the monoamine transporters is displayed for the majority of compounds, only the smallest value of the range was considered in order to determine their relative potency. A compound was considered selective for a monoamine transporter if its potency at that site was five times greater than at another site. In addition, throughout the paper, a compound is considered to exhibit high affinity for a site if its Kd for this site is <1,000 nM. Moderate affinity is attributed for Kd between 1,000 and 10,000 nM and weak affinity for Kd > 10,000 nM.  Table 2 presents a list of all of the compounds included in this review based on their primary monoamine transporter affinity.

The main focus of the current review article is, however, about clinical and observational human studies that were published, since these are more numerous than rodent and nonhuman primate studies. The animal models included in this review are the 1-methyl-4-phenyl-1,2,3,6-tetrahydropyridine- (MPTP-) lesioned mouse and primate, as well as the 6-hydroxydopamine- (6-OHDA-) lesioned rat. Except for a few studies, the haloperidol-induced catalepsy or reserpine-treated rat models are not discussed or comprehensively reviewed, because the former is a postsynaptic parkinsonism, whereas the latter is a transient parkinsonism based upon monoamine depletion, two conditions that differ from idiopathic PD, and where the effects of presynaptically targeted MAUIs are also likely to differ from PD. Similarly, the cases of drug-induced Parkinsonism—except for cases relating to a worsening of a preexisting PD following the onset of drug therapy—are not reviewed here. For that reason, many of the molecules discussed here have been used as radioligands, in either* postmortem* studies or* in vivo* imaging studies; some studies are cited in the current paper, but not all of them, as this review article is centered on pharmacological studies. Another aim of the present review was to detail sufficiently the literature cited so that the reader could understand the experimental design and the main outcomes of the studies without having to read the articles. However, that was not always possible, as details were sometimes missing, especially in the literature from the pre-L-DOPA era. Other reasons accounting for an occasional lack of detail include when studies have been presented exclusively as abstracts or have been published in non-English or non-French journals.

Selegiline (L-deprenyl) is an inhibitor of the monoamine oxidase (MAO) type B (MAO-B) that has been used in the treatment of Parkinson's disease for decades [24–26]. Selegiline is metabolised into L-amphetamine and L-methamphetamine [27–30]. However, as the primary use of selegiline in PD is as a MAO-B inhibitor, studies performed with selegiline in PD patients are not reviewed in the current paper. Similar issues apply to amantadine, which is widely used in Parkinson's disease, mostly for its glutamate antagonist and anticholinergic properties [31], but which also exhibits affinity for both NET and DAT [32].

Lastly, some compounds discussed here, encompassing amphetamine, its enantiomers, and derivatives such as fenfluramine, act both as monoamine reuptake inhibitors and as monoamine release enhancers (references with individual compounds). These drugs thus have two mechanisms of action which result in elevated extrasynaptic monoamine levels: (1) inhibitory capacity relating to their competition with the endogenous amine for reuptake and (2) release of monoamines via reversal of the transporter. Notwithstanding these two mechanisms, these drugs will be considered as monoamine reuptake inhibitors here.

## 3. Monoamine Transporters

### 3.1. Dopamine Transporter and Parkinson's Disease

The dopamine transporter (DAT) is found exclusively on dopaminergic neurons [33–35]. Its gene (SLC6A3) was first cloned in 1991 and is located on chromosome 5 [36–39]. The human DAT gene is a 64-kilobase (kb) gene that contains 15 exons and 14 introns [40] and that codes for a 620-amino acid protein [41]. Both N- and C-terminals are located intracellularly [35]. Homo-oligomerisation of DAT monomers is important for DAT expression and function [42]. DAT interacts with the PDZ (postsynaptic density protein [PSD_95_]) domain-containing protein PICK_1_ [43]. DAT also interacts with other intracellular proteins such as Hic_5_, protein kinase C (PKC), protein phosphatase 2A (PP_2A_), Rack_1_, synuclein, and syntaxin; these proteins regulate DAT expression, membrane distribution, and activity [35]. Two Na^+^ and one Cl^−^ ions are cotransported with each dopamine^+^ ion [44]. The orphan nuclear receptor* Nurr1* enhances DAT expression [45].

DAT is required for MPTP and 6-OHDA to be neurotoxic to dopaminergic neurons, as both toxins enter the neurons via DAT [46–48]. DAT levels in the striatum of PD patients are markedly reduced [12, 49–51].

### 3.2. Serotonin Transporter and Parkinson's Disease

The serotonin transporter (SERT) gene (SLC6A4) was cloned in 1991 [52, 53]. It is localised on chromosome 17, spans about 24 kb, and contains 13 exons. Like DAT, SERT is Na^+^ and Cl^−^ dependent [54]. Human brain and platelet SERT is a 630-amino acid protein [55]. Like DAT, SERT interacts with the PDZ domain-containing protein PICK_1_, but the interaction is weaker than DAT's [43]. SERT monomers also form oligomers [56].

In PD, both* postmortem* and positron emission tomography (PET) studies have demonstrated a reduction in SERT levels in the cortex and basal ganglia [12, 57].

### 3.3. Noradrenaline Transporter and Parkinson's Disease

The noradrenaline transporter (NET) gene (SLC6A2) was cloned in 1991 [58]. The gene is located on chromosome 16, spans 45 kb, contains 14 exons, and codes for a 617-amino acid protein [41]. Like DAT and SERT, NET is Na^+^ and Cl^−^ dependent [59]. Three splice variants have been described, but only two appear to be functional [60]. Like DAT and SERT, it interacts with the PDZ domain-containing protein PICK_1_ [43]. NET also interacts with *α*-synuclein [61].

As mentioned above, there is a degeneration of the noradrenergic system in PD. However, to our knowledge, only one studied assessed the fate of NET in the brain of PD patients. This PET study used [^11^C]-RTI-32—a nonspecific DAT/NET ligand—as their radioligand and did not include a control group. It showed that [^11^C]-RTI-32 binding levels were decreased in locus coeruleus (LC) and anterior cingulate gyrus when depressed PD patients were compared to nondepressed PD patients [62]. As DAT is expressed only in dopaminergic neurons [35], the changes encountered in the LC are likely to reflect degeneration of noradrenergic neurons.

### 3.4. Interactions between the Monoamine Systems and Relevance to Parkinson's Disease

The dopaminergic, serotonergic, and noradrenergic systems are intimately connected with each other. Thus,* in vivo* stimulation of the dorsal raphe nucleus (DRN) in the rat leads to elevations of 5-hydroxyindoleacetic acid (5-HIAA, a metabolite of 5-HT) and dihydroxyphenylacetic acid (DOPAC, a metabolite of dopamine) in the striatum [63] and, in the rat, unilateral electrolytical lesions of the median raphe nucleus lead to increased 5-HIAA and DOPAC levels in the striatum [64]. Dopamine can directly activate serotonergic type 2A (5-HT_2A_) [65], type 1A (5-HT_1A_), type 2C (5-HT_2C_), and type 3 (5-HT_3_) receptors [66]. In the MPTP-lesioned mouse model of PD, there is a 5-HT hyperinnervation of the striatum, potentially a response to compensate for the loss of afferent dopaminergic fibres [67]. Similar findings were encountered in the striatum of the 6-OHDA-lesioned rat model of PD, in both adult-lesioned [68–70] and neonatal-lesioned rats [71, 72].

It was further demonstrated that, in the 6-OHDA-lesioned rat, exogenous L-DOPA is metabolised into dopamine by striatal serotonergic terminals [73–77]. It is believed that this dopamine derived from 5-HT terminals acts as a false neurotransmitter and plays an important role in the emergence of L-DOPA-induced dyskinesia [78–80]. In addition to L-DOPA, dopamine itself can be uptaken by SERT [81, 82]. In cerebral areas in which DAT levels are low, such as the frontal cortex, NET can reuptake dopamine [83–86]. A recent study suggested that L-DOPA-derived dopamine could be uptaken by NET in the striatum of 6-OHDA-lesioned rats because of an increase in dopamine levels following desipramine administration [87]. However, as displayed in Table 1, desipramine exhibits high affinity at SERT and is thus not selective for NET, raising doubt on the authors' conclusions. Lastly, noradrenaline can be uptaken by and released from 5-HT terminals [88].

Thus, by restoring physiological levels of monoamines in the brain, MAUIs could theoretically restore physiological interactions between the monoamines and their transporters, leading to an alleviation of both motor and nonmotor symptoms of PD, as well as to a decrease in treatment-related complications.

The remainder of this review will consider each of the three main classes of MAUIs, that is, SERT, DAT, and NET inhibitors, and will describe their effect on motor and nonmotor symptoms of PD and treatment-related complications, either as monotherapy or as adjuncts to L-DOPA.

## 4. SERT Inhibitors

### 4.1. Overview

SERT inhibitors are widely used as antidepressants. Antidepressants are the most extensively studied MAUIs in PD. Selective serotonin reuptake inhibitors (SSRIs) and tricyclic antidepressants (TCAs) are the most prescribed antidepressants in PD [89–92]. Despite the wide use of antidepressants in PD, concerns remain about their efficacy for depression, safety, and tolerability. In 2003, a Cochrane systematic review concluded that there were insufficient data on the effectiveness against depression and safety of antidepressants in PD to make recommendations [93] and, in 2005, a review and meta-analysis of antidepressants in PD were unable to demonstrate a difference in efficacy between placebo and active treatment [94]. A recent meta-analysis also found a lack of superiority when SSRIs were compared to placebo for PD depression, but stated that SSRIs were generally well tolerated in PD [95]. However, a study in which depressed PD patients were treated with paroxetine, nortriptyline, or placebo found that specific parameters such as somatic anxiety and lack of interest were significantly improved by active treatment [96]. Moreover, a recent study provided class I evidence that the SSRI paroxetine was effective in treating depression in PD (see below) [97, 98]. Taken together, these data suggest that SSRIs are probably effective against depression and may also effectively alleviate other aspects of PD nonmotor symptomatology, such as anxiety and apathy. One case-series [99] and one case-report [100] suggested that the SERT inhibitors citalopram and venlafaxine might also alleviate dopaminergic psychotic features.

SSRIs have been linked to potentially life-threatening adverse effects when combined to MAO inhibitors [101]. One of these potentially life-threatening adverse events is the 5-HT syndrome, which fortunately occurs fairly rarely when antidepressants are combined with MAO-B inhibitors such as selegiline or rasagiline [102–115]. At present, there is not enough evidence to recommend avoiding the combination of rasagiline and SSRIs, although patients should be informed of the potential interaction between the molecules and of the symptoms of the 5-HT syndrome. It is noteworthy that as many as 7% of PD patients may be taking an antidepressant [116] and that, considering this relatively high figure, the 5-HT syndrome has only seldom been reported. The STACCATO study, which reviewed charts to document the occurrence of 5-HT syndrome in PD patients treated with rasagiline and antidepressants, should provide further data on this important topic [117].

The possibility of worsening preexisting Parkinsonism by adding an antidepressant remains controversial, mainly for SSRIs. Thus, a vast pharmacoepidemiologic study found no significant increase in risk of motor deterioration upon antidepressant therapy in PD [118]. In addition, a patient-level meta-analysis encompassing 2064 PD patients from the FS1 and FS-TOO (Futility Study I and Futility Study II), ELLDOPA (Earlier versus Later Levodopa Therapy in Parkinson's Disease), QE2 (Effects of CoEnzyme Q10 in Early Parkinson's Disease), TEMPO (TVP-1012 in Early Monotherapy for Parkinson's Disease Outpatients), and PRECEPT (Parkinson Research Examination of CEP1348 Trial), in which 451 subjects were taking antidepressants, showed that TCAs delay the time to dopaminergic therapy, suggesting that they might exert some disease-modifying effect. That meta-analysis also demonstrated that antidepressants have no effect on the annual Unified Parkinson's Disease Rating Scale (UPDRS) part III decline rate [119–123]. Accordingly, the SSRIs sertraline, paroxetine, and fluoxetine were demonstrated to improve haloperidol-induced catalepsy in mice, suggesting that they may not worsen Parkinsonism in human [124].

However, a retrospective study found a faster increase in anti-Parkinsonian medication amongst SSRI-treated than TCA- or placebo-treated depressed PD patients [125]. A mechanism by which SERT inhibitors could worsen Parkinsonism may involve 5-HT-mediated activation of 5-HT_2C_ receptors within the SN, which leads to a decrease in nigrostriatal dopaminergic transmission [126]. Alternately, SERT inhibitors might worsen Parkinsonism via a 5-HT-mediated activation of presynaptic 5-HT_1A_ receptors [127], which would result in less dopamine being released from raphe-striatal terminals. If the last mechanism was demonstrated to play a role in the pathophysiology of L-DOPA-induced dyskinesia [78], its involvement as a determinant of the severity of Parkinsonism or of the efficacy of L-DOPA anti-Parkinsonian action remains hypothetical.

### 4.2. Citalopram and Escitalopram

In addition to their high affinity for SERT, citalopram and its L-enantiomer escitalopram show moderate/weak affinity for both DAT and NET (Table 1). They also exhibit moderate affinity for the alpha (*α*) 1 adrenoceptors (Kd of 1.2 and 3.9 *μ*M, resp.), muscarinic (M) type 1 receptors (Kd of 1.4 and 1.2 *μ*M, resp.), and 5-HT_2C_ receptors (Kd of 2.1 and 2.5 *μ*M, resp.). In addition, citalopram has high affinity for the histamine (H) type 1 receptors (Kd of 0.28 *μ*M), whereas escitalopram has moderate affinity for H_1_ receptors (Kd of 2.0 *μ*M) [128]. Citalopram also has high affinity for the sigma (*σ*) type 1 and moderate affinity for the *σ*_2_ receptors (Kd of 0.29 and 5.4 *μ*M, resp.) [129]. The interaction with the *σ* receptors might be relevant for the treatment of PD, since the *σ*_1_ receptor antagonist and 5-HT_1A_ receptor agonist BMY-14802 was demonstrated to reduce abnormal involuntary movements (AIMs) in the L-DOPA-treated 6-OHDA-lesioned rat [130, 131]. The chemical formulae of citalopram and escitalopram are illustrated in Figure 1.

#### 4.2.1. Citalopram

In the MPTP-lesioned mouse, citalopram (1, 5, and 10 mg/kg intraperitoneally (i.p.)) significantly reduced the duration of rapid-eye-movement (REM) sleep. However, the magnitude of REM-sleep reduction was similar in the saline-treated animals [132]; thus the effect of citalopram on REM-sleep may not be due to an interaction with the disease process.

In the 6-OHDA-lesioned rat, chronic daily treatment with citalopram (40 mg/kg i.p.) resulted in a significant reduction in L-DOPA-induced abnormal involuntary movements (AIMs) severity after 2 months of treatment [133]; that study did not assess the effect of citalopram on the anti-Parkinsonian efficacy of L-DOPA. In the 6-OHDA-lesioned, acute challenges of citalopram (2, 3, and 5 mg/kg i.p.) significantly reduced the severity of AIMs and rotational behaviour, without impairing the anti-Parkinsonian action of L-DOPA [134, 135]. In another study,* de novo* treatment with citalopram (3 and 5 mg/kg subcutaneously (s.c.)) reduced the development of L-DOPA-induced AIMs, in the 6-OHDA-lesioned rat [136, 137]. Collectively, these results suggest that both acute and chronic treatment with a SERT inhibitor might reduce dyskinesia severity, while* de novo* treatment with a SERT inhibitor might attenuate the priming leading to the expression of dyskinesia. However, a retrospective study conducted in 111 PD patients with a follow-up of at least 10 years found that administration of selective SERT inhibitors does not prevent dyskinesia development, thought it may delay their onset [138], somewhat contradicting the preclinical rodent data. On another note, in a study presented as an abstract, pretreatment of rats with citalopram and desipramine 30 min prior to 6-OHDA administration in the striatum prevented contralateral forepaw hyperalgesia [139].

In a case-report study, citalopram (20 mg orally (p.o.) once a day (id)) caused marked deterioration of the UPDRS part III subscore. Following the introduction of citalopram, UPDRS part III subscore was 53 in the off-state and 21 in the on-state, compared to 30 in the off-state and 16 in the on-state following discontinuation of citalopram. Tremor was the most severely affected item of the subscale [140]. In a case-report study, an 80-year-old man with PD and depression developed auditory hallucinations while on citalopram (10 and 20 mg p.o. id), which resolved upon discontinuation. No mention was made of the efficacy of citalopram on depressive or motor symptoms [141]. In another case-report, citalopram (10 mg p.o. id) effectively treated pathological crying in a 66-year-old PD patient [142]. Citalopram (20 mg p.o. id and then 40 mg p.o. id) reportedly unmasked PD in a 68-year-old woman with major depression, although no nuclear imaging was performed to determine whether there was striatal dopamine denervation [143].

In a prospective, 8-week open-label trial, 10 PD patients with depression were administered flexible doses of citalopram (average final dose of 19 mg p.o. id). Eight patients completed the trial. Citalopram significantly improved the Hamilton Depression Rating Scale (HDRS) score. Worsening of motor function was reported in one patient [144], although no formal evaluation using the UPDRS part III was reported.

In a randomised, double-blind, placebo-controlled trial performed in 37 PD patients, citalopram (10–20 mg p.o. id) failed to significantly improve the HDRS score, when compared to placebo [145]. In this 52-week study, there was an important dropout rate (79% in the placebo group and 66% in the citalopram group). Ten patients in the placebo group and 4 patients in the citalopram group reported an increase in Parkinsonism, although no formal assessment was performed.

In a randomised, double-blind, placebo-controlled trial, citalopram (20 mg p.o. id) and desipramine (a NET inhibitor with mild selectivity over SERT, see Table 1; up to 25 mg p.o. three times a day (tid)) were compared in 48 nondemented PD patients with depression [146, 147]. In this study, desipramine significantly improved the Montgomery Asberg Depression Rating Scale (MADRS) score 14 days after the beginning of therapy, when compared to citalopram and placebo. On day 30, both desipramine and citalopram had significantly improved the MADRS score when compared to placebo. The Hamilton Anxiety Rating Scale (HARS) was also significantly improved in both antidepressant groups on day 30. Although no formal motor assessment was made, the antidepressants did not worsen Parkinsonism when compared to placebo. This study suggests that, in PD, NET > SERT inhibitors exert their antidepressant effect quicker than selective SERT inhibitors.

One single-blind, semirandomised study specifically assessed the effect of citalopram on parkinsonian disability in 32 depressed and nondepressed PD patients [148]. In this study, citalopram (20 mg p.o. id) significantly improved items 23 and 31 of the UPDRS part III (finger tapping and body bradykinesia) when compared to baseline, at one and 4 months after the beginning of therapy. The total UPDRS part III subscore was also significantly improved in both depressed and nondepressed PD patients, as were the Beck Depression Inventory (BDI) and the HDRS scores in the depressed group.

In a 12-week open-label, unblinded, uncontrolled study, 12 PD patients with major depression were administered citalopram (10–30 mg p.o. id). Citalopram significantly improved the HDRS and the MADRS scores and was also beneficial against hypokinesia, rigidity, and dyskinesia but led to a worsening of tremor. The authors also measured regional cerebral blood flow (rCBF) using single-photon computed emission tomography (SPECT) and found that citalopram treatment led to a significant reduction of a previously increased rCBF in the left frontal dorsolateral region [149, 150]. Another 12-week, open-label, unblinded, uncontrolled study encompassing 11 PD patients with major depression was published by the same authors. Citalopram (10–30 mg p.o. id) was administered and significantly improved both the HDRS and MADRS scores. Citalopram did not lead to changes in levels of homovanillic acid (HVA), 5-HIAA, the noradrenaline metabolite 3-methoxy-4-hydroxyphenylglycol (MHPG, MOPEG), brain-derived neurotrophic factor (BDNF), orexin-A, interleukin-6, or corticosterone [151].

In a randomised, double-blind, placebo-controlled, cross-over trial conducted in 21 PD patients without psychiatric comorbidity, an acute challenge of citalopram (40 mg p.o.) was administered. Citalopram had a negative effect on visual verbal learning task, exacerbated subscores of depression, anger, and anxiety, and had no effect on concept shifting task, UPDRS part III, or reaction time [152].

In a 6-month open-label, single-blind, uncontrolled study of 52 depressed PD patients, citalopram (*N* = 13; 20 mg p.o. id), fluvoxamine (*N* = 13; 150 mg p.o. id), fluoxetine (*N* = 13; 20 mg p.o. id), and sertraline (*N* = 13; 50 mg p.o. id) were evaluated. The BDI and HDRS scores were both improved by each of the four SSRIs, without difference between the drugs. None of these SSRIs worsened significantly the UPDRS part II and III subscores, but two patients with fluvoxamine and two patients with fluoxetine experienced an exacerbation of tremor [153].

In a randomised, double-blind, placebo-controlled, cross-over trial, citalopram (acute administration of 30 mg p.o.) significantly reduced the stop signal reaction time and enhanced inferior frontal activation, as assessed by multimodal magnetic resonance imaging [154].

An evidence-based medicine (EBM) review published by the MDS in 2011 stated that there was “insufficient evidence” regarding the efficacy of citalopram for the treatment of depression in PD to make any recommendation [155].

#### 4.2.2. Escitalopram

The efficacy of escitalopram was evaluated in an open-label, flexible dose study of 14 PD patients with depression. In this study, escitalopram (10–20 mg p.o. id) significantly improved the HDRS score and did not significantly change the UPDRS score [156]. In a Phase II prospective, randomised, double-blind, double-dummy, placebo-controlled trial, escitalopram (10–20 mg p.o. id) significantly improved depression without adverse motor effects, although there was a trend towards a worsening of Parkinsonism [157].

There is one case-report of confusion and hallucinations triggered by the addition of the MAO inhibitor rasagiline to escitalopram in a 66-year-old PD woman [158]. Escitalopram also triggered hallucinations, aggressive behaviour, and disinhibition when administered to a 66-year-old PD woman who was previously on L-DOPA and entacapone [159].

### 4.3. Clomipramine

In addition to its high affinity for SERT, clomipramine displays moderate affinity for both NET and DAT (Table 1) and high affinity for *α*_1_, 5-HT_2A_, dopamine (D) D_3_, 5-HT_2C_, D_2_, 5-HT_3_, and *α*_2A_ receptors (Kd of 15.5, 35.5, 50.1, 64.6, 77.6, 85.1, and 525 nM, resp.) [160]. Clomipramine also binds to serotonergic type 7 (5-HT_7_) receptors with a Kd of 127 nM [161]. The chemical formula of clomipramine is provided in Figure 1.

In a study published as an abstract conducted in the 6-OHDA-lesioned rat, clomipramine (7.5, 15, or 30 mg i.p.) alleviated L-DOPA-induced AIMs [162].

In a case-report study, clomipramine (100 mg intravenously (i.v.) id) was administered for seven days to a 60-year-old PD man afflicted by a depression with psychotic features. This i.v. regimen led to an improvement of the depression and the patient was started on clomipramine 150 mg p.o. id ten days later. This treatment was continued for 6 months without recurrence of either the depression or the psychotic manifestations. The effect of clomipramine on Parkinsonism was not mentioned [163]. In a nonrandomised, double-blind, placebo-controlled study of 20 depressed PD patients, clomipramine (50–150 mg p.o. id) significantly improved HDRS, without worsening Parkinsonism [164].

### 4.4. Duloxetine

Duloxetine is a selective SERT inhibitor that exhibits high affinity for both DAT and NET (Table 1). Duloxetine also binds to serotonergic type 6 (5-HT_6_), 5-HT_2A_, 5-HT_2C_, serotonergic type 1E (5-HT_1E_), serotonergic type 2B (5-HT_2B_), 5-HT_7_, serotonergic type 1B (5-HT_1B_), serotonergic type 1F (5-HT_1F_), *α*_2_, and D_2_ receptors (Kd of 0.42, 0.50, 0.92, 3.7, 2.1, 2.3, 4.0, 4.4, 8.6, 14 *μ*M, resp.). The affinity of duloxetine at serotonergic type 4 (5-HT_4_), type 1D (5-HT_1D_), and 5-HT_1A_ receptors is unclear (Kd > 1.0, >3.0, and >5.0 *μ*M, resp.). Duloxetine also binds to H_1_, M, and *α*_1_ receptors, as well as to the MAO-B and MAO-A (EC_50_ of 2.3, 3.0, 8.3, 18, and 87 *μ*M, resp.) [165]. The chemical formula of duloxetine is displayed in Figure 1.

In a case-report study, a 71-year-old man with Parkinsonism showed near absent striatal uptake in a DAT SPECT scan with [^123^I]-ioflupane while taking duloxetine (dose not mentioned). After duloxetine discontinuation, the uptake was increased on both sides but moderately decreased on the right side, consistent with the patient's symptoms. No mention was made of the effect of duloxetine on Parkinsonism. The man also took escitalopram for a while, but no mention of escitalopram on Parkinsonian disability was made [166].

In a 6-week open-label study, 23 PD patients with pain were administered duloxetine (60 mg p.o. id). 20 patients completed the study. Duloxetine had a significant effect on pain, as assessed by questionnaires, but 7 patients reported no improvement with the drug. Duloxetine had no effect on the BDI and UPDRS scores. Tremor was worsened in 3 patients [167].

In a single-center, open-label study presented as an abstract, duloxetine (30 mg p.o. id, increased to 60 mg p.o. id if well tolerated) was administered to 10 PD patients with depression for 12 weeks. Seven patients completed the study and saw an improvement of the HDRS score, with a reduction on anxiety and pain, without effect on motor function [168].

In an open-label, noncomparative, multi-centre study, duloxetine (60 mg p.o. id) was administered during 12 weeks to 151 PD patients with depression. Duloxetine significantly improved the BDI and the HDRS and had no effect on the UPDRS motor score, but nearly 10% of patients experienced adverse effects [169].

In a 12-week randomised, open-label, parallel-group study, the antidepressant efficacy of duloxetine (*N* = 30, 60 mg p.o. id) was compared to sertraline (*N* = 30, 50 mg p.o. id) in 60 depressed PD patients. More patients recovered in the duloxetine than in the sertraline group [170].

### 4.5. Fenfluramine

In addition to its affinity for SERT, fenfluramine has moderate affinity for NET (Table 1). Upon binding to the transporters, fenfluramine inhibits monoamine reuptake and enhance monoamine release [171]. Fenfluramine also binds to 5-HT_2C_, 5-HT_2B_, and 5-HT_2A_ receptors (Kd of 3.2, 4.1, and 5.2 *μ*M, resp.) [172]. The chemical formula of fenfluramine is illustrated in Figure 1.

Fenfluramine is an amphetamine derivative that was shown to be toxic to serotonergic neurons in rat [173]. When administered to the 6-OHDA-lesioned rat, fenfluramine (20 mg/kg i.p.) induced bidirectional rotations, with a nonsignificant predominance of rotations ipsilateral to the lesioned side. The total number of rotations was less than those induced by either apomorphine or methamphetamine (2.5 mg/kg i.p.) and did not change following striatal embryonic stem cell graft [174]. In the 6-OHDA-lesioned rat, fenfluramine (2.5 mg/kg i.p., but not 0.25 mg/kg i.p.) significantly reduced AIMs but did not change the rotation number, when administered 5 minutes prior to L-DOPA [175]. In the 6-OHDA-lesioned rat which received intrastriatal grafts of embryonic dopaminergic cells primed with L-DOPA to exhibit AIMs, fenfluramine (2 mg/kg i.p.) did not induce dyskinesia, whereas fenfluramine (5 and 10 mg/kg i.p.) suppressed motor behaviour [176]. In the bilateral 6-OHDA-lesioned rat, administration of fenfluramine (2 mg/kg) increased the number of head twitches, a rodent correlate of psychotic activity [177].

In a study, a single challenge of fenfluramine (60 mg p.o.) was administered to 11 PD patients with major depression, 22 nondepressed PD patients, and 20 age-/gender-matched controls. Following the challenge, there was an elevation of serum prolactin levels in healthy controls. Although a prolactin elevation was noted in PD patients, its magnitude was significantly lower than in the control group. Prolactin elevation in the depressed PD group was significantly smaller than in the nondepressed group. No difference in cortisol response could be detected between groups following fenfluramine intake. The clinical effects of fenfluramine administration were not reported [178]. In a similar study, a single challenge of fenfluramine (60 mg p.o. id) was administered to 10 men with PD. Administration of fenfluramine did not alter plasma levels of adrenocorticotropic hormone (ACTH) or cortisol [179].

In a randomised, double-blind, placebo-controlled study, fenfluramine (20 mg p.o. four times a day (qid)) was administered to 10 PD patients for two weeks. Fenfluramine produced no effect on Parkinsonism, whether patients were treated with L-DOPA or not [180].

### 4.6. Fluoxetine

Fluoxetine is a SERT inhibitor that displays moderate/weak affinity for both DAT and NET (Table 1). Fluoxetine also exhibits moderate/weak affinity (EC_50_ > 1.6 *μ*M) for the *α*_1_, *α*_2_, and beta (*β*) adrenoceptors, H_1_ and H_2_ receptors, M, opioid, serotonin, dopamine, and *σ*_2_ receptors [129, 181, 182]. Fluoxetine has higher affinity for the *σ*_1_ receptor with a Kd of 0.24 *μ*M [129]. The chemical formula of fluoxetine is presented in Figure 1.

In the vesicular monoaminergic transporter (VMAT) type 2-deficient mouse model of PD [183], administration of fluoxetine decreased the immobility time during the forced swim test (Porsolt test) and exerted an antidepressant effect on the tail suspension test [184, 185]. Although the Porsolt test is traditionally seen as a measure of behavioural despair [186], its interpretation in the context of motor deficits is difficult, as the animals have to swim. Thus, in the case of a Parkinsonian animal, an improvement of the parkinsonian condition following drug administration, fluoxetine in the present instance, would also ameliorate the test performance and would not be necessarily indicative of an effect on behavioural despair.

In the 6-OHDA-lesioned rat, fluoxetine administration (15 and 20 mg/kg i.v.) alters the firing pattern of neurons of the LC, but not of neurons of the DRN. Fluoxetine normally inhibits LC neuronal firing, but this inhibition is weaker following 6-OHDA lesion. Under normal circumstances, fluoxetine inhibits serotonergic DRN firing; this effect of fluoxetine on DRN neurons is unchanged following 6-OHDA lesion [187].

In the 6-OHDA-lesioned rat, fluoxetine (5 and 10 mg/kg i.p.) administered as monotherapy for 10 days exacerbates Parkinsonian disability, whereas fluoxetine (1 mg/kg i.p.) administered over 10 days improved Parkinsonism, assessed by the bar-test [188].

In the 6-OHDA-lesioned rat, fluoxetine (10 mg/kg i.p.) induces rotations ipsilateral to the lesioned side. This rotational behaviour is maintained after striatal embryonic stem cell grafting [174]. In the 6-OHDA-lesioned rat, acute challenges of fluoxetine (5, 10, and 20 mg/kg i.p.) significantly reduced the severity of AIMs and rotational behaviour, though the anti-Parkinsonian action of L-DOPA was impaired with the 10 and 20 mg/kg doses [134].

Importantly, fluoxetine increases cellular proliferation in the subgranular zone (SGZ) when administered to parkinsonian rats. Thus, in PD patients and Parkinsonian rodents, dopamine depletion decreases precursor cell proliferation in both the subependymal layer and the SGZ [189]. When administered as monotherapy to 6-OHDA-lesioned rats for 14 days, fluoxetine (5 mg/kg i.p. id), but not the NET inhibitor maprotiline (10 mg/kg i.p. id), significantly reversed the reduction in SGZ cellular proliferation [190]. Fluoxetine (18 mg/kg p.o. daily for 33 days) also reversed the decreased hippocampal neurogenesis in the A53T-synuclein transgenic mouse, possibly via an increase in levels of BDNF and GDNF [191, 192]. The relevance of these promising findings for the treatment of PD remains to be determined, but it would be interesting to determine if fluoxetine therapy has an effect on the neurogenesis occurring in the striatum of adult primates [193, 194]. In addition to this interesting neuroproliferative effect, fluoxetine could protect against MPTP toxicity in the mouse, by reducing microglial activation and the expression of proinflammatory cytokines [195]. A study performed in primary rat midbrain cultures showed similar results, whereby fluoxetine attenuated neurodegeneration induced by lipopolysaccharide or 1-methyl-4-phenyl-pyridinium (MPP+) [196].

As mentioned previously and as can be inferred from the studies presented so far, the effect of SERT inhibitors on Parkinsonism is unclear, though some reports suggest they may worsen Parkinsonism. The mechanism by which fluoxetine and possibly all other SSRIs might worsen parkinsonian features might be related to a 5-HT_1A_-mediated reduction of striatal dopamine release. Thus, in the 6-OHDA-lesioned rat, fluoxetine reduced striatal dopamine levels following L-DOPA administration by 41%; 5-HT_1A_ receptors are likely to participate in the phenomenon, since the 5-HT_1A_ antagonist WAY-100,635 reversed the decrease [127]. Unfortunately, the behavioural correlates of these changes in striatal dopamine levels were not provided. Thus, worsening of the parkinsonian phenotype in PD patients treated with SSRIs may be related to a reduction of striatal dopamine levels and, because it does not occur in every SSRI-treated PD patient, a threshold phenomenon is possibly involved. If this hypothesis is correct, patients with more severe striatal dopamine depletion and probably more advanced/severe disease phenotype might be more susceptible to experiencing a worsening of their motor symptoms with SSRI therapy.

Two pharmacokinetic studies with fluoxetine were performed in PD patients [197, 198]. Because no clinical correlates were provided, these will not be discussed further.

In a case-report study, a 68-year-old man with PD experienced an improvement of anxiety and depression with fluoxetine (20 mg p.o. id) [199]. In a case-series of four PD patients, administration of fluoxetine (20 mg p.o. id) worsened Parkinsonism, evaluated by UPDRS part III subscore and parkinsonism improved following discontinuation of fluoxetine [200].

In a randomised study published in Chinese, administration of fluoxetine 20 mg id to 60 depressed PD patients significantly improved HDRS and increased levels of BDNF, two effects that were increased by simultaneous electroacupuncture therapy [201].

In a randomised, placebo-controlled study comparing repetitive transcranial magnetic stimulation with fluoxetine (20 mg p.o. id) in 21 PD patients with major depression, both treatments improved depression (both BDI and HDRS scores). No effects were noted on UPDRS part III subscore [202].

In a randomised, single-blind study, fluoxetine (20 mg p.o. id) improved depression in 21 PD patients (both HDRS and BDI) without significantly worsening UPDRS part III subscore, when compared to baseline [203]. In a prospective, open-label, uncontrolled study performed in 18 PD patients, fluoxetine improved HDRS score without affecting motor function. However, the dropout rate was 50% [204].

In another study, fluoxetine (20 mg p.o. id), sertraline (75–100 mg p.o. id), or paroxetine (20 mg p.o. id) was administered for 12 weeks to 12 patients with Parkinsonism, 5 of whom had idiopathic PD. When compared to baseline, antidepressant treatment significantly improved HDRS scores of the idiopathic and secondary PD groups. Both sertraline and paroxetine worsened the severity of Parkinsonism in one patient [205].

In a randomised, double-blind study, fluoxetine (20 mg p.o. id) effectively enhanced cognition in 12 depressed PD patients [206]. In a randomised, single-blind single-photon emission computed tomography (SPECT) study performed by the same group, fluoxetine (20 mg p.o. id) led to an increase in rCBF in the posterior cingulate gyrus and the occipital lobe and a decrease in rCBF in the right medial frontal gyrus, in 13 depressed PD patients [207].

An open-label, single-blind study of 7 L-DOPA-responsive PD patients evaluated the effect of fluoxetine on apomorphine-induced dyskinesia following a 12-hour withdrawal of anti-Parkinsonian medication. Fluoxetine (20 mg p.o. twice a day (bid) started 11 days prior to assessment) significantly reduced the severity of apomorphine-induced dyskinesia when compared to baseline, without deleterious effect on apomorphine anti-Parkinsonian action [208].

A few studies specifically addressed the issue of fluoxetine treatment and severity of Parkinsonism and, unfortunately, did not provide consistent results. In two case-report studies, fluoxetine increased the severity of Parkinsonism [209, 210]. In a case-series of 5 PD patients treated with L-DOPA and either cabergoline or placebo, fluoxetine (10–20 mg p.o. id) worsened UPDRS part III subscore in two patients [211]. In a 1-month add-on study of 14 PD patients with depression, fluoxetine (20 mg p.o. id) did not significantly modify UPDRS part III subscore but significantly improved tremor severity and MADRS score [212]. In a retrospective study that reviewed the medical records of 23 PD patients treated with fluoxetine (up to 40 mg p.o. id), Parkinsonism was adversely affected in three patients, reportedly improved in two patients and remained unchanged in 18 patients [213]. In a case-series of 23 PD patients, fluoxetine (10–40 mg p.o. id) did not worsen Parkinsonism, except for one patient who was also on selegiline [214]. In a randomised, double-blind, placebo-controlled study of 43 depressed patients with Parkinsonism (28 with idiopathic PD), fluoxetine (20 mg p.o. id for 8 weeks) led to a reduction of bradykinesia and rigidity, but exacerbated tremor. Depression was also improved, as evaluated by the HDRS, BDI, and MADRS scales [215]. In a prospective open-label controlled study, fluoxetine (20 mg p.o. id) significantly improved depression in 18 PD patients, without worsening Parkinsonism over 80 days. Fluoxetine and its metabolite norfluoxetine reached steady-state plasma levels after 18 days of administration [216].

All of the aforementioned studies were performed in nondemented depressed PD patients and the effect of adding fluoxetine in the subpopulation of demented PD patients remains unknown. In a case-report, however, adding fluoxetine (20 mg p.o. id) to L-DOPA and bromocriptine in a 68-year-old demented PD patient led to the emergence of visual hallucinations that resolved upon discontinuation of fluoxetine [217].

One nonrandomised, single-blind, pilot study evaluated the effect of fluoxetine (20 mg p.o. id) on orthostatic hypotension in 14 PD patients with orthostatic hypotension. After one month of treatment, there was a significant reduction of the decrease in systolic blood pressure upon standing [218].

As mentioned above, the combination of fluoxetine and selegiline worsened Parkinsonism in a single patient [214]. The combination of these two drugs has also been associated with severe adverse effects. Thus, a 72-year-old depressed PD woman developed features of the 5-HT syndrome following the addition of fluoxetine to L-DOPA and selegiline [219]. A 46 year-old-PD woman developed a manic-like episode with shivers and cold sweat when selegiline and fluoxetine (20 mg p.o. id) were added to L-DOPA and bromocriptine [220]. The addition of fluoxetine (20 mg p.o. id) to L-DOPA, bromocriptine, and selegiline caused shivers and cold sweat to a 56-year-old PD woman [220]. These two patients were previously on amitriptyline (50 mg p.o. at bedtime (hs)), which was well tolerated, except for anticholinergic side effects [220]. The addition of fluoxetine to L-DOPA, selegiline, and bromocriptine to a 44-year-old PD woman resulted in a pheochromocytoma-like syndrome, with labile arterial blood pressure and elevation of plasma and urine catecholamines and catecholamine metabolites [221]. However, the combination of fluoxetine and selegiline in PD patients appears to be generally safe and well tolerated. Thus, in a series of 23 PD patients taking the two drugs concomitantly, no adverse effects other than those associated with each of the two drugs taken individually were reported [222].

An EBM review published by the MDS in 2011 stated that there was “insufficient evidence” regarding the efficacy of fluoxetine for the treatment of depression in PD to make any recommendation [155].

### 4.7. Fluvoxamine

Fluvoxamine is a potent and selective SERT inhibitor which displays moderate/weak affinity for both DAT and NET (Table 1). Fluvoxamine binds with high affinity (Kd = 36 nM) to *σ*_1_ receptors [129]. Fluvoxamine exhibits weak affinity (EC_50_ > 5.0 *μ*M) for *α*_1_, *α*_2_, and *β* adrenoceptors, 5-HT_2A_, *σ*_2_, and D_2_ receptors [129, 223]. The chemical formula of fluvoxamine is illustrated in Figure 1.

In the 6-OHDA-lesioned rat, monotherapy with fluvoxamine (1, 3, 10, and 30 mg/kg i.p.) did not induce rotational behaviour. When combined to the DAT inhibitor vanoxerine, fluvoxamine increased the number of rotations induced by vanoxerine as monotherapy (see Section 7.4) [224]. In an article published in Japanese, fluvoxamine worsened parkinsonian disability, in the MPTP-lesioned marmoset [225].

A case-report study suggested that SSRI-induced Parkinsonism might herald future development of PD. Thus, a 67-year-old woman with depression was administered fluvoxamine (50 mg p.o. bid and then 100 mg p.o. bid), after which she developed a Parkinsonian phenotype, which resolved upon discontinuation and replacement with maprotiline (75 mg p.o. id). However, after 11 months of being asymptomatic, she developed Parkinsonism and was finally diagnosed with idiopathic PD [226]. In another case-report, a 63-year-old depressed man with PD was treated with electroconvulsive therapy and was discharged with fluvoxamine (100 mg p.o. id), but depression relapsed while under fluvoxamine treatment. No mention was made of the effect of fluvoxamine on motor symptoms [227].

In a case-report study, fluvoxamine (50 mg p.o. id) was ineffective at treating fibromyalgia symptoms in a 65-year-old L-DOPA-treated PD man; the effect of fluvoxamine on parkinsonism was not reported [228]. In a randomised, double-blind, placebo-controlled *N*-of-one trial, a 55-year-old PD patient with depression was treated with fluvoxamine (100 mg p.o. id). Both the HDRS and the BDI were improved. Despite no change in the UPDRS part III subscore, the patient felt better and the total daily dose of L-DOPA was reduced by 25% [229]. In a case-report study, fluvoxamine improved a hypokinetic-rigid syndrome in a 64 year-old man. However, because dopaminergic therapy had previously failed to improve the parkinsonian symptoms of the patient and as he was also suffering from severe orthostatic hypotension, the diagnosis of idiopathic PD can be questioned [230].

Despite these two case-reports in which fluvoxamine improved parkinsonian symptoms, there are case-reports in which fluvoxamine worsened Parkinsonism. Thus, a 61-year-old man experienced worsening of bradykinesia, rigidity, and freezing following the introduction of fluvoxamine (titrated up to 100 mg p.o. id over two weeks). In another case-report, a 62-year-old man with PD experienced increases in bradykinesia, rigidity, and gait difficulties following the introduction of fluvoxamine (titrated up to 150 mg p.o. id over two weeks) [231]. Additionally, deterioration of Parkinsonism and psychotic symptoms occurred four days following the introduction of fluvoxamine (25 mg p.o. id) in a 71-year-old man with PD [232].

In another case-report study, the addition of fluvoxamine (50 mg p.o. id) to L-DOPA and tolcapone in a 71-year-old PD man caused the emergence of a 5-HT syndrome 48 hours after the first dose of the antidepressant [233].

### 4.8. Imipramine

Imipramine is a selective SERT inhibitor that binds with moderate affinity to both DAT and NET (Table 1). Imipramine displays high affinity for *β*, 5-HT_2A_, 5-HT_1A_, H_1_, M, and *σ*_1_ receptors and exhibits moderate affinity for *σ*_2_ receptors (Kd of 0.31, 0.34, 2.24, 26, 85, 343, and 2,107 nM, resp.) [129, 234–237]. The chemical formula of imipramine is depicted in Figure 1.

In the 6-OHDA-lesioned rat, imipramine (10 mg/kg i.p.) improved the performance at the forced swim test, a model of depression-like behaviour [238].

The binding of [^3^H]-imipramine in thrombocytes of PD patients was assessed. Binding levels were significantly lower in depressed PD patients than in healthy controls, but no difference was found between depressed and nondepressed PD patients or between nondepressed PD patients and controls [239]. Another study, however, found decreased [^3^H]-imipramine binding levels in the thrombocytes of PD patients compared to age-matched normal individuals [240], whereas another one did not find any difference between PD patients and normal controls [241]. Another study employing [^3^H]-imipramine found reduced binding levels in the putamen of PD patients [242]. [^3^H]-Imipramine binding levels were also reduced in the putamen and prefrontal cortex of PD patients when compared to normal individuals [243].

In a case-report, a 69-year-old PD patient with depression was put on imipramine (125 mg p.o. id) as monotherapy. Imipramine improved tremor and depressive symptoms [244].

In a case-series of 12 patients with postencephalitic, vascular, or idiopathic PD, imipramine (50–150 mg p.o. id) had a favourable effect on parkinsonian features [245]. Imipramine also exerted a favourable effect on Parkinsonism and depressive symptoms in a small case-series of 3 PD patients [246]. In a case-series of 6 depressed PD patients, imipramine as monotherapy improved depression in 5 subjects and had no effect on tremor and bradykinesia [247]. In a case-series of 21 PD patients with depression, imipramine (various doses) alleviated depression in the majority of patients, without deteriorating Parkinsonism [248]. Imipramine also alleviated depression in a case-series 8 PD patients, without worsening parkinsonian disability [249].

In a four-month randomised, cross-over, double-blind, placebo-controlled trial, 70 patients with Parkinsonism (5 with a history of encephalitis, 10 with cerebrovascular disease, and 55 with idiopathic PD) were administered imipramine (up to 200 mg p.o. id). Imipramine led to an improvement of depression in 60% of patients, improved akinesia in 54% of patients, rigidity in 42%, tremor in 28%, and hypersalivation in 57% [250]. In a randomised, double-blind, placebo-controlled study, imipramine (50 mg p.o. bid to tid) was administered as monotherapy to 8 patients with Parkinsonism. Imipramine worsened tremor in one woman with postencephalitic PD and produced no effect in two others. The 5 remaining patients (4 with idiopathic PD and one with postencephalitic PD) were improved. One patient experienced a sialorrhoea reduction, whereas one bedridden patient became able to sit and one wheelchair-bound patient became able to walk [251]. In a case-series of postencephalitic (*N* = 11) and idiopathic (*N* = 13) PD patients, imipramine as monotherapy variably improved motor and nonmotor aspects of parkinsonism. The efficacy of the drug for specific symptoms was different from patient to patient [252].

In an open-label, add-on study performed in 66 L-DOPA-untreated PD patients, 43 patients were improved by imipramine. Of these, 14 noted an improvement of depressive symptoms. Eight patients were on imipramine monotherapy and 6 of these experienced an improvement in Parkinsonism [253]. In an open-label, non-randomised, uncontrolled trial, 10 PD patients were administered imipramine (25–50 mg p.o. tid) with and without trihexyphenidyl. The majority of patients reported some improvement of tremor, rigidity, and bradykinesia following the introduction of imipramine [254]. In a nonrandomised, uncontrolled, open-label study, imipramine (100–250 mg p.o. id) was administered to 15 PD patients as monotherapy. Five patients did not respond to treatment, and rigidity and bradykinesia deteriorated in some of these nonresponders. Five patients improved mildly, mainly in bradykinesia and rigidity, but not in tremor. Five patients were markedly improved and regained some autonomy [255]. In a study of 8 patients with Parkinsonism, imipramine as monotherapy or in combination with trihexyphenidyl led to an improvement of depressive symptoms and rigidity [256].

In a case-series, imipramine (50 mg p.o. tid) was administered to 50 patients with Parkinsonism. 37 patients were improved. Bradykinesia was the most improved symptom. Cases of confusion induced by imipramine were reported [257]. In another case-series, imipramine (100–200 mg p.o. id) was administered to 15 PD patients. Parkinsonism improved in 12 patients. Three patients developed confusion [258–260]. In another case-series, imipramine (30–40 mg p.o. id) was administered to 17 PD patients and improved Parkinsonism in the majority of them [261].

In a 2-month double-blind, placebo-controlled, partly cross-over trial, imipramine (total daily dose of 10–75 mg p.o.) was administered to 32 PD patients, 21 of whom were included in the analysis. Nine received imipramine as monotherapy. No formal statistical analysis was performed, but imipramine was deemed to improve bradykinesia and rigidity but had no effect on tremor [262]. In another study, imipramine (75–150 mg p.o. id) also produced an improvement in tremor and bradykinesia in 12 PD patients [263].

In a nonrandomised, uncontrolled study, dimepramine (50–225 mg p.o. id)—a compound chemically related to imipramine, with undisclosed pharmacological properties that is believed to possess anticholinergic and adrenergic/dopaminergic agonist effects—was administered to 9 patients with parkinsonism (3 with postencephalitic PD and 6 with idiopathic PD). Dimepramine deteriorated cognitive performance and impaired arousal. The drug also decreased autonomic arousal responses, as evaluated by electrodermic skin conduction tests [264].

All of the aforementioned studies were performed with imipramine administered either as monotherapy or in combination with anticholinergic agents. To our knowledge, no study was published in which imipramine was administered with L-DOPA. However, it is possible that imipramine might reduce the efficacy of the anti-Parkinsonian action of L-DOPA, since imipramine interferes with the absorption of L-DOPA at the gastrointestinal level in both rat [265] and human [266].

A study is published in Russian [267] and another one in Danish [268] in which imipramine was used included PD patients; the details of this study will not be reviewed here.

### 4.9. Paroxetine

Paroxetine is a selective SERT inhibitor which binds with high/moderate affinity to DAT and NET (Table 1). Paroxetine also strongly binds to M receptors (Kd = 42 nM) and exhibits moderate affinity at the *α*_1_, *σ*_1_, *α*_2_, and 5-HT_2A_ receptors (Kd of 1.0, 1.9, 3.9, and 6.3 *μ*M, resp.), and low affinity for the 5-HT_1A_, H_1_, and *σ*_2_ receptors (Kd > 10 *μ*M) [129, 233]. The chemical formula of paroxetine is presented in Figure 1.

A study performed in mice suggested that paroxetine may be a neuroprotective agent. Thus, paroxetine (10 mg/kg i.p. id, for 6 days, started 12 hours after the last MPTP injection) significantly attenuated the loss of tyrosine hydroxylase-positive neurons in the SN of mice treated with 4 MPTP injections (total MPTP dose of 20 mg/kg i.p.), each injection being 2 h apart. This regimen of paroxetine also decreased the magnitude of dopamine loss in the striatum, reduced microglial activation, and diminished the production of oxidative agents within the SN [269].

In the 6-OHDA-lesioned rat, acute challenges of paroxetine (0.3, 0.5, and 1.25 mg/kg i.p.) significantly reduced the severity of AIMs and rotational behaviour, without impairing the anti-Parkinsonian action of L-DOPA [134]. In another study,* de novo* treatment with paroxetine (0.5 and 1.25 mg/kg s.c.) reduced the development of L-DOPA-induced AIMs, in the 6-OHDA-lesioned rat [136, 137]. In another study, paroxetine (6 mg/kg i.p.) did not improve the anhedonic behavioural deficits following 6-OHDA lesion in the rat [270, 271].

A study employing [^3^H]-paroxetine found reduced binding levels in the putamen of PD patients [242], whereas another did not find any difference in binding levels in the orbitofrontal and temporal cortices of PD patients when compared to controls [272].

In an article published in Japanese, administration of paroxetine (10 mg p.o. id) to a 73-year-old woman with PD treated with pramipexole led to the development of a neuroleptic malignant syndrome [273]. Paroxetine (20 mg p.o. id) caused visual hallucinations when administered to a 79-year-old woman with PD treated with L-DOPA [274–276].

In a nonrandomised, open-label, single-blind study, paroxetine (started at 5 mg p.o. id and increased up to 20 mg p.o. id) significantly improved depressive symptoms (BDI and HDRS) over a six-month period, without worsening UPDRS part III subscore. One patient experienced worsening of tremor [277]. In a nonrandomised, open-label, tolerability study, paroxetine (10–20 mg p.o. id for three months) significantly improved the HDRS score in 52 PD patients with depression. Two patients reported an increase in the severity of motor symptoms [278].

In a 2-year study, 45 severely depressed PD patients over 60 years of age were treated with paroxetine (20 mg p.o. id). Over 85% of patients showed a good response with few side effects [279]. In a 6-month study performed in 30 depressed PD patients, paroxetine (20 mg p.o. bid) reduced both anxiety and somatic complaints associated with depression [280].

In a randomised, cross-over, double-blind, placebo-controlled i.v. L-DOPA study, paroxetine (started two weeks prior to the evaluation) did not impair L-DOPA anti-Parkinsonian efficacy or alter dyskinesia severity during the i.v. infusion. Paroxetine significantly increased the walking speed during the off-state [281].

In a case-report study, paroxetine (20 mg p.o. id) was ineffective at treating fibromyalgia symptoms in a 65-year-old L-DOPA-treated PD man. No reports were made concerning the effects of paroxetine on Parkinsonism [228]. In another case-report, a 35-year-old PD woman with depression was prescribed paroxetine (20 mg p.o. id). Following one month of therapy, her motor symptoms had deteriorated. Withdrawal of paroxetine led to an improvement of Parkinsonism [282].

Three PD patients were included in an open-label study assessing overactive bladder due to neurological disorders. Paroxetine (40 mg p.o. id) had no effect on urinary symptoms. The authors did not mention the effect of paroxetine on Parkinsonism [283].

A randomised, double-blind, placebo-controlled study conducted in 52 depressed PD patients compared the antidepressant efficacy of paroxetine (12.5–37.5 mg p.o. id) and nortriptyline (25–75 mg p.o. id). Both active treatments improved somatic anxiety and lack of interest after 8 weeks of treatment [96, 284]. Twenty patients entered a 4-month extended phase, in which both active treatments improved cognitive parameters such as verbal memory and word recall [284]. Another publication using the same patients reported that active treatment improved quality of life and did not impair motor function [285].

The Study of Antidepressants in Parkinson's Disease (SAD-PD) was a randomised, double-blind, placebo-controlled, Phase III trial comparing the efficacy of paroxetine (10–40 mg p.o. id) and venlafaxine extended release (37.5–225 mg p.o. id) in 115 depressed PD patients. Both paroxetine and venlafaxine improved the HDRS score to a greater extent than placebo, without exerting deleterious effect on Parkinsonism [97, 286]. A secondary analysis of the SAD-PD study found that high pretreatment depression scores and low pretreatment anxiety scores are the most important prognostic factors for improvement upon antidepressant treatment in PD [287].

An EBM review published by the MDS in 2011 stated that there was “insufficient evidence” regarding the efficacy of paroxetine for the treatment of depression in PD to make any recommendation [155].

### 4.10. R-MDMA

Detailed discussion about the pharmacology and behavioural effects of 3,4-methylenedioxymethamphetamine (MDMA) and its two enantiomers (R- and S-MDMA) is performed in the “MDMA, R-MDMA, and S-MDMA” section (see below). Briefly, R-MDMA a SERT-selective MAUI with additional actions to antagonise 5-HT_2A_ receptors significantly reduced the severity of L-DOPA-induced dyskinesia and psychosis-like behaviours in the MPTP-lesioned common marmoset. However, the mechanism is probably more related to 5-HT_2A_ receptor blockade than SERT inhibition, given the relative affinity of R-MDMA for the two targets. The chemical formula of R-MDMA is presented in Figure 1.

### 4.11. Sertraline

Sertraline is a selective SERT inhibitor that also exhibits high/moderate affinity for DAT and NET (Table 1). Sertraline also has high affinity for *α*_1_, *σ*_1_, *α*_2_, and M receptors (Kd of 36, 57, 477, and 232 nM, resp.) [129, 288]. Sertraline displays moderate affinity for 5-HT_2A_, 5-HT_1A_, H_1_, and *σ*_2_ receptors (Kd of 2.2, 3.7, 5.0, and 5.3 *μ*M, resp.) [129, 288]. The chemical formula of sertraline is illustrated in Figure 1.

In the 6-OHDA-lesioned rat, treatment with sertraline (1.0 and 2.0 mg/kg i.p.) significantly reduced catalepsy at 60, 120, and 180 min after administration, assessed by the time rats spent on a rod. This anticataleptic effect was reversed by preadministration of the 5-HT_1A_ and *α*_2_ antagonist NAN-190 [289, 290].

In the MPTP-lesioned common marmoset not primed to exhibit dyskinesia, sertraline (10 mg/kg p.o.) significantly reduced the activity counts when compared to vehicle-treated animals and slightly worsened parkinsonian disability (5.0 mg/kg p.o., but not 10 mg/kg p.o.). However, sertraline (1.0 mg/kg p.o.) had no effect on motor activity and significantly (although mildly) improved parkinsonian disability. Combining the NET inhibitor nisoxetine with sertraline (both molecules administered at the dose of 1.0 mg/kg p.o.) had no effect on motor activity counts but mildly reversed parkinsonian disability. When added to the DAT inhibitor vanoxerine, sertraline reduced the anti-Parkinsonian benefit provided by vanoxerine as monotherapy [291].

In a case-report, sertraline (50 mg p.o. id) improved pseudobulbar crying induced by deep-brain stimulation of the subthalamic nucleus (STN) in a 46-year-old L-DOPA-treated woman with PD. Of note, the woman had undergone left pallidotomy 8 years prior to deep-brain stimulation [292]. In another case-report, sertraline (50 mg p.o. id) alleviated pseudobulbar laughter following right-sided gamma-knife thalamotomy in a 46-year-old man with PD [293]. In a case-report, sertraline possibly unmasked idiopathic PD. Thus, an 81-year-old woman with depression developed Parkinsonism upon administration of sertraline (50–100 mg p.o. id). The parkinsonian syndrome resolved following discontinuation of sertraline but recurred 14 months later, while the patient was not taking any SSRI, though it is not mentioned if the patient was taking other medications at the time of recurrence [294, 295]. In another report, sertraline (100 mg p.o. id) induced a parkinsonian syndrome in a 70-year-old man with depression. The Parkinsonism did not disappear following sertraline discontinuation and L-DOPA was introduced. Depression was treated with nortriptyline and trazodone (doses not mentioned) [296]. Sertraline (50–100 mg p.o. id) also caused a Parkinsonian syndrome in a 73-year-old depressed woman; whether symptoms resolved completely following withdrawal is unclear [297]. Sertraline (75 mg p.o. id) improved speech in a 52-year-old PD patient who had stuttering during the on-off transitions [298]. A 76-year-old man with PD, treated with L-DOPA and amantadine developed a 5-HT syndrome when sertraline (50 mg p.o. id) was added to his medication [299].

Sertraline (50 mg p.o. id) successfully alleviated depression in a 61-year-old man with PD treated with L-DOPA and selegiline [300].

In a pilot, open-label, nonrandomised, unblinded, 7-week study, the safety and efficacy of sertraline (25–50 mg p.o. id) were assessed in 15 depressed PD patients. Sertraline significantly improved the BDI score and did not worsen the UPDRS part III subscore [301, 302]. Another uncontrolled, open-label, 3-month trial performed in 21 depressed PD patients reported similar findings. In that study, sertraline (50 mg p.o. id) significantly improved the Geriatric Depression Scale score without affecting motor function [303, 304]. In a case series of 5 PD patients, sertraline (50–100 mg p.o. id) significantly improved the Beck Anxiety Inventory (BAI) and the BDI scores [305].

In a 14-week randomised, open-label, single-blind study performed in 67 depressed PD patients, the antidepressant actions of sertraline and pramipexole were compared. Pramipexole (1.5 to 4.5 mg p.o. id) was significantly more effective than sertraline (50 mg p.o. id) at improving the HDRS score [306]. However, the subjective improvement, assessed by the Zung self-rating scale, was similar in both treatment groups. Sertraline slightly improved the UPDRS part III subscore.

In a 6-month open-label, randomised trial, sertraline liquid formulation was compared to sertraline regular formulation in 54 depressed PD patients [307]. Both formulations were equally effective at improving the Turkish-HDRS and Turkish-MADRS scores. None of the formulations affected the UPDRS part III subscore.

In a 3-month randomised, single-blind study, sertraline (50 mg p.o. id) was compared to amitriptyline (25 mg p.o. id) in 31 depressed PD patients [308]. Both drugs significantly improved the HDRS-17 score and none affected the UPDRS part III sub-score.

In an open-label, nonrandomised, 6-month study of 310 community-dwelling PD patients with depression, sertraline (50–200 mg p.o. id) significantly improved the HDRS score as well as all of the subscales of the UPDRS [309]. However, at the end of the study, patients were taking significantly higher L-DOPA doses than at baseline, raising suspicion as to whether sertraline was indeed the cause of the motor improvement.

One randomised, double-blind, placebo-controlled, 10-week trial of sertraline in PD depression was performed. The trial stopped prematurely because of difficulty recruiting patients. Nevertheless, 12 patients were included and sertraline (25–100 mg p.o. id) was not superior to placebo at improving the MADRS score. The UPDRS part III subscore did not differ significantly to pretreatment baseline in any group [310].

A case of probable 5-HT syndrome was reported in a 75-year-old woman taking L-DOPA/carbidopa/entacapone, amantadine and rasagiline 1 mg id, one week after sertraline was increased to 100 mg id [311].

### 4.12. Trazodone

Although trazodone is included in this review as a MAUI, it has higher affinity for the *α*_1_, 5-HT_2A_, H_1_, 5-HT_1A_, and *α*_2_ receptors (Kd of 12, 20, 29, 42, and 106 nM, resp.) [288] than for any of the monoamine transporters. Trazodone exhibits high affinity for SERT and moderate/weak affinity for DAT and NET (Table 1). It is thus likely that most of the biological effects of trazodone come from its interaction with the aforementioned receptors rather than a monoamine reuptake inhibition. The chemical formula of trazodone is presented in Figure 1.

In a 5-month randomised, single-blind, uncontrolled trial, trazodone (50 mg p.o. id) significantly improved the HDRS score in 8 depressed PD patients. The UPDRS part III subscale was not worsened by the use of trazodone. The effect of trazodone on dyskinesia was not reported [312]. However, there is one case-report in which trazodone (25–125 mg p.o. id) was successful at alleviating dyskinesia in a 61-year-old depressed PD man [313].

The efficacy of trazodone for PD tremor is unclear. Thus, in a study performed in 26 PD patients, trazodone (150 mg p.o. id) was beneficial for tremor, but the benefit decreased with time [314]. In a 30-day randomised, double-blind, placebo-controlled trial encompassing 19 PD patients, trazodone (100 mg p.o. bid) failed to provide any benefit for tremor [315]. In contrast, in a case-series conducted in 27 PD patients, trazodone (single challenge of 0.71 mg/kg i.v.) improved tremor in 9 PD patients [316–318]. In another case-series, trazodone (300 mg p.o. id for 30 days) improved tremor in 2 out of 10 PD patients, when added to L-DOPA [319].

In a case presented as an abstract, a 68-year-old woman with PD taking trazodone (150 mg p.o. id) suffering from obstructive sleep apnoea saw an improvement of her sleep following STN deep-brain stimulation [320].

### 4.13. Trimipramine

Trimipramine is a selective SERT inhibitor displaying moderate affinity for the NET and DAT (Table 1). Its chemical formula is presented in Figure 1 [321]. Trimipramine also exhibits high affinity for H_1_, 5-HT_2A_, *α*_1_, D_2_, M, *α*_2B_, D_1_, and 5-HT_2C_ (Kd of 1.4, 19.5, 24, 57.5, 59, 280, 347, and 537 nM, resp.) receptors and moderate affinity for *α*_2A_ and 5-HT_3_ receptors (Kd of 1.38 and 9.12 *μ*M, resp.) [321]. To our knowledge, only one article reporting the use of trimipramine in PD has been published. In that case-report, trimipramine (50 mg p.o. id) was ineffective at alleviating anxiety in a 39-year-old L-DOPA-treated man with PD for 6 years. The effect of trimipramine on Parkinsonism was not reported [322].

### 4.14. UWA-122

Detailed discussion about the pharmacology and behavioural effects of UWA-101 and its two enantiomers (UWA-121 and UWA-122) is performed in the “UWA-101, UWA-121, and UWA-122” section (see below). Briefly, UWA-122, a SERT-selective MAUI, did not produce any effect in combination with L-DOPA in the MPTP-lesioned common marmoset. The chemical formula of UWA-122 is illustrated in Figure 1.

### 4.15. Venlafaxine

Venlafaxine has high affinity for SERT and high/moderate affinity for NET and DAT (Table 1). Venlafaxine exhibits moderate affinity at 5-HT_4_, 5-HT_2C_, 5-HT_2A_, and 5-HT_6_ receptors (Kd of > 1.0, 2.0, 2.2, and 2.8 *μ*M, resp.) [165]. Venlafaxine has weak affinity for H_1_, M, and *α*_1_ receptors (Kd of 12.9, 30.0, and 39.9 *μ*M, resp.) [288]. The chemical formula of venlafaxine is presented in Figure 1.

In a study of 29 depressed PD patients, venlafaxine (75 mg p.o. id) significantly improved the BDI and HDRS scores after 8 weeks of treatment. Venlafaxine did not modify the UPDRS score [323].

The SAD-PD study was a randomised, double-blind, placebo-controlled, Phase III trial comparing venlafaxine (37.5–225 mg p.o. id) to paroxetine (10–40 mg p.o. id). In that study, both venlafaxine and paroxetine reduced the HDRS score, without worsening motor function [97, 286].

A case of 5-HT syndrome has been described in a 43-year-old man with PD taking venlafaxine (75 mg p.o. id), L-DOPA, ropinirole, amantadine, and benzhexol. Venlafaxine was started two weeks prior to development of the 5-HT syndrome [324]. In a case-report, venlafaxine (150 mg p.o. bid) triggered spontaneous erections and increased libido in a 58-year old PD patient. The effect of venlafaxine on Parkinsonism was not mentioned [325].

To our knowledge, no study examining the effects of desvenlafaxine (O-desmethylvenlafaxine), the major active metabolite of venlafaxine [326], in PD or animal models of PD has been published.

### 4.16. SERT Inhibitors: Summary

The following SERT-selective MAUIs have been used in studies in PD and/or related animal models: citalopram, clomipramine, duloxetine, escitalopram, fenfluramine, fluoxetine, fluvoxamine, imipramine, paroxetine, R-MDMA, sertraline, trazodone, trimipramine, UWA-122, and venlafaxine. The results of the studies involving these SERT inhibitors, excepting R-MDMA and UWA-122, are summarised in Table 3.

In weighing evidence based on quality of data, we conclude that selective SERT inhibitors

1. could be effective against L-DOPA-induced dyskinesia;
2. may impair L-DOPA anti-Parkinsonian action, although this is inconsistent throughout studies, usually minor when it happens and can be alleviated by increasing L-DOPA dose;
3. are probably effective for anxiety and depression.

Indeed, in the majority of the studies cited above, SERT-selective MAUIs were effective against both anxiety and depression. However, SSRIs may not be the best molecules to employ if a rapid antidepressant effect is needed, as selective NET inhibitors appear to have a quicker onset of therapeutic benefit.

The potential efficacy of selective SERT MAUIs in PD appears to extend beyond their traditional role as antidepressants and anxiolytics. Thus, as discussed above, chronic treatment with citalopram and acute challenges of fenfluramine effectively alleviated L-DOPA-induced AIMs in the 6-OHDA-lesioned rat model of PD and, in the settings of a clinical trial, fluoxetine reduced apomorphine-induced dyskinesia in PD patients. The antidyskinetic potential of SERT inhibitors certainly requires further exploration in well-controlled clinical trials whose primary end-point would be the assessment of dyskinesia severity. Eventual clinical trials evaluating the antidyskinetic potential of SSRIs should also assess their effect on L-DOPA anti-Parkinsonian action as a secondary end-point, as the issue remains controversial despite the abundant literature reviewed here. However, as discussed above, an indirect interaction with 5-HT_1A_ and 5-HT_2C_ receptors via an increase in 5-HT levels after SERT inhibition could account for the antidyskinetic potential of the compounds, by reducing dopamine release, a phenomenon that might also reduce L-DOPA anti-Parkinsonian action. We propose that patients with more severe nigrostriatal lesion, that is, more advanced disease, might be more susceptible to experiencing a deterioration of Parkinsonism upon treatment with a SERT-selective MAUI. Additionally, in order to produce a therapeutic effect without worsening Parkinsonism, it might be necessary to administer specific doses of medication, as some SSRIs appear to have very narrow therapeutic window in the context of Parkinsonism. This is well-exemplified by sertraline which worsened the parkinsonian phenotype at 10 mg/kg but improved it at 1 mg/kg, when administered as monotherapy to the MPTP-lesioned marmoset. Lastly, the potential neuroprotective effects of fluoxetine and paroxetine against MPTP-induced neurotoxicity are interesting findings that require further exploration. Similarly, fluoxetine-induced enhancement of cellular proliferation in the SGZ requires further characterisation, as it could lead to new therapies for PD,* a fortiori* as SSRIs are highly prescribed, well-characterised, and well-tolerated molecules with a well-documented adverse-effect profile.

## 5. SERT = NET Inhibitors

### 5.1. Amitriptyline

Amitriptyline has high affinity for SERT and NET and moderate affinity for DAT (Table 1). Amitriptyline also binds with high affinity to H_1_, M, *α*_1_, 5-HT_2A_, *α*_2_, and 5-HT_1A_ receptors (Kd of 0.17, 2.6, 4.4, 5.3, 114, and 129 nM, resp.) [235, 288]. Amitriptyline is also a potent 5-HT_2C_ inverse agonist (EC_50_ of 235 nM) [371] and binds with high affinity to the *σ*_1_ receptors (Kd of 287 nM) [372]. In addition amitriptyline inhibits with moderate affinity the MAO-B (Kd of 8.4 *μ*M) [373] and the butyrylcholinesterase (Kd of 10 *μ*M) [374]. Under normal conditions, amitriptyline also has weak affinity for the *N*-methyl-D-aspartate (NDMA) glutamate receptors (EC_50_ of 20 *μ*M) [375]. Amitriptyline inhibits the delayed potassium rectifier potassium channels Kv7.2/7.3 and Kv1.1 (EC_50_ of 1.0 and 22 *μ*M, resp.) [376]. Additionally, amitriptyline depresses sodium current (Kd of 20 *μ*M) [377], as well as L-type calcium current in the heart (EC_50_ of 23 *μ*M at a stimulation frequency of 0.33 Hz) [378]. Lastly, amitriptyline is a TrkA and TrkB neurotrophin receptor agonist, thereby exerting neurotrophic activity [379]. The chemical formula of amitriptyline is displayed in Figure 2.

In the 6-OHDA-lesioned rat, amitriptyline protected against 6-OHDA toxicity and increased BDNF levels in the intact and lesioned SN [119, 380, 381]. Chronic amitriptyline treatment also increased BDNF levels in the hippocampus but decreased them in the striatum, anterior cingulate, and pyriform cortex contralateral to the injection site [119]. Levels of BDNF were also increased after amitriptyline administration in the hippocampus ipsilateral to the lesion [119]. Amitriptyline treatment also increased glial cell line-derived neurotrophic factor (GDNF) levels in the contralateral hippocampus, anterior cingulate cortex and SN [119].

In a study published as an abstract conducted in the 6-OHDA-lesioned rat, amitriptyline (7.5, 15 or 30 mg i.p.) was administered to rats, but its effects were not reported [162].

In a case-report study, amitriptyline (30 mg p.o. id) was ineffective at treating fibromyalgia symptoms in a 65-year-old L-DOPA-treated PD man. No report was made concerning the effects of amitriptyline on Parkinsonism [228]. In a case-report published in Japanese, a 59-year-old PD patient with hypothyroidism developed a neuroleptic malignant syndrome following discontinuation of amitriptyline and the benzodiazepine analogue etizolam [382]. In another case-report, amitriptyline (10 mg p.o. hs) improved off-period depression and sleep disturbance in a 59-year-old woman with PD [383]. Administration of amitriptyline (25 mg p.o. hs) improved restless syndrome in a 63-year old man with PD [384]. A 63-year-old woman with PD on amitriptyline (20 mg p.o. hs) developed hypertensive crisis following the introduction of L-DOPA and metoclopramide [385]. An 82-year-old PD patient (sex not mentioned) also developed a hypertensive crisis while on L-DOPA and imipramine (25 mg p.o. tid); following the discontinuation of imipramine and introduction of amitriptyline (25 mg p.o. tid), the patient also developed a hypertensive crisis [386]. Amitriptyline (dose not mentioned) improved depressive symptoms in a 65-year-old man with PD [387, 388]. Amitriptyline also improved depressive symptoms in two PD patients, a 62-year-old man (25 mg p.o. tid, reduced to 10 mg p.o. qid after two weeks) and a 75-year-old man (25 mg p.o. tid) [389]. In a case-series, the addition of amitriptyline (dose not mentioned, but individually tailored for each patient and lower than 20 mg p.o. id, administered for 30 days) to L-DOPA exerted a beneficial effect on tremor in 5 out of 10 patients [319].

In a 16-week randomised, placebo-controlled, double-blind study, the efficacy of amitriptyline (25 mg p.o. bid) against muscle contraction headache was evaluated in 31 PD patients. Four weeks after the beginning of the study, patients in the amitriptyline group had significantly less days of headache per month and were taking less analgesics. These differences remained throughout the study. The Zung Depression Scale and the Webster Rating Scale scores both remained unchanged [390].

In a randomised, single-blind, uncontrolled, one year study, amitriptyline (25–75 mg p.o. id) was compared to fluoxetine (20–40 mg p.o. id) in 77 depressed PD patients. 19 patients dropped out of the study, all in the amitriptyline group, because of adverse effects. However, amitriptyline was significantly more effective than fluoxetine at controlling depressive symptoms, as measured with the HDRS. No worsening of the UPDRS score was reported [391].

A one-year randomised, double-blind, uncontrolled study compared amitriptyline (mean dose of 69 mg p.o. id; *N* = 27 patients) to fluvoxamine (mean dose of 78 mg p.o. id; *N* = 20 patients) in depressed PD patients. Seven patients in the fluvoxamine and 10 in the amitriptyline group had to cease the study because of confusion and visual hallucinations. One patient in the fluvoxamine group experienced an exacerbation of tremor. Both drugs were equally effective at alleviating depressive symptoms [392].

In its 2006 practice parameters for the evaluation and treatment of depression, psychosis, and dementia in PD, the AAN states that “amitriptyline may be considered to treat depression in PD without dementia,” level C evidence [393]. On the other hand, an EBM review published by the MDS in 2011 stated that “there was insufficient evidence for amitriptyline to be rated for the treatment of depression in PD” [155].

The use of amitriptyline in PD is also reported in the Swedish [394] and German medical literature (no translation or English abstract provided) [395].

### 5.2. Milnacipran

Milnacipran (or midalcipran or F 2207) is a dual SERT/NET inhibitor (Table 1) which exerts virtually no effects (EC_50_ > 10 *μ*M) at the *α*_1_, *α*_2_, *β*, 5-HT_1_, 5-HT_2_, D_2_, H_1_, M, and benzodiazepine receptors [355]. The chemical formula of milnacipran is displayed in Figure 2.

In a case-report study, milnacipran (30 mg p.o. id) was ineffective at treating fibromyalgia symptoms in a 65-year-old L-DOPA-treated PD man. No comments were made concerning the effects of milnacipran on Parkinsonism [228].

In a series of 2 case-reports, milnacipran was effective at alleviating depressive symptoms in PD [396]. The first case was a 62-year-old man with PD on L-DOPA who had depressive symptoms that were unresponsive to paroxetine (40 mg p.o. id). Paroxetine was switched for milnacipran (100 mg p.o. id), which resulted in improvement of the depression. No changes were noted on the motor symptoms. The second case was a 64 year-old woman with PD on L-DOPA and trihexyphenidyl whose depressive symptoms were unresponsive to fluvoxamine (200 mg p.o. id). Fluvoxamine was replaced by milnacipran (100 mg p.o. id) which improved the depressive symptoms. No motor fluctuations were reported.

In an open-label, nonrandomised, uncontrolled, unblinded trial, 8 depressed PD patients were administered milnacipran (30 mg p.o. bid for 12 weeks). Seven patients completed the trial and milnacipran significantly improved the HDRS score without affecting the motor function [397].

In an open-label study assessing overactive bladder due to neurological disorders, three PD patients were included [283]. Milnacipran (100 mg p.o. id) significantly improved bladder capacity when all of the patients of the study were considered, but the effect of the drug on the urinary function of the PD subpopulation was not mentioned. The authors did not comment about PD motor and nonmotor symptoms either.

### 5.3. SERT = NET Inhibitors: Summary

The following SERT = NET MAUIs have been used in studies in PD and/or related animal models: amitriptyline and milnacipran. The results of the studies involving SERT = NET inhibitors in PD are summarised in Table 4.

In weighing evidence based on quality of data, we conclude that mixed SERT = NET inhibitors

1. are probably effective for depressive symptoms in PD;
2. do not exert a deleterious effect on L-DOPA anti-Parkinsonian efficacy.

Other potential benefits of SERT = NET inhibitors in PD relate to an analgesic effect, as evidenced by a reduction in muscle contraction headache, as well as a potential efficacy for overactive bladder. However, the reports against these symptoms are anecdotal and require further confirmation. An interesting effect of amitriptyline lies in its capacity to increase levels of the neurotrophic factors BDNF and GDNF in the SN of the 6-OHDA-lesioned rat. This is a finding that might have important implications, as it might lead to disease-modifying/neurorestorative treatments. It remains to be demonstrated if treatment with amitriptyline also leads to such elevations in neurotrophic factor levels in the MPTP-lesioned primate and in idiopathic PD.

## 6. NET Inhibitors

### 6.1. Amoxapine

Amoxapine is a selective NET inhibitor that exhibits high affinity at SERT and moderate affinity at DAT (Table 1). Amoxapine also binds to H_1_, *α*_1_, D_2_, M, and *α*_2_ receptors (Kd of 25, 50, 160, 1,000, and 2,600 nM, resp.) [398]. Amoxapine acts as an antagonist at D_2_ receptors and could potentially worsen parkinsonian disability [399]. Amoxapine also exhibits affinity for 5-HT_2C_, 5-HT_2A_, and 5-HT_1A_ receptors (Kd of 4.3, 8.5, and 1,995 nM, resp.) [400]. The chemical formula of amoxapine is depicted in Figure 3.

In a study of 3 case-reports, amoxapine (12.5–100 mg p.o. id) improved visual hallucinations and reduced dyskinesia severity in two PD patients. Motor function—especially off-time duration—was worsened in all 3 patients [401].

### 6.2. Amphetamine, Methamphetamine, and Propylhexedrine

Amphetamine and its L-enantiomer, levoamphetamine (L-amphetamine), are selective NET inhibitors that exhibit high/moderate affinity for DAT and SERT (Table 1). Dextroamphetamine (D-amphetamine) is a dual NET/DAT inhibitor that exhibits moderate/weak affinity for SERT (Table 1). D-Amphetamine also binds to M_1_ and *σ* receptors (Kd of 36.2 nM and 12.7 *μ*M, resp.) [402, 403]. Upon binding to a transporter, amphetamine inhibits its uptake function and reverses its action, thereby increasing monoamine release [347]. Unlike amphetamine, methamphetamine is a mixed DAT = NET inhibitor (Table 1) but, like amphetamine, methamphetamine enhances monoamine release, in addition to inhibiting monoamine reuptake [347]. Methamphetamine also binds to *σ*_1_ and *σ*_2_ receptors (Kd of 2.2 and 47 *μ*M, resp.) [404]. Propylhexedrine is a compound structurally related to amphetamine and methamphetamine. Despite an extensive search, the precise pharmacological profile of propylhexedrine could not be found, but it appears to act as amphetamine and methamphetamine [405, 406]. The chemical formulae of amphetamine, L-amphetamine, and (+)-methamphetamine (selective NET inhibitors) are illustrated in Figure 3, whereas the chemical formulae of D-amphetamine, methamphetamine, and (−)-methamphetamine (dual DAT = NET inhibitors) are depicted in Figure 5. Although some of the aforementioned compounds are selective NET inhibitors and some are dual DAT = NET inhibitors, they are all discussed in the current section as they are amphetamine-derivatives. To our knowledge, no studies were performed with the enantiomers of methamphetamine in PD. Their affinities for each of the monoamine transporters and their chemical formulae are nevertheless included in this review article, to provide a better understanding of racemic methamphetamine.

Several studies performed in the rodent and nonhuman primate have demonstrated striatal dopaminergic denervation following the administration of amphetamine and methamphetamine, raising the possibility of neurotoxicity of these compounds [407–414]. Based on studies performed in mice, methamphetamine toxicity does not seem to affect catecholaminergic neurons of the gastrointestinal tract [415] or of the heart [416]. In a PET study conducted in human subjects, DAT binding was significantly reduced in the striatum of former methamphetamine users, when compared to controls [417]. This reduction of DAT binding persisted even after discontinuation of methamphetamine [418]. In a* postmortem* human study performed in the brains of 12 chronic methamphetamine users, dopamine levels were significantly reduced in the caudate and putamen of methamphetamine users, when compared to controls [419]. Accordingly, epidemiological studies found that the risk of developing PD was greater in methamphetamine users than in nonusers [420–422]. Amphetamine exposure also appears significantly more frequent in PD patients than in age-matched controls [422–424]. Moreover, use of stimulants such as amphetamine, MDMA, and cocaine can result in SN hyperechogenicity, as in PD [425]. The Parkinsonian features in the amphetamine-exposed PD patients are not different to those of non-amphetamine-exposed PD patients, but Parkinsonism tends to start at a younger age in the methamphetamine-exposed group [426, 427].

In the 6-OHDA-lesioned rat, amphetamine injection does not increase dopamine or DOPAC levels and does not lead to an increase in OH^−^ levels in the denervated striatum [428]. Amphetamine does not improve the deficit at the forelimb stepping test, which is impaired in the rat after 6-OHDA injection [429]. D-Amphetamine causes rotations towards the lesioned side [430, 431]. In contrast to apomorphine, D-amphetamine-induced rotations occur at lesser degrees of striatal dopamine depletion, suggesting that apomorphine is more sensitive than D-amphetamine to detect severe striatal dopamine denervation [432, 433]. D-Amphetamine causes rotations by enhancing dopamine levels in the intact striatum, whereas apomorphine stimulates dopamine receptors on both the lesioned and unlesioned sides, but dopamine receptor supersensitivity on the lesioned side leads to contralateral rotations [434, 435].

D-Amphetamine elicited AIMs when L-DOPA-primed 6-OHDA-lesioned mice [436] and rats were grafted with intrastriatal transplantation of embryonic ventral mesencephalon [437–442] or intrastriatal transplantation of foetal dopaminergic and serotonergic neurons [443]. Like D-amphetamine, methamphetamine induces rotations ipsilateral to the lesioned side in the 6-OHDA-lesioned rat. These rotations are reduced following striatal grafting of embryonic stem cells [174]. Methamphetamine (1 mg/kg i.p.) also induces reward-mediated behaviour, in the 6-OHDA-lesioned rat [444]. Administration of methamphetamine (3 mg/kg i.p.) to 6-OHDA-lesioned rats induced FBJ murine osteosarcoma viral oncogene homolog- (Fos-) like immunoreactivity in the striatum and globus pallidus (GP) contralateral to the lesion and the SN pars reticulata ipsilateral to the lesion [445–447]. Because the amphetamine-induced rotation test has been used in many studies as a way to determine the degree of striatal dopamine denervation of the animals and not as a study endpoint* per se*, these studies are not reviewed here. Of note, amphetamine administration also induces rotational behaviour in the 6-OHDA-lesioned hemi-Parkinsonian monkey [448–451], the hemi-MPTP-lesioned monkey [452], but not in the 6-OHDA-lesioned hemi-Parkinsonian Black Silkie chicken [453]. Chronic but not acute administration of amphetamine induces rotations in the hemi-MPTP-lesioned sheep [454].

In the L-DOPA-primed 6-OHDA-lesioned rat transplanted with embryonic dopaminergic neurons within the striatum, administration of D-amphetamine (1.5 mg/kg i.p.) triggered AIMs, the severity of which was increased when fenfluramine (2 mg/kg i.p.) was coinjected with D-amphetamine [176].

Administration of methamphetamine to the 6-OHDA-lesioned rat alters the expression of several genes and transcription factors, such as c-Fos, FosB, and Egr1 [455–457]. In the MPTP-lesioned mouse, a study demonstrated that D-amphetamine-induced motor activity depended on the dose of MPTP administered and on the remaining striatal dopamine content, with severely lesioned animals being less responsive [458].

In the rat, none of D-amphetamine (10 mg/kg s.c.), L-amphetamine (20 mg/kg s.c.), or methylphenidate (20 mg/kg p.o.) attenuated the catalepsy induced by intraventricular injection of 6-OHDA followed, 2 weeks later, by administration of the inhibitor of catecholamine synthesis *α*-methyl-para-tyrosine [459].

In PD, methamphetamine enhances dopamine release by surviving nigrostriatal neurons. For instance, in a PET study performed in 6 PD patients who had been suffering from the disease for at least 7 years, methamphetamine (0.3 mg/kg i.v., administered after an overnight withdrawal of anti-Parkinsonian medication) led to a significant decrease in [^11^C]-raclopride binding when compared to saline injections [460]. Other studies have used this technique to measure dopamine release in PD [461, 462]. As the therapeutic benefit of methamphetamine was not the primary endpoint of these studies, they will not be reviewed here. Methamphetamine (0.3 mg/kg i.v.) induced increases in both diastolic and systolic blood pressure in 11 PD patients [463].

In single-blind, nonrandomised, placebo-controlled study, amphetamine (10–160 mg p.o. id) was administered to 28 patients with postencephalitic PD and 10 patients with vascular PD for 4 weeks to 16 months. No statistical analysis was performed. Although little objective effects were noted, amphetamine-treated patients experienced favourable effects on rigidity, energy levels and activities of daily living in post-encephalitic PD cases. Amphetamine was highly effective at alleviating oculogyric crises. In the cases of vascular PD however, only one patient reported to feel better, whereas 6 felt worse on amphetamine [464]. In a case-series of 20 patients with idiopathic and postencephalitic PD, amphetamine (15–50 mg p.o. id) was administered for a time period of 1 week to 10 months. No statistical analysis was performed. 15 patients were improved, one patient was unchanged, and two patients deteriorated. Oculogyric crises improved in 5 out of 6 patients. Mood improvement was noted in 13 patients. Improvement in rigidity and tremor was inconsistent [465]. In a case-series of 12 patients with postencephalitic PD, amphetamine (10–15 mg p.o. bid) was administered either as monotherapy or in combination with atropine for several days. When combined to atropine, amphetamine improved sleep cycle, increased energy, and improved oculogyric crises. Amphetamine produced little clinical effect when administered as monotherapy [466]. In a case-series, a mixture of amphetamine (5 mg), apomorphine (1.5 mg), strychnine (1 mg), and metrazol (50 mg) per 4 cc was administered (2–8 cc at a time, number of daily intakes not specified) to 63 PD patients and was found to be beneficial to 40, while it had no benefit and was even toxic to 23 [467].

In a case-series, 71 postencephalitic and 3 idiopathic PD patients were administered amphetamine (40–60 mg p.o. id in two doses) as an add-on medication. No statistical analysis was performed. The majority of patients were subjectively improved. Amphetamine was highly effective against oculogyric crises. The grip strength and writing were improved in the majority of patients. The effects on tremor, rigidity, and bradykinesia were inconsistent [468].

In an open-label, nonrandomised, add-on trial, D-amphetamine (5 mg p.o. bid, *N* = 30), or methylphenidate (maximal dose of 10 mg p.o. id, *N* = 21) was administered to L-DOPA-treated PD patients. No statistical analysis was performed. Both drugs had a positive effect on gait, but no improvement was noted on tremor, rigidity, or bradykinesia. Methylphenidate abolished somnolence. In addition, some patients presented depressive symptoms despite treatment with TCAs and L-DOPA; the addition of either D-amphetamine or methylphenidate led to complete resolution of depressive symptoms [469]. D-Amphetamine (5 mg p.o. bid-tid) was also reported to improve akinesia in PD patients (number not mentioned) [260].

In an open-label, nonrandomised, uncontrolled, add-on trial, D-amphetamine (5–10 mg p.o. id) was added to L-DOPA (3-4 grams, administered without an AADC inhibitor) in 9 PD patients. Of these, 7 were later started on imipramine (50–100 mg p.o. id). D-amphetamine, either alone or in combination with imipramine, improved bradykinesia and rigidity in most patients, but was not effective for tremor [470].

In a randomised, double-blind, placebo-controlled, cross-over study, L- (30 mg p.o. am and 20 mg p.o. at midday) and D-amphetamine (10 mg p.o. am and 5 mg p.o. at midday) were administered to 22 PD patients (only 12 patients received D-amphetamine). 15 patients were on L-DOPA and 16 on amantadine. The blind had to be ceased prematurely because of side effects. L-Amphetamine significantly improved total PD disability, tremor, and rigidity, but not bradykinesia and posture. D-Amphetamine did not produce any benefit. Dyskinesia was exacerbated in two patients. Two patients experienced hallucinations and worsening of tremor on L-amphetamine [471].

There are a few reports, in the German literature, on the use of propylhexedrine in PD. They will not be detailed here, but propylhexedrine, as monotherapy, reportedly improved Parkinsonism. Propylhexedrine provided a greater benefit when administered in combination with L-DOPA than as monotherapy [469, 472–476]. Another article published in German without English abstract also reports the use of amphetamine in PD [477].

### 6.3. Atomoxetine

The NET inhibitor atomoxetine (or tomoxetine) also exhibits high affinity at SERT and moderate affinity at DAT (Table 1). In a screening study encompassing more than 60 other binding sites, atomoxetine displayed affinity weaker than 1.0 *μ*M at each of the targets [339]. The chemical formula of atomoxetine is presented in Figure 3.

In a double-blind study presented as an abstract, atomoxetine, administered over 6 weeks to 4 PD patients, did not improve attention when compared to baseline [478]. In a 7-week open-label study, atomoxetine (40 mg p.o. bid) was administered to 10 PD patients, 3 of whom had STN deep-brain stimulation. Atomoxetine was well tolerated, although there was no improvement in the freezing of gait questionnaire. Four of the 10 patients reported subjective improvement of freezing of gait [479].

In a randomised, double-blind, placebo-controlled cross-over study, 25 PD patients were administered atomoxetine 40 mg p.o. or placebo, separated by at least one week. Overall, atomoxetine had no significant effect on impulsivity, measured by deliberation time, stop signal reaction time, and reflexion impulsivity. However, reanalysis of data taking into consideration plasma levels of atomoxetine showed that high plasma concentrations were associated with higher impulsivity, whereas low plasma concentrations were associated with decreased impulsivity, compared to placebo, suggesting that atomoxetine, as a treatment for impulsivity, might have a narrow therapeutic window in the PD population [480–482]. The benefit of atomoxetine seems to be associated with increased right inferior frontal gyrus activation and enhanced fronto-striatal connectivity [481].

In an 8-week open-label, flexible dose (25–100 mg p.o. id, mean 89.6 mg p.o. id), add-on study, atomoxetine was administered to 12 PD patients with executive dysfunction. Atomoxetine significantly improved executive dysfunction. Two patients reported increased dreaming, rigidity, and hyperkinetic movements [483].

In a randomised, double-blind, placebo-controlled, add-on study of 5 PD patients, atomoxetine (40 mg p.o. id, *N* = 3) did not significantly improve freezing of gait. UPDRS part I, II, and III scores were not modified [484, 485].

In a randomised, double-blind, placebo-controlled study, the efficacy of atomoxetine for depression in PD was assessed in 55 patients. 50 were included in the analysis. Atomoxetine did not significantly improve depressive symptoms when compared to placebo after 8 weeks of treatment, but improved cognition and reduced daytime sleepiness [486, 487].

An EBM review published by the MDS in 2011 stated that there was “insufficient evidence” regarding the efficacy of atomoxetine for the treatment of PD to make any recommendation [155].

An observational study assessing the effect of atomoxetine on blood pressure in neurogenic hypotension in PD patients is ongoing [488]. An open-label, non-randomised, uncontrolled safety and efficacy study of atomoxetine in the treatment of executive dysfunction in PD was active, but its current status is unknown [489]. A Phase II randomised, double-blind, placebo-controlled study assessing the effect of atomoxetine for the treatment of cognitive in PD is currently ongoing [490].

### 6.4. Desipramine

Desipramine (desmethylimipramine) is a selective NET inhibitor that displays high affinity for SERT and moderate affinity for DAT (Table 1). The compound also has high affinity for *α*_1_, H_1_, M, and 5-HT_2A_ receptors (Kd of 23, 31, 37, and 115 nM, resp.) and moderate affinity for *α*_2_, *σ*_1_ and 5-HT_1A_ receptors (Kd of 1.4, 2.0, and 2.3 *μ*M, resp.) [129, 288].

Desipramine is frequently administered prior to administration of 6-OHDA, in order to protect noradrenergic neurons from 6-OHDA toxicity, thereby making the lesion selective for dopaminergic neurons [491].

In the MPTP-lesioned mouse, desipramine (0.5, 1.0, 5.0, and 10.0 mg/kg i.p.) significantly reduced the duration of REM sleep and increased duration of slow-wave sleep, when compared to saline-treated mice. A similar effect was seen in normal mice [132].

In a study published as an abstract conducted in the 6-OHDA-lesioned rat, desipramine (7.5, 15, or 30 mg i.p.) delayed the onset of L-DOPA anti-Parkinsonian action [162].

Binding of [^3^H]-desipramine is unchanged in the LC of PD patients, whether they have concomitant dementia or not [492].

In a case series of 7 postencephalitic and one idiopathic PD patients, desipramine (75–150 mg p.o. id as monotherapy) had a beneficial effect on Parkinsonism [493]. In a case series of 7 postencephalitic and 3 vascular PD patients, desipramine (up to 100 mg p.o. id as monotherapy or in combination with anticholinergics) improved Parkinsonism in some patients and had to be stopped in one because of confusion [494]. In a randomised, double-blind, placebo-controlled study, desipramine (total daily dose of 100 mg p.o.) as monotherapy was assessed in 39 patients with Parkinsonism (1 case of hereditary parkinsonism, 6 cases of postencephalitic PD, and 32 cases of idiopathic PD). Desipramine improved depressive symptoms in 9 patients, rigidity in 5, and tremor in 3 [495]. In an open-label, nonrandomised, uncontrolled trial, 15 PD patients were administered desipramine (25–50 mg p.o. tid) with or without trihexyphenidyl. The majority of patients reported some improvement of tremor, rigidity, and bradykinesia following the introduction of desipramine [254].

In an open-label, nonrandomised, uncontrolled trial, desipramine (150 mg p.o. id) was administered to 40 L-DOPA-untreated patients with Parkinsonism (17 patients with idiopathic PD, 20 patients with postencephalitic PD, and 13 patients with atherosclerotic PD). 12 patients reported they were improved by desipramine and 18 patients reported they were somewhat improved, whereas 10 patients did not see any improvement. Mood and tremor were the most improved symptoms [496].

In a nonrandomised, single-blind, uncontrolled study, desipramine (75 mg p.o. id) was administered as monotherapy to 7 PD patients or was combined (50 mg p.o. id) to either procyclidine or trihexyphenidyl in 6 PD patients. On day 50, desipramine, in both groups, had significantly improved akinesia, rigidity, tremor, and vegetative symptoms. Mood was significantly improved only in the combined therapy group [497].

In a case-report study, desipramine (10 mg p.o. id) was effective at alleviating depressive symptoms and L-DOPA-induced oro-facio-lingual dyskinesia in a 61-year-old depressed PD man. However, the drug had to be stopped because, when added to L-DOPA, bromocriptine, and trihexyphenidyl, it triggered agitation and visual hallucinations [313]. In another case-report study, desipramine (300 mg p.o. id) significantly alleviated anxiety in a 39-year-old L-DOPA-treated man with PD for 6 years. Of note, the anxiety had not responded to trimipramine (50 mg p.o. hs) and benzodiazepines [322].

A study measured the urinary levels of monoamine and their metabolites when desipramine (150 mg p.o. id) was combined to L-DOPA and an AADC inhibitor. Desipramine did not modify the urinary excretion of dopamine, 5-HIAA, or 3-methyl-tyrosine. No behavioural data were provided [498].

As mentioned earlier, in a randomised, double-blind, placebo-controlled trial, the antidepressant efficacies of citalopram and desipramine were compared in 48 nondemented depressed PD patients. Both citalopram and desipramine significantly improved depressive symptoms, but desipramine onset of action was shorter than citalopram [146, 147].

An EBM review published by the MDS in 2011 stated that desipramine was “likely efficacious” for the treatment of depression in PD [155].

### 6.5. L-Amphetamine

Detailed discussion of the pharmacology and behavioural effects of L-amphetamine is presented in the “Amphetamine, Methamphetamine, and Propylhexedrine” subsection. Briefly, L-Amphetamine could be effective against tremor and rigidity. The chemical formula of L-amphetamine is provided in Figure 3.

### 6.6. Maprotiline

Maprotiline, a tetracyclic antidepressant, is a selective NET inhibitor with moderate affinity for SERT and DAT (Table 1). Maprotiline also exhibits high affinity at H_1_, *α*_1_, M, and D_2_ receptors, as well as moderate affinity at the *α*_2_ receptors (Kd of 2.0, 90, and 570 nM and 9.4 *μ*M, resp.) [398]. The chemical formula of maprotiline is presented in Figure 3.

In a case-report study, maprotiline (40 mg p.o. id) was ineffective at treating fibromyalgia symptoms in a 65-year-old L-DOPA-treated PD man. No reports were made concerning the effects of maprotiline on Parkinsonism [228].

In a case-report study, a 52-year old man with PD with cognitive decline was administered maprotiline (50 mg i.v. id for 4 days, followed by 50 mg p.o. id) as an add-on to L-DOPA and bromocriptine. The cognition and memory improved shortly after the initiation of maprotiline and the effect was sustained after 6 months. The tests used to evaluate objectively the effects of maprotiline were not mentioned, nor were the mood of the patient or the effects of maprotiline on motor aspects of PD [499]. In another case-report, a 57-year-old man with PD developed paroxysmal hypertension while receiving L-DOPA, selegiline and maprotiline (75 mg p.o. id), theophylline, and ephedrine. The precise contribution of maprotiline on the hypertension is difficult to determine, considering the various drugs he was taking [500].

In a nonrandomised, unblinded, uncontrolled study of 10 PD patients, maprotiline (50 mg i.v. id for 5 days followed by 25 mg p.o. tid for 20 days) improved rigidity in all patients, bradykinesia in 9, and tremor in 7. Some patients were taking concomitant medication, but this was poorly defined [501]. In a nonrandomised, unblinded, uncontrolled study of 10 PD patients, maprotiline (50 mg i.v. id for 5 days followed by 25 mg p.o. tid for 30 days) significantly improved motivation. The drug had a nonsignificant benefit on rigidity and did not modify tremor. Some patients were taking concomitant dopaminergic therapy, but no subanalysis was performed to assess the efficacy of maprotiline as add-on or monotherapy [502].

### 6.7. Mazindol

Mazindol is a selective NET inhibitor that exhibits high affinity for DAT and SERT (Table 1). In a radioligand binding study in which a single dose of mazindol (10 *μ*M) was employed, mazindol displaced 60% of binding at M_2_, 59% of binding at M_1_, 38% of binding at H_1_, and 35% of binding at 5-HT_1A_ receptors. Although no absolute affinity values were provided for these receptors, mazindol clearly binds to them with moderate/low affinity [503]. The chemical formula of mazindol is depicted in Figure 3.

In a mouse study, mazindol (10 mg/kg i.p.) administered 30 minutes before MPTP injections protected against the toxin-induced striatal denervation [46].

[^3^H]-Mazindol binding is reduced in the striatum of PD patients [504, 505], of the MPTP-lesioned nonhuman primate [506, 507] and of the 6-OHDA-lesioned rat [508]. Mazindol has been used as a way to assess the extent of dopaminergic lesion in PD and related animal models in several studies and citing all of the studies where [^3^H]-mazindol was used in such a context is beyond the scope of this paper.

In an open-label study, 6 PD patients were treated with mazindol (1 mg p.o. id) for 70 days and 4 were treated with mazindol (1 mg p.o. id) in combination with bromocriptine (2.5 mg p.o. tid) for 4 weeks. Although no statistical analysis was performed, mazindol was said to improve bradykinesia, rigidity, and tremor [509]. The same authors then performed a 3-week randomised, placebo-controlled trial in which mazindol (1 mg p.o. id) was administered to 6 PD patients and placebo to 4 PD patients. Mazindol significantly improved total Parkinsonism, as well as each of bradykinesia, rigidity, and tremor [509].

### 6.8. Mianserin

Mianserin potently inhibits NET and has moderate/low affinity for SERT and DAT (Table 1). Mianserin also binds to 5-HT_2A_, H_1_, *α*_1_, and M receptors (Kd of 0.4, 1.0, and 54.9 nM and 4.0 *μ*M, resp.) [356]. Mianserin also binds to *α*_2C_, 5-HT_2C_, *α*_2A_, 5-HT_3_, 5-HT_7_, D_1_, D_2_, and D_3_ receptors (Kd of 3.8, 4.4, 4.8, 7.1, and 56 nM, and 1.4, 2.2, and 2.8 *μ*M, resp.) [510, 511]. Because of the high affinity of mianserin to all of these receptors, the primary mechanism of action of the compound is not related to its effect on monoamine transporters. PD studies involving mianserin will nevertheless be summarised in this review. The chemical formula of mianserin is shown in Figure 3.

In the MPTP-lesioned vervet monkey and cynomolgus macaque, local injection of mianserin within the dorsolateral or associative/limbic striatum and the GP pars externa led to a transient hyperactivity followed by exacerbation of parkinsonian disability [512].

Visual hallucinations were reported in a 71-year-old woman with PD taking L-DOPA, lorazepam, and mianserin. However, the effect of mianserin on visual hallucinations was not reported, nor was the effect of mianserin on motor symptoms [513].

In an 8-week, nonrandomised, open-label, add-on trial, mianserin (20–60 mg p.o. id, average 36.7 mg p.o. id) was administered to 12 PD patients with psychotic manifestations on dopaminergic therapy. Mianserin significantly reduced psychotic manifestations, diminished distractibility, and improved recent memory. The UPDRS part III subscore was also significantly improved by mianserin [514].

In an open-label study, mianserin (30 mg p.o. id) was administered to 25 PD patients with psychotic symptoms. All 17 patients with benign hallucinations and 5 out of 8 patients with delusions were improved [515, 516].

In another study involving 13 idiopathic PD patients and one patient with vascular PD, mianserin (10–30 mg p.o. id) improved psychotic manifestations [517]. In a case-report study, mianserin (30 mg p.o. id) reduced the anti-Parkinsonian action of L-DOPA without alleviating psychotic manifestations [516].

### 6.9. Mirtazapine

Mirtazapine (Org 3770, 6-azamianserin) is a tetracyclic antidepressant that exhibits moderate affinity at NET and virtually no affinity at either DAT or SERT (Table 1). Mirtazapine binds to a variety of receptors with stronger potency than it does at NET. Thus, mirtazapine has affinity for H_1_, *α*_2_, *α*_1_, acetylcholine, and D_2_ receptors (Kd of 0.5, 112, 372, and 794 nM and 4.0 *μ*M, resp.) [356]. Mirtazapine also binds to 5-HT_3_, 5-HT_2C_, 5-HT_2A_, 5-HT_7_, D_1_, and D_3_ receptors (Kd of 7.94, 39, 69, and 265 nM and 4.2 and 5.7 *μ*M, resp.) [510, 511]. In addition, mirtazapine displays affinity for 5-HT_2B_, 5-HT_1A_, 5-HT_1B_, and H_2_ receptors (Kd of 199 nM, and 5.0, 12, and 16 *μ*M, resp.) [518, 519]. Mirtazapine is therefore not selective for the monoamine transporters and its primary mechanism of action does not come from a direct interaction with them. Indeed, its interaction with *α*_2_ adrenergic receptors appears to be the most important mechanism by which mirtazapine enhances both serotonergic and noradrenergic neurotransmissions [519]. Nevertheless, a few studies with mirtazapine in PD were performed and are summarised here. The chemical formula of mirtazapine is displayed in Figure 3.

In an open-label add-on study, mirtazapine (30 mg p.o. id) was administered to 20 dyskinetic PD patients for 6 months. Five patients dropped out of the study, one because of visual hallucinations and 2 because of confusion. Mirtazapine significantly reduced dyskinesia severity, assessed by the Abnormal Involuntary Movement Scale (AIMS). The UPDRS part III score was not modified by the addition of mirtazapine [520]. However, mirtazapine did not alleviate dyskinesia in another open-label study [521].

In a case-series of 3 L-DOPA-treated PD patients, mirtazapine (30 mg p.o. id) improved tremor in each of the patients and improved dyskinesia in two of them [522]. In an open-label 30-day study, mirtazapine (30 mg p.o. id) was administered to 25 PD patients and significantly improved (by 7%) the tremor item of the UPDRS part III subscore [523]. Another study reporting the efficacy of mirtazapine for PD tremor is encountered in the literature. However, despite an extensive search, we could not find the study—presented as an abstract in 1999—and therefore could not verify its content. We cite it here to acknowledge the work of the authors. According to cross-referencing, that study was a case-series of 30 PD patients, in which mirtazapine improved tremor [524].

In a case-series, 4 PD patients treated with mirtazapine (15–30 mg p.o. id) for depressive symptoms developed REM-sleep behaviour disorder (RBD). Two of them also developed psychotic features following the introduction of mirtazapine. The RBD and psychotic manifestations resolved upon discontinuation of mirtazapine [525]. In a case-report, mirtazapine (up to 60 mg p.o. id) was administered to a 44-year-old depressed PD woman. She was previously taking L-DOPA, pergolide, selegiline, and memantine. Two days after the dose was increased to 60 mg p.o. id, she tried to commit suicide by self-strangulation, was delusional, and had paranoid ideations [526]. In another case-report, mirtazapine (15 mg p.o. id) alleviated auditory hallucinations in a 41-year-old woman with PD, without any effect on UPDRS part III sub-score [527, 528]. Mirtazapine (30 mg p.o. hs) was also effective at reducing visual hallucinations in a 67-year-old man with PD previously treated without success with clozapine, quetiapine, and rivastigmine [529]. Similarly, mirtazapine (15 mg p.o. id for 1 week and then 30 mg p.o. id) diminished visual hallucinations in an 83-year-old woman with PD whose symptoms had not responded to quetiapine (50 mg p.o. id), risperidone (2 mg p.o. id), and trazodone (50 mg p.o. id). Mirtazapine also improved her mini-mental state examination score, without impairing parkinsonian disability [530].

### 6.10. Nisoxetine

Nisoxetine is a potent NET inhibitor that exhibits high affinity at DAT and moderate affinity at SERT (Table 1). Nisoxetine also has moderate affinity for *α*_1_, *α*_2_, and H_1_ receptors (EC_50_ of 1.6, 6.1, and 17 *μ*M, resp.) [340]. The chemical formula of nisoxetine is depicted in Figure 3.

When administered as monotherapy to the 6-OHDA-lesioned rat, nisoxetine (3, 10, and 30 mg/kg i.p.) did not elicit rotational behaviour. When combined to the DAT inhibitor vanoxerine, nisoxetine did not modulate vanoxerine-induced rotational behaviour (see below) [224].

In the MPTP-lesioned common marmoset not primed to exhibit dyskinesia, nisoxetine (3.0 and 10.0 mg/kg p.o.) significantly reduced motor activity counts, but also significantly, albeit mildly, improved parkinsonian disability (0.3 and 1.0 mg/kg). The combination of nisoxetine and sertraline (both 1.0 mg/kg p.o.) had no effect on motor activity counts but mildly reversed parkinsonian disability. When added to vanoxerine, nisoxetine reduced motor activity without affecting anti-Parkinsonian action (see below) [291].

To our knowledge, no study with nisoxetine was performed in human PD patients.

### 6.11. Nortriptyline

Nortriptyline is a selective NET inhibitor that exhibits high/moderate affinity for SERT and DAT (Table 1). Nortriptyline also displays high affinity for the H_1_, M, 5-HT_2A_, and *α*_1_ receptors (Kd of 6.3, 37, 44, and 55 nM, resp.) [531]. Nortriptyline is the active metabolite of amitriptyline, following demethylation in the liver [531]. The chemical formula of nortriptyline is provided in Figure 3.

In a randomised, double-blind, placebo-controlled, cross-over study, 19 L-DOPA-treated PD patients were administered nortriptyline (25–150 mg p.o. id). Nortriptyline significantly alleviated depressive symptoms and had no effects on Parkinsonism or dyskinesia. However, nortriptyline caused a significant decrease in systolic blood pressure upon standing [532].

In an 8-week randomised, double-blind, placebo-controlled trial, the efficacies of nortriptyline (25–75 mg p.o. id) and paroxetine controlled-release (12.5–37.5 mg p.o. id) were compared in 52 depressed PD patients. At the end of the study, nortriptyline had improved significantly the HDRS score, quality of sleep, and anxiety when compared to placebo, whereas paroxetine had not. Nortriptyline did not significantly reduce the HDRS scores when compared to paroxetine. Nortriptyline and paroxetine had no effect on UPDRS scores [533].

In a case-report, a 47-year-old L-DOPA-untreated PD woman developed a possible 5-HT syndrome two days after selegiline (10 mg p.o. id) was added to nortriptyline (75 mg p.o. id) [534].

An EBM review published by the MDS in 2011 stated that nortriptyline was “likely efficacious” for the treatment of depression in PD [155].

### 6.12. Reboxetine

The potent NET inhibitor reboxetine also displays high affinity at SERT and virtually no affinity at DAT (Table 1). Reboxetine seems to be selective for these two transporters [349]. The chemical formula of reboxetine is shown in Figure 3.

In the normal rat, reboxetine decreases spontaneous firing activity of the LC neurons. The decrease is slightly, but significantly, enhanced following 6-OHDA lesion. In addition, in the normal rat, reboxetine exerts an inhibitory effect on DRN neurons; this inhibitory effect is reduced following 6-OHDA lesion [187].

In a case-report study, reboxetine (4 mg p.o. id) improved depressive symptoms in a 68-year-old PD woman in whom amitriptyline and fluoxetine had previously been tried without success. Reboxetine did not affect motor function [210].

In a 4-week open-label study, the efficacy of reboxetine (4–8 mg p.o. id) was assessed in 16 PD patients. Reboxetine significantly improved the HDRS score. One patient withdrew from the trial because of delusions and visual hallucinations. Two patients experienced transient increase in hand tremor, but the UPDRS part III subscore was not significantly modified. The effect on dyskinesia was not reported. L-DOPA doses were not significantly different between baseline and completion of the study [535].

In a 4-month open-label, rater-blinded study, reboxetine (average dose of 4.2 mg p.o. id) significantly improved HDRS scores in 13 depressed PD patients. UPDRS scores remained unchanged throughout the treatment period [536].

### 6.13. NET Inhibitors: Summary

The following NET-selective MAUIs have been used in studies in PD and/or related animal models: amoxapine, amphetamine (and parent compounds), atomoxetine, desipramine, maprotiline, mazindol, mianserin, mirtazapine, nisoxetine, nortriptyline, and reboxetine. Results of the studies involving NET inhibitors in PD are summarised in Table 5.

In weighing evidence based on quality of data, we conclude selective NET inhibitors as follows:

1. they probably exert an anti-Parkinsonian benefit as monotherapy;
2. they are probably effective at alleviating depression and anxiety and their onset of action might be quicker than and their antidepressant efficacy superior to SSRIs;
3. there are not enough data to draw conclusions relative to the effect of selective NET inhibitors on L-DOPA anti-Parkinsonian action, but NET inhibitors might worsen the severity of L-DOPA-induced dyskinesia.

Indeed, when administered as monotherapy or in combination with a SERT inhibitor, nisoxetine reversed Parkinsonism in the MPTP-lesioned marmoset, suggesting that both selective NET and mixed NET = SERT inhibitors might represent effective anti-Parkinsonian therapies when administered as monotherapy. In clinical settings, this anti-Parkinsonian efficacy has been achieved as well, although the findings tended to be inconsistent across studies. Discussion about potential mechanisms underlying the anti-Parkinsonian action of selective NET and mixed NET/SERT inhibitors as monotherapy is performed in the “DAT = SERT Inhibitors” section (see below). Other interesting effects achieved with selective NET inhibitors in clinical settings include enhancement of cognition and motivation, as well as a wake-promoting effect.

## 7. DAT Inhibitors

### 7.1. Amineptine

Amineptine displays moderate affinity for DAT and low affinity for both NET and SERT (Table 1). In screening assays with a concentration up to 10 *μ*M, amineptine did not bind to 5-HT_1A_, 5-HT_2A_, D_2_, *α*_1_, *α*_2_, H_1_, benzodiazepine, or gamma-aminobutyric acid (GABA) sites [327]. The chemical structure of amineptine is illustrated in Figure 4.

When administered to rats one hour prior to intraventricular 6-OHDA, amineptine (20 mg/kg i.p.) significantly attenuated the loss of striatal dopamine caused by the toxin [537].

In a randomised, double-blind 4-week study, amineptine (200 mg p.o. id) was compared to the reversible MAO-A inhibitor moclobemide (300–450 mg p.o. id) in 40 depressed PD patients. Both compounds significantly improved the HDRS score when compared to baseline. No motor adverse effects were reported [538].

### 7.2. Modafinil and Armodafinil

Modafinil exhibits moderate affinity for DAT, mild affinity for NET, and virtually no affinity for SERT (Table 1). In addition, modafinil modulates GABA and glutamate release in the striatum, GP, and SN [539]. In the cortex, modafinil decreases GABA release and increases levels of glutamine synthetase [540, 541]. Modafinil also interacts with the orexin and histamine systems [357]. Armodafinil is the R-enantiomer of modafinil and, although its specific binding profile has not been disclosed, it seems to exhibit affinities similar to those of its racemate [542]. The chemical formulae of modafinil and armodafinil are depicted in Figure 4.

Several studies were performed in parkinsonian rodents with modafinil. Modafinil was shown to be neuroprotective against striatal ischaemia [543] and MPTP-induced toxicity in mice [544–546]. In a rat study, modafinil was demonstrated to reduce nigral neuronal loss following hemitransection of the ascending dopaminergic system. Striatal dopamine, 5-HT, and noradrenaline losses were also reduced when animals were administered modafinil [547].

In the common marmoset, modafinil (10, 30, and 100 mg/kg p.o. id), administered daily during MPTP treatment and for up to two weeks following its ending, dose-dependently prevented neuronal loss in the SN. In addition, the administration of acute challenges of modafinil (10, 30, and 100 mg/kg p.o.) as monotherapy to MPTP-lesioned common marmosets not primed to exhibit dyskinesia, dose-dependently reversed the parkinsonian phenotype [548]. A* postmortem* study performed in the MPTP-lesioned common marmoset found that modafinil treatment (100 mg/kg p.o. id during MPTP administration and for up to two weeks following its cessation) abolished the increase in GABA_A_ receptor binding in the GP pars interna of parkinsonian marmosets [549].

In the 1-methyl-1,2,3,6-tetrahydropyridine- (MTP-) lesioned common marmoset, modafinil (100 mg/kg p.o. id started the day of the MTP injection and continued for 27 days) significantly, albeit incompletely, reversed the reduction of striatal dopamine, dopamine metabolites, and 5-HT. Behavioural deficits were also less severe in the MTP-modafinil group than in the MTP-vehicle group [550]. Concordant results were obtained in a magnetic resonance imaging with spectroscopy study that used the same MTP administration paradigm. In this study, the *N*-acetylaspartate (NAA)/phosphocreatine ratio was significantly reduced in the SN of MTP-vehicle marmosets. In contrast, in the MTP-modafinil marmosets, 3.5 weeks following the beginning of the MTP treatment, the NAA/phosphocreatine ratio in the SN was significantly increased when compared to baseline, indicating a neuroprotective effect [551]. In a complementary study, the same group assessed the effect of modafinil (100 mg/kg p.o.) as monotherapy in stable MTP-lesioned marmosets and demonstrated that the drug reversed the parkinsonian phenotype [552].

Despite these anti-Parkinsonian effects of modafinil in the MPTP-lesioned primate, studies assessing modafinil in idiopathic PD have focused on the alertness-enhancing properties of the drug. As such, although several of the studies cited in the current section disclose some effects of modafinil on motor parameters, they were not primarily designed to evaluate the effects of modafinil on Parkinsonism and dyskinesia and may thus lack sensitivity to detect subtle but meaningful changes in L-DOPA anti-Parkinsonian efficacy or L-DOPA-induced dyskinesia.

In a case-report, modafinil (400 mg p.o. id) significantly improved the Epworth Sleepiness Scale (ESS) in a 59-year-old PD woman treated with L-DOPA, amantadine, and sertraline. No effects were reported on motor symptoms and dyskinesia [553]. In another case-report, modafinil (200 mg p.o. id) significantly improved the ESS in a 33-year-old PD woman treated with pramipexole and amantadine. The effects on motor symptomatology were not mentioned [554]. In a case-report, modafinil (100 and 200 mg p.o. id) was effective at normalising the ESS in a 65-year old PD woman treated with L-DOPA, amantadine, and selegiline. Again, the effects of modafinil on motor symptoms were not mentioned [555].

In an open-label add-on study, modafinil (mean dose of 172 mg p.o. id) significantly improved ESS in 9 PD patients treated with L-DOPA and/or dopamine agonists. Modafinil had no effect on UPDRS score. One patient dropped out of the study because of modafinil-induced visual hallucinations. Modafinil did not exacerbate dyskinesia severity in the only dyskinetic patient enrolled [556].

In a randomised, placebo-controlled, double-blind, add-on pilot study, modafinil (400 mg p.o. id) slightly improved the ESS score in 49 PD patients. No differences were found using the Fatigue Severity Index (FSI). No effects were noted on motor function [557]. In a randomised, double-blind, placebo-controlled, add-on study, modafinil (400 mg p.o. id) was administered to 13 PD patients with excessive daytime sleepiness (EDS). Modafinil had no effect on the FSI or the Fatigue Severity Scale (FSS) scores, but significantly improved the ESS when compared to baseline, whereas placebo did not. There were no changes in the UPDRS and Hospital Anxiety and Depression Scale (HADS) scores at the end of the study [557, 558]. In a randomised, double-blind, placebo-controlled, cross-over study of 21 PD patients with EDS, modafinil (200 mg p.o. id) significantly improved the ESS score when compared to baseline. However, only the first period of treatment was analysed, because of a carryover effect. Modafinil had no effect on UPDRS part I–III subscores [559]. In a randomised, double-blind, placebo-controlled, cross-over add-on trial, modafinil (200 mg p.o. id) significantly improved ESS score in 13 patients. Modafinil had no effect on sleep latency in the Maintenance of Wakefulness test. Modafinil did not change the BDI score. The effect of modafinil treatment on motor parameters was not reported [560].

However, in a randomised, double-blind, placebo-controlled, add-on trial, modafinil (100 mg p.o. bid) failed to improve fatigue in 19 PD patients. In that study, modafinil improved the tapping frequency at the Alternate Finger Tapping Test, suggesting it might have an effect against physical fatigability, according to the authors. The effects of modafinil on Parkinsonism and dyskinesia were not mentioned [561, 562]. Another randomised, double-blind, placebo-controlled, add-on study failed to show any benefit of modafinil over placebo for EDS. In that study performed in 37 PD patients, modafinil (200 mg p.o. bid) did not significantly improve the EDS, FSS, or HDRS scores, and sleep latency was not improved either. The UPDRS part II and III subscores were not modified by modafinil [563].

In its 2007 practice parameters for the treatment of narcolepsy and other hypersomniae of central origin, the American Academy of Sleep Medicine stated that “modafinil may be effective for treatment of daytime sleepiness due to PD” [564]. Accordingly, the AAN 2010 practice parameters for treatment of nonmotor symptoms of PD state that modafinil is effective at improving patient's perception of wakefulness, but not at objectively improving EDS [565, 566].

An open-label, randomised, cross-over, safety and efficacy trial comparing methylphenidate and modafinil for the treatment of excessive daytime sleepiness in PD was terminated because of difficulties to recruit patients [567]. An open-label efficacy study examining the effect of armodafinil on attention in PD is currently registered online [568].

### 7.3. MRZ-9547

MRZ-9547 is a DAT inhibitor that displays affinity for its target in the low micromolar range [569]. MRZ-9547 (50 and 100 mg/kg i.p.) elicited ipsiversive rotations in the 6-OHDA-lesioned rat and, when administered with L-DOPA, enhanced the contraversive rotations induced by L-DOPA, without exacerbating AIMs [570]. A Phase I study with MRZ-9547 has recently been performed [571].

### 7.4. SEP-228,791 and SEP-226,330

SEP-228,791 is a selective DAT inhibitor that displays high affinity at NET and virtually no affinity at SERT (Table 1). The binding profile of SEP-228,791 at other sites is unknown. SEP-226,330 is a MAUI, but its binding profile is unknown; nevertheless, in this review article, SEP-226,330 is included in DAT Inhibitors section, but its inclusion in this category may have to be revised. The chemical formulae of both SEP-228,791 and SEP 226,330 have not been disclosed yet.

When administered as monotherapy to MPTP-lesioned macaques primed to exhibit dyskinesia, acute challenges of SEP-228,791 (3, 10 mg/kg p.o.) significantly reduced parkinsonian disability, without eliciting dyskinesia. The effect of SEP-228,791 was more pronounced against bradykinesia than other parkinsonian features. When given in combination with L-DOPA, SEP-228,791 did not enhance the anti-Parkinsonian action of L-DOPA and did not worsen the severity of dyskinesia [363, 572].

Unlike SEP-228,791, SEP-226,330 did not exert any anti-Parkinsonian effect when given as monotherapy to MPTP-lesioned macaques primed to exhibit dyskinesia. However, in combination with low dose L-DOPA, SEP-226,330 (10 mg/kg p.o.) significantly enhanced the anti-parkinsonian benefit of L-DOPA, without increasing the severity of peak-dose dyskinesia. The duration of on-time with disabling dyskinesia was, however, significantly extended when SEP-226,330 was added to L-DOPA [572].

### 7.5. Vanoxerine

Vanoxerine (GBR-12,909) is a selective DAT inhibitor that exhibits high affinity for both NET and SERT (Table 1). Vanoxerine also binds to *σ* receptors with high affinity (EC_50_ of 48 nM) [573]. The chemical formula of vanoxerine is illustrated in Figure 4.

In MPTP-lesioned mice, vanoxerine (10 mg/kg i.p.) significantly reduced the duration of both slow-wave and REM sleep and increased the duration of awakening when compared to saline-treated MPTP-lesioned mice. Vanoxerine (2.5 and 10 mg/kg i.p.) produced similar effects in MPTP-unlesioned mice, but the magnitude of the effects was smaller [132].

In the 6-OHDA-lesioned rat, vanoxerine (2.5, 3, 10, 30, and 60 mg/kg i.p.) induced ipsiversive rotations [224, 370]. The addition of fluvoxamine (3 mg/kg i.p.) to vanoxerine (30 mg/kg i.p.) enhanced this ipsiversive rotational behaviour, whereas the addition of nisoxetine (10 mg/kg i.p.) to vanoxerine (30 mg/kg i.p.) did not modify the number of rotations. The ipsiversive rotational behaviour induced by concurrent administration of vanoxerine (30 mg/kg i.p.), fluvoxamine (3 mg/kg i.p.), and nisoxetine (10 mg/kg) did not differ to the one induced by the combination of vanoxerine (30 mg/kg i.p.) and fluvoxamine (3 mg/kg i.p.) [224].

In the MPTP-lesioned common marmoset primed with L-DOPA to exhibit dyskinesia, vanoxerine (10 mg/kg p.o.) as monotherapy reversed parkinsonian disability and increased motor activity counts to levels comparable to those attained with submaximal dose of L-DOPA (12.5 mg/kg p.o.), without eliciting dyskinesia [574].

In the MPTP-lesioned common marmoset not primed to exhibit dyskinesia, vanoxerine (2.5, 5, 10 mg/kg p.o.) significantly increased motor activity counts and reversed Parkinsonian disability. The addition of nisoxetine (1 mg/kg p.o.) to vanoxerine (10 mg/kg p.o.) led to a significant reduction in motor activity counts when compared to vanoxerine alone but did not affect the anti-Parkinsonian benefit. In contrast, the addition of sertraline (1 mg/kg p.o.) to vanoxerine (10 mg/kg p.o.) significantly reduced motor activity counts and vanoxerine anti-Parkinsonian action. The degree of Parkinsonism was however still lower than in vehicle-treated animals. When vanoxerine (10 mg/kg p.o.), nisoxetine (1 mg/kg p.o.), and sertraline (1 mg/kg p.o.) were administered in combination, motor activity counts did not differ to vehicle-treated animals, but there was still a mild reversal of the parkinsonian disability [291].

### 7.6. DAT Inhibitors: Summary

The following DAT-selective MAUIs have been used in studies in PD and/or related animal models: amineptine, modafinil, SEP-228,791, and vanoxerine. Results of the studies involving DAT inhibitors in PD are summarised in Table 6.

In weighing evidence based on quality of data, we conclude that selective DAT inhibitors

1. exert an anti-Parkinsonian benefit when administered as monotherapy; this anti-Parkinsonian benefit is not accompanied by dyskinesia in animals that were primed with L-DOPA;
2. do not enhance L-DOPA anti-Parkinsonian action;
3. could exert wake-enhancing effect.

Indeed, studies performed in two nonhuman primate species, the common marmoset and the cynomolgus macaque, have shown similar results that is monotherapy with selective DAT inhibitors reverse parkinsonism to an extent comparable to L-DOPA. Importantly, this anti-Parkinsonian benefit is not marred by dyskinesia. However, DAT inhibitors do not enhance L-DOPA anti-Parkinsonian efficacy. These findings have important clinical implications, as they suggest that selective DAT inhibitors could be used in PD, perhaps early in the disease, as L-DOPA-sparing agents, a strategy that might be used to delay the onset of dyskinesia. However, the potential use of DAT-selective inhibitors as agents to enhance L-DOPA anti-Parkinsonian benefit, that is, alleviating wearing-off, is not supported by the available preclinical data. Interestingly, combining a DAT inhibitor to a NET or a SERT inhibitor, thereby resulting in mixed dopamine/noradrenaline or dopamine/5-HT reuptake inhibition, also elicits an anti-Parkinsonian benefit when administered in the absence of L-DOPA, suggesting that enhancing the function of the remaining nigrostriatal dopaminergic fibres, whether selectively or unselectively, is sufficient to exert an anti-Parkinsonian action. Thoroughly designed clinical studies are needed to evaluate these promising preclinical findings.

Despite methodological issues and contradictory results, the numerous studies performed with modafinil suggest that the compound may reduce daytime sleepiness. It remains to be seen if this wake-promoting effect can be generalised to the other DAT inhibitors discussed in the current subsection, as the pharmacology of modafinil is unique, since it also modulates glutamatergic and GABAergic transmissions.

## 8. DAT = NET Inhibitors

### 8.1. Benztropine

Benztropine (or benzatropine) is a potent DAT = NET inhibitor that exhibits only weak affinity for SERT (Table 1). The chemical formula of benztropine is presented in Figure 5. In PD, benztropine is primarily used as an anticholinergic agent [575]. Because of that, studies performed with benztropine in PD are not reviewed in the present paper.

### 8.2. Brasofensine

Brasofensine (NS 2214, BMS-204,756) is a potent dual DAT = NET inhibitor that also exhibits high affinity for SERT (Table 1). The pharmacological profile of brasofensine outside of the monoamine transporters is unknown. The chemical formula of brasofensine is depicted in Figure 5.

In the MPTP-lesioned common marmoset primed to exhibit dyskinesia, brasofensine as monotherapy (0.5 mg p.o. id) significantly increased motor activity but did not induce dyskinesia, stereotypy, or hyperactivity. Over the 11 days during which the marmosets were treated, a 10% weight loss occurred [576]. The effect of combining brasofensine to L-DOPA in these marmosets primed to exhibit dyskinesia was not assessed.

In the MPTP-lesioned common marmoset not primed to exhibit dyskinesia, brasofensine (0.5, 1.0, and 2.5 mg/kg p.o.) as monotherapy significantly increased motor activity counts and reduced parkinsonian disability. In combination with low dose L-DOPA (2.5 mg p.o.), low dose brasofensine (0.25 mg p.o.) significantly increased motor activity counts and improved parkinsonian disability when compared to either L-DOPA (2.5 mg p.o.) or brasofensine (0.25 mg p.o.) alone. Combining low dose brasofensine (0.25 mg p.o.) with a higher dose of L-DOPA (12.5 mg p.o.) did not further increase motor activity counts or reduce the parkinsonian disability when compared to L-DOPA (12.5 mg p.o.) alone or to the combination of low dose L-DOPA (2.5 mg p.o.) and low dose brasofensine (0.25 mg p.o.) [577, 578].

In a randomised, double-blind, placebo-controlled, escalating-dose study, brasofensine (0.5, 1.0, 2.0, and 4.0 mg p.o. id) was administered to 8 L-DOPA-treated PD men. Brasofensine did not improve the UDPRS part III subscore, the 10-metre walking test, or the finger tapping test. No effect was reported on the severity of dyskinesia. In a complementary pharmacokinetic study, the maximal plasma concentration was 3.27 ng/mL in patients who were administered 4.0 mg p.o., which corresponds to a plasma concentration slightly inferior to 10 nM, at which brasofensine is likely to behave as a mixed DAT/NET inhibitor. Accordingly, all of the oral doses employed in the study should have given plasma concentrations between 1 and 10 nM, at which brasofensine is also likely to behave as a DAT = NET inhibitor [579–581]. Brasofensine has excellent brain penetration, with brain levels higher than plasma levels for 12 h following its administration [582], thus, lack of brain availability is unlikely to account for the lack of effect of brasofensine on parkinsonism.

In a 4-week, placebo-controlled, Phase II trial, brasofensine was administered to 95 recently diagnosed PD patients. Brasofensine (2, 3 mg p.o. id) significantly improved the UPDRS after one week of treatment, but the improvement was no longer significant after 2 and 4 weeks [583].

The development of brasofensine has been discontinued [584].

### 8.3. Bupropion

Bupropion inhibits DAT and NET with high/moderate affinity and has moderate/low affinity for SERT (Table 1). Bupropion is also a moderate-/low-affinity antagonist at nicotinic acetylcholine receptors (EC_50_ of 1.5 *μ*M at *α*_3_ *β*_4_ subunits and 10 *μ*M at *α*_1_ *β*_1_ *γδ* subunits) [585]. Bupropion also binds with moderate affinity to *α*_1_ and H_1_ receptors (Kd of 4.6 and 6.6 *μ*M, resp.) and with low affinity to *α*_2_ and M receptors (Kd > 45 *μ*M for both) [398]. The chemical formula of bupropion is illustrated in Figure 5. The anti-Parkinsonian efficacy of the metabolites of bupropion, R,R- and S,S-hydroxybupropion was also tested (see below). R,R-Hydroxybupropion does not exhibit any affinity for either DAT, NET, or SERT [361, 362], whereas S,S-hydroxybupropion inhibits both the DAT and NET with high/moderate affinity (Table 1).

In a rat study in which 6-OHDA was administered intracisternally, bupropion (25, 50, and 100 mg/kg i.p. given 30 minutes before 6-OHDA) significantly reduced the loss of striatal dopamine. When bupropion (15 and 25 mg/kg i.p.) was administered as monotherapy to 6-OHDA-lesioned rats with chronic bilateral striatal dopamine depletion, it failed to increase motor activity [586]. A study presented as an abstract suggested that bupropion (10 mg/kg i.p.) improved anhedonic behavioural deficits following 6-OHDA lesion in the rat [270, 271].

In the MPTP-lesioned common marmoset not primed to exhibit dyskinesia, bupropion (6, 12.5, 18, and 25 mg/kg p.o.) as monotherapy had no effect on motor activity counts and parkinsonian disability [291]. In another study in which the priming status of the animals was not mentioned, bupropion (25 mg/kg, route of administration not mentioned) significantly reduced the total parkinsonian disability over a 4 h observation period, in the MPTP-lesioned common marmoset [587]. In another study performed in the MPTP-lesioned common marmoset (priming status unknown), bupropion (25 mg/kg p.o.) had no effect on motor activity counts, but significantly improved parkinsonian disability over a 4 h observation period [588]. Dyskinesia was not evaluated in that last study.

In a case-report, bupropion (150 mg p.o. id) was administered to a 57-year-old PD woman with anxiety, panic disorder with agoraphobia and somatic complaints. Bupropion was added to her preexisting medication (L-DOPA, pramipexole, rotigotine, selegiline, and mirtazapine). Her psychiatric symptoms were improved by bupropion. The effect of bupropion on motor phenomenology was not reported [589]. In another case-report, bupropion (150 mg p.o. bid) improved HDRS in a 70-year-old PD woman with treatment-resistant depression. No changes were noted on the UPDRS part III subscore, but the patient felt that bupropion had improved her bradykinesia and rigidity [590]. Bupropion (150 mg p.o. id) also significantly improved depressive symptoms in a 78-year-old lady with PD who had not responded to previous therapies with fluoxetine (40 mg p.o. id) or mianserin (30 mg p.o. id) [591]. Bupropion (75 mg p.o. qid) was also administered to a 65-year-old woman with PD and led, along with changes in L-DOPA posology, tramadol, and cyclobenzaprine, to a reduction of pain and depressive symptoms. The precise effect of bupropion on symptoms was not mentioned and the woman was previously on paroxetine (dose not specified) [592]. The addition of bupropion (75 mg p.o. bid) to benztropine and amantadine triggered a delirium in a 75-year-old man with PD [593].

In a series of three case-reports, bupropion (150 mg p.o. id) significantly alleviated dopamine agonist-induced compulsive behaviour and depressive symptoms. Bupropion had no effect on Parkinsonism and did not trigger or exacerbate dyskinesia [594].

In a 12-week open-label study, bupropion (300 mg p.o. id) was administered to 15 depressed PD patients. Bupropion significantly improved the HDRS score without altering motor symptoms [595].

In a randomised, double-blind, placebo-controlled, cross-over add-on study followed by an open-label phase, bupropion (450 mg p.o. id) was studied in 20 PD patients. Bupropion significantly improved gait, postural stability, bradykinesia, and parkinsonian disability score (assessed with the New York University Parkinson Disease Scale and the Northwestern University Disability Scale). Five out of 12 depressed patients were improved. Hallucinations and confusion occurred in 3 patients, whereas dyskinesia was exacerbated in one patient [596].

In an open-label study, bupropion (300 mg p.o. id) was administered to 9 PD patients with advanced disease and freezing of gait for 12 weeks. Bupropion did not improve freezing of gait, evaluated with the Gait and Balance Scale (GABS), and had no effect on UPDRS part III subscore [597, 598].

In the MPTP-lesioned common marmoset (priming status unknown), monotherapy with S,S-hydroxybupropion (6, 12.5, and 18 mg/kg p.o.) significantly increased motor activity and parkinsonian disability over a 4 h observation period [588]. The combination of S,S- and R,R-hydroxybupropion (both 12.5 mg/kg p.o.) also increased motor activity counts and reversed parkinsonism over a 4 h observation period, whereas R,R-hydroxybupropion (6, 12.5, and 18 mg/kg p.o.) had no effect on either motor activity or parkinsonian disability. Dyskinesia was not evaluated in the study [588].

### 8.4. Cocaine

Cocaine is a potent and selective DAT = NET inhibitor that also exhibits high affinity for SERT (Table 1). Cocaine inhibits the current generated by 5-HT at 5-HT_3_ receptors with an EC_50_ of 4.2 *μ*M [599]. Cocaine also binds to *σ* receptors (Kd of 6.7–26 *μ*M) [402] and M_2_ receptors (Kd of 2.2–40 *μ*M for (+)-cocaine and (−)-cocaine, resp.) [403]. The chemical formula of cocaine is illustrated in Figure 5.

Cocaine analogues have been used in many imaging studies to assess extent of striatal dopamine denervation [600–602]. A detailed review of each of these imaging studies is beyond the scope of this paper. Cocaine itself has seldom been used to determine the extent of dopamine denervation within the striatum. Studies using [^3^H]-cocaine found decreased binding levels in the striatum of the 6-OHDA-lesioned rat [603] and in the putamen of patients with idiopathic PD [603, 604].

Adolf Hitler, who likely suffered from PD, reportedly used amphetamines and cocaine [605–616]. In a case-series of two PD patients, cocaine inhalation relieved off periods [617]. In a case-report, intravenous and intranasal cocaine intake several times a week were thought to underlie the parkinsonian phenotype developed by a 35-year-old man, although he had previously taken amphetamines [618]. Intraocular instillation of 40 *μ*L of a 5% cocaine solution to PD patients induced a significantly smaller mydriasis than when the same solution was administered to normal subjects [619]. Intraocular cocaine has also been administered to PD patients with a myosis following thalamotomy, with an ensuing pupillary dilation [620]. Intraocular administration of cocaine was also performed in a case-series aimed at elucidating the mechanism by which L-DOPA sometimes induces pupillary dilation; a peripheral mechanism, not direct stimulation of adrenoceptors, seems to be involved [621, 622].

In an immunohistochemical study performed in MPTP-lesioned mice, cocaine (30 mg/kg i.p.) failed to increase striatal levels of phosphorylated cAMP response element-binding (CREB) and c-Fos, whereas cocaine-induced increases in CREB and c-Fos occurred in normal mice. The behavioural correlates of these findings were not reported [623]. In the 6-OHDA-lesioned rat, cocaine (20 mg/kg i.p.) induced rotations ipsilateral to the lesioned side [624]. Exposure to cocaine* in utero* or during adulthood rendered mice more susceptible to MPTP toxicity, suggesting that cocaine intake might predispose to development of PD [625], although two studies conducted in heavy cocaine users did not find evidence of parkinsonism [626, 627]. In the rat with bilateral 6-OHDA lesions, administration of cocaine (10 mg/kg i.p.) increased motivation, assessed by the conditioned place preference test [628].

In a case-series, 3 PD patients on dopamine agonists experiencing impulse-control disorder began smoking crack cocaine. Two patients stated that cocaine improved their motor function [629].

### 8.5. D-Amphetamine

Detailed discussion about the pharmacology and behavioural effects of D-amphetamine is performed in the “Amphetamine, Methamphetamine, and Propylhexedrine” subsection (see above). Briefly, D-amphetamine could improve gait and depressive symptoms in PD. The chemical formula of D-amphetamine is provided in Figure 5.

### 8.6. Methamphetamine

Detailed discussion about the pharmacology and behavioural effects of methamphetamine and its two enantiomers is performed in the “Amphetamine, Methamphetamine, and Propylhexedrine” subsection (*vide supra*). Briefly, in PD, methamphetamine enhances dopamine release by surviving nigrostriatal axons. The chemical formula of methamphetamine is illustrated in Figure 5.

### 8.7. Methylphenidate

Methylphenidate is a potent dual DAT = NET inhibitor that displays virtually no activity at SERT (Table 1). Methylphenidate also binds to 5-HT_1A_ and 5-HT_2B_ receptors (Kd of 5 and 13 *μ*M, resp.) [630]. In screening assays with a single dose of methylphenidate (10 *μ*M), methylphenidate displaced between 55 and 71% of specific binding at each of M_1–5_ receptors, indicating that the compound has at least moderate affinity for these receptors [630]. A review of methylphenidate in PD was published in 2009 [631]. The chemical formula of methylphenidate is depicted in Figure 5.

Radiolabeled methylphenidate is frequently used as a radioligand in PET studies in PD [632–634] and animal models of PD [635, 636]. Methylphenidate is also used to induce dopamine release prior to PET scanning, though with mixed results [637, 638]. The list of studies cited where radiolabeled methylphenidate has been used as PET ligand in PD is not exhaustive.

When coadministered with 6-OHDA, methylphenidate prevents dopaminergic nigral neuronal loss and the emergence of the parkinsonian phenotype in rat [639]. When administered to 6-OHDA-lesioned rats, methylphenidate causes rotations towards the lesioned side [640]. In a study performed in 3 MPTP-lesioned macaques, methylphenidate (0.3 mg/kg intramuscularly [i.m.]) decreased the number of “no response errors” during a delayed response task test, without improving the overall performance to the test [641].

In a case-report, methylphenidate (5 mg p.o. bid) improved apathy and increased motivation and initiative in an 82-year-old depressed PD man. Depressive symptoms had responded to paroxetine (20 mg p.o. id), but not apathy. Sleepiness was also improved. The effects on motor symptoms were not reported [642]. In a nonrandomised, unblinded pilot study, an acute challenge of methylphenidate (20 mg p.o.) was administered to 21 L-DOPA-treated PD patients. Methylphenidate significantly improved attention but had no effect on executive functions, hand-eye coordination, visuospatial orientation, or memory when compared to baseline. Methylphenidate significantly improved gait and mobility. The effects of methylphenidate on the UPDRS part III subscore or on dyskinesia severity were not reported [643]. Methylphenidate (20 mg p.o. bid) completely abolished dopamine uptake in the basal ganglia in an 80-year-old man with PD who underwent a SPECT scan to the dopaminergic transporter; the behavioural correlate was not provided [644].

In a randomised, double-blind, placebo-controlled, cross-over trial, the effects of an acute oral challenge of L-DOPA and methylphenidate (doses not mentioned) were studied in 15 drug-naïve PD patients. Neither of the two drugs produced a significant subjective benefit, assessed by visual analogue scale (VAS). L-DOPA, but not methylphenidate, improved UPDRS part III subscore. The same experiments were repeated in the same patients after 16.7 months of anti-Parkinsonian therapy. Following anti-Parkinsonian medication washout, both drugs improved positive affect and reward responsivity. Only L-DOPA improved UDPRS part III subscore [645].

In a 6-week randomised, double-blind, placebo-controlled add-on study of 36 PD patients, the efficacy of methylphenidate (10 mg p.o. tid) on fatigue was assessed. Methylphenidate significantly improved fatigue and had no effect on the UPDRS part III subscore [646]. In a case-report, methylphenidate (dose not mentioned) also had a beneficial effect on fatigue in an L-DOPA-treated elderly PD patient [647].

In light of some of the aforementioned studies, the AAN 2010 practice parameters for treatment of nonmotor symptoms of PD stated that methylphenidate is possibly effective at treating fatigue in PD patients [565].

In a placebo-controlled case-series study, the subjective effects of methylphenidate (15–30 mg p.o. id) in 12 PD patients were compared to the subjective effects of the drug in 12 age-matched healthy controls. Overall, healthy subjects seemed to be significantly more responsive to methylphenidate than PD subjects. No effects on motor function were reported in PD patients [648].

In a randomised, double-blind, placebo-controlled study, 25 PD patients were administered an acute challenge of pramipexole and methylphenidate (10 mg p.o.). Methylphenidate improved vigour, pleasure, and the motor series Luria task when compared to placebo, whereas pramipexole did not [649].

In a double-blind, placebo-controlled, cross-over trial, methylphenidate (3 injections of 0.4 mg/kg i.v. 10 minutes apart) significantly improved PD-related pain symptoms (scored according to a 0–5 intensity scale) when compared to placebo and baseline, in 8 L-DOPA-treated PD patients. The authors did not mention if an anti-Parkinsonian medication washout was performed before administration of methylphenidate. The effects of methylphenidate on motor symptoms were not discussed [650].

In a randomised, double-blind, placebo-controlled study, methylphenidate (0.4 mg/kg i.v.) was administered to depressed (*N* = 13) and nondepressed (*N* = 11) PD patients to study its effect on mood following a 72-hour withdrawal of anti-Parkinsonian medication and a 2-week withdrawal of antidepressant medications. Methylphenidate produced an improvement in the euphoria state of nondepressed PD patients but had no effect on depressed PD patients. Rigidity and bradykinesia were improved in 50% of patients (statistical significance not mentioned), but there was a trend towards tremor worsening [651].

In a nonrandomised, unblinded study of 8 PD patients, methylphenidate (10 mg p.o.) significantly reduced freezing and improved gait after a 12-hour anti-Parkinsonian medication washout [652–654]. In a 6-month randomised, double-blind, placebo-controlled, cross-over trial, methylphenidate (1 mg/kg p.o. id divided in 3 doses, up to 80 mg p.o. id) was administered to 27 PD patients with moderate gait disturbance. 17 patients completed the trial. Methylphenidate did not improve gait and UPDRS score worsened in the active group [655, 656].

In an unblinded, nonrandomised trial of 4 PD patients, methylphenidate (30–40 mg i.v.) improved rigidity and range of movement. Tremor was worsened in one patient [657]. In a subsequent 16-week randomised, double-blind, placebo-controlled, cross-over trial performed in 12 PD patients, monotherapy with methylphenidate (total dose of 60 mg p.o. id) ameliorated freedom of movement and rigidity and had a beneficial effect on mood [657].

In an article published in Italian, methylphenidate (2.5–35 mg p.o. id) was administered to a few PD patients in combination with reserpine. Methylphenidate was not effective in that study [658]. In a similar study, where methylphenidate (5 mg p.o. tid or qid) was administered to 18 patients with postencephalitic parkinsonism in combination with reserpine and led to an improvement in tremor, mood, and mental functioning [659].

In a randomised, double-blind, placebo-controlled trial, the effects of adding methylphenidate to L-DOPA were assessed. Following an overnight washout of their anti-Parkinsonian medication, 5 PD patients were administered methylphenidate (0.2 mg/kg p.o.) or placebo in combination with L-DOPA (2 mg/kg/h i.v.) or placebo. The methylphenidate/L-DOPA treatment significantly improved right hand tapping when compared to the placebo/L-DOPA treatment. UPDRS part III subscore and dyskinesia were not different between treatments. Methylphenidate did not improve the enhancement of the “choice reaction time” cognitive test noted in the L-DOPA/placebo group [660].

In a randomised, triple-blind, placebo-controlled trial, methylphenidate (0.4 mg/kg p.o.) was administered to 17 PD patients undergoing an i.v. L-DOPA infusion (0.5 or 1.0 mg/kg/h) following an overnight anti-Parkinsonian medication washout. When given to patients in the off-state, methylphenidate had no effect on motor disability, anxiety, mood, or energy. When added to L-DOPA, methylphenidate significantly increased tapping and walking speeds and enhanced mood in comparison to L-DOPA alone. However, the addition of methylphenidate to L-DOPA increased the percentage of subjects exhibiting dyskinesia, without exacerbating dyskinesia duration or severity, when compared to L-DOPA alone. Peak plasma L-DOPA levels were not affected by methylphenidate [661].

In a randomised, double-blind, placebo-controlled, cross-over trial, methylphenidate (0.4 mg/kg p.o. tid) was administered to 13 L-DOPA-treated PD patients with motor fluctuations. Twelve patients completed the trial. Despite a trend, methylphenidate failed to extend on-time duration. Methylphenidate significantly improved tremor and had no significant effect on dyskinesia severity [662].

In a rater-blinded study, methylphenidate (1 mg/kg p.o. tid) was studied in 17 PD patients with STN deep-brain stimulation. As monotherapy, methylphenidate significantly improved gait and UPDRS part III subscore when compared to baseline. In combination with L-DOPA, methylphenidate significantly improved gait and UPDRS part III subscore, without worsening dyskinesia, when compared to L-DOPA alone. In an open-label phase following study completion, methylphenidate significantly reduced sleepiness and improved UPDRS part I and II subscores, as well as selective and sustained attention [663]. In a randomised, placebo-controlled, double-blind, multicentre extension of that study, 69 PD patients with STN deep-brain stimulation were randomised to receive either methylphenidate (1 mg/kg daily) or placebo in combination with moderate dose of L-DOPA. After 12 weeks, there was a significant decrease in the time and number of steps in the Stand Walk Sit test. Freezing and freezing of gait were also reduced, as were UPDRS part III subscore and reaction time [664–666].

As mentioned above, an open-label, randomised, cross-over, safety and efficacy study comparing methylphenidate and modafinil for the treatment of excessive daytime sleepiness in PD was prematurely terminated because of difficulty with patient recruitment [567]. A randomised, double-blind, placebo-controlled, cross-over study evaluating the effect of methylphenidate on nonmotor symptoms and postural control in PD patients was also prematurely terminated because of difficulty with enrolment and no observable benefit after an interim analysis of 6 patients [667].

### 8.8. Nomifensine

Nomifensine (HOE 984) is a dual DAT = NET inhibitor that exhibits high/moderate affinity at SERT (Table 1). Nomifensine also binds to 5-HT_2A_, 5-HT_1A_, and 5-HT_2C_ receptors (Kd of 603, 977 nM and 4.1 *μ*M, resp.) [400], as well as to *α*_1_ and *α*_2_ H_1_ and M receptors (Kd of 0.85, 6.5, 21, and 250 nM, resp.) [398]. One study also suggested that nomifensine exerts a direct dopaminergic agonist effect [668]. The chemical formula of nomifensine is depicted in Figure 5.

Nomifensine was demonstrated to protect against MPTP toxicity in zebrafish embryos [669] and the cynomolgus macaque [670, 671]. Nomifensine (10 mg/kg i.p.) reduced dopaminergic neuronal death after MPTP administration to the Sprague-Dawley rat but did not alter the toxic effect of MPTP on corticotropin-releasing factor- (CRF-) immunoreactive neurons in the paraventricular nucleus of the hypothalamus and in the amygdala [672].

In the 6-OHDA-lesioned rat, nomifensine (0.3 mg/kg s.c.) induces rotations ipsilateral to the lesioned side [673]. Perfusion of a solution containing 5 *μ*mol/l of nomifensine in the denervated striatum of 6-OHDA-lesioned rats led to an increase in basal dopamine release, but absolute dopamine levels remained lower than those of the nondenervated striatum [674]. Perfusion of a solution containing 6.7 *μ*mol/l of nomifensine in the denervated striatum of 6-OHDA-lesioned rats that were grafted with human foetal mesencephalic tissue led to increases in dopamine, though the magnitude was smaller than that from the intact hemisphere [675]. Local injection of nomifensine (400 nl, 800 *μ*M) in the dorsal striatum of adeno-associated virus (AAV)-synuclein-overexpressing parkinsonian rats also led to increased dopamine levels [676]. In the striatum of parkin knockout mice, dopamine release following administration of nomifensine (7 mg/kg s.c.) is decreased compared to wild-type mice [677]. In the striatum of the leucine-rich repeat kinase 2 (dardarin, LRRK2)^R1441G^ bacterial artificial chromosome transgenic mouse, dopamine release is diminished compared to normal mice following local administration of nomifensine (100 *μ*M) [678].

Radiolabeled nomifensine has also been used as a radioligand to determine the extent of striatal dopamine denervation in PD [679–682] and animal models of PD [683]. The list of studies where radiolabeled nomifensine has been used as a ligand in nuclear imaging studies in PD is not exhaustive.

In the MPTP-lesioned common marmoset not primed to exhibit dyskinesia, monotherapy with nomifensine significantly increased motor activity counts (25 mg/kg p.o.) and improved parkinsonian disability (20 and 25 mg/kg p.o.) [291]. In another study, nomifensine (5 and 10 mg/kg i.p.) reversed bradykinesia, in the MPTP-lesioned common marmoset [684].

Administration of nomifensine (200 mg p.o.) to 6 PD subjects led to an increase in growth hormone levels. The clinical correlate was not mentioned and no comparison was made with control individuals [685].

Administration of nomifensine (200 mg p.o., single dose, administered in the morning) to 11 PD patients who had not taken their usual anti-Parkinsonian medication since the night before, did not alter plasma prolactin levels. Nomifensine mildly improved tremor but worsened bradykinesia [686].

In a case-report, a 67-year-old man who had been suffering from PD for 6 years was treated with L-DOPA 1,000 mg id. Increasing the dose did not provide additional benefit and bromocriptine had to be withdrawn because of visual hallucinations. The addition of nomifensine (50 mg p.o. id) improved mobility, mood, and sleep pattern [687].

In a nonrandomised, double-blind, placebo-controlled cross-over study, nomifensine (up to 200 mg p.o. id) was administered to 29 PD patients for 12 weeks, after which nomifensine was replaced by placebo for 6 weeks. Previous anti-Parkinsonian medication was withdrawn. Nomifensine produced a moderate but significant improvement of Parkinsonism; tremor and facial expression were the most improved parameters. When placebo replaced nomifensine, there was a reemergence of Parkinsonism [688].

In a 12-week randomised, double-blind, placebo-controlled, cross-over,* de novo* study, nomifensine (100–200 mg p.o. id) was administered to 21 previously untreated PD patients (18 of whom were included in the analysis). Tremor, rigidity, speech disorder, and rising from the chair were all mildly, but significantly, improved by nomifensine when compared to placebo [689].

In a nonrandomised, single-blind, uncontrolled study, nomifensine (50 mg p.o. tid) was added to the anti-Parkinsonian medication of 8 PD patients. Nomifensine failed to provide additional anti-Parkinsonian benefit. However, some of the enrolled patients were unresponsive to L-DOPA, suggesting that they might have been suffering from a Parkinson-plus syndrome [690].

In a nonrandomised, single-blind, placebo-controlled, add-on study, nomifensine (75–200 mg p.o. id, average 150 mg p.o. id) was studied in 28 idiopathic PD patients and one postencephalitic PD patient. 19 patients were included in the analysis. When nomifensine was substituted for placebo, there was a significant deterioration of Parkinsonism. Nomifensine also improved the finger flexion test. Severity of dyskinesia was increased in 10 patients [691].

### 8.9. DAT = NET Inhibitors: Summary

The following DAT = NET MAUIs have been used in studies in PD and/or related animal models: benztropine, brasofensine, bupropion, cocaine, D-amphetamine, methamphetamine, methylphenidate, and nomifensine. Results of the studies involving DAT = NET inhibitors in PD are summarised in Table 7.

In weighing evidence based on quality of data, we conclude that mixed DAT = NET inhibitors

1. probably exert an anti-Parkinsonian action when administered as monotherapy, but this effect may fade over time;
2. are probably not useful as adjunct therapy to L-DOPA, at therapeutically relevant doses and may exacerbate the severity of L-DOPA-induced dyskinesia;
3. may be effective at alleviating anxiety and depression.

Indeed, mixed DAT = NET inhibitors exerted an anti-Parkinsonian effect as monotherapy in the parkinsonian primate and in idiopathic PD patients. However, combining mixed DAT = NET inhibitors to L-DOPA does not seem to provide extra anti-Parkinsonian benefit and could be deleterious on dyskinesia. A discussion about possible mechanisms underlying that lack of efficacy at enhancing L-DOPA anti-Parkinsonian action is provided in the “DAT = SERT inhibitors” and “DAT = NET = SERT inhibitors” sections. These data suggest that, as for selective DAT inhibitors, the value of mixed DAT = NET inhibitors in PD may be as L-DOPA-sparing agents, possibly early in the disease process, whereas there is at present no rationale to support their use in advanced disease, as adjunct therapy, to alleviate wearing-off. Simultaneous inhibition of DAT and NET enhanced the anti-Parkinsonian action of subtherapeutic, low dose of L-DOPA in the MPTP-lesioned marmoset, a finding that did not translate into efficacy in clinical settings, where therapeutically relevant doses of L-DOPA were administered. This highlights the importance of designing nonhuman primate studies to mimic as closely as possible the clinical reality. Based on the currently available data, in advanced PD, the use of mixed DAT = NET inhibitors might be as antidepressant, anxiolytic, and/or wake-promoting agents.

Importantly, the anti-Parkinsonian action of brasofensine was not maintained over time, suggesting that tachyphylaxis may occur with repeated administration of the molecule. This is potentially a serious concern, as it may end up by limiting the use of MAUIs in PD. Moreover, tachyphylaxis was not detected at the preclinical level in monkey studies, but it was not addressed either. Indeed, nonhuman primate studies usually consist in administering acute challenges of different doses of a drug, in order to establish a dose-response correlation. Upcoming studies assessing MAUIs in the parkinsonian primate will have to address this important issue of tachyphylaxis.

## 9. DAT = SERT Inhibitors

### 9.1. UWA-101, UWA-121, and UWA-122

UWA-101 is the first equipotent SERT and DAT inhibitor to be developed for the treatment of PD (Table 1). UWA-101 does not display affinity for the 5-HT_1A_, 5-HT_2A_, 5-HT_2C_, 5-HT_1B_, or 5-HT_1D_ receptors [367, 692]. Toxicity assays performed in cell lines have established that the compound is devoid of toxicity [692, 693]. UWA-101 had no effect on the rodent test of prepulse inhibition of the startle reflex, indicating that the compound is devoid of hallucinogenic/psychomimetic effects [692, 694]. The chemical formulae of UWA-101 and UWA-121 are presented in Figure 6, whereas the chemical formula of UWA-122 is depicted in Figure 1.

In the reserpine-treated rat, UWA-101 enhanced L-DOPA-induced horizontal activity and rearing behaviour [692, 695]. In the MPTP-lesioned common marmoset primed to exhibit dyskinesia, UWA-101 (1, 3, 10 mg/kg s.c.) in combination with L-DOPA significantly increased motor activity counts when compared to L-DOPA alone [692, 696]. UWA-101 (3, 6, and 10 mg/kg s.c.) in combination with L-DOPA also significantly increased duration of on-time and “good quality on-time.” UWA-101 did not exacerbate severity of peak-dose dyskinesia but had a deleterious effect on the severity of L-DOPA-induced psychosis-like behaviours [367, 692, 694, 697, 698].

UWA-121 is the R-enantiomer of UWA-101 and retains the affinity of its racemate for both SERT and DAT (Table 1). However, unlike UWA-101, UWA-121 is primarily a DAT > SERT inhibitor. Like UWA-101, UWA-121 does not exhibit activity at NET, 5-HT_1A_, 5-HT_2A_, 5-HT_2C_, 5-HT_1B_, and 5-HT_1D_ receptors [368, 699]. In the MPTP-lesioned common marmoset, UWA-121 dose-dependently extended duration of L-DOPA-induced motor activity [699, 700]. In the MPTP-lesioned common marmoset primed to exhibit dyskinesia, adding UWA-121 (10 mg/kg s.c.) to L-DOPA significantly extended duration of on-time and on-time without dyskinesia. UWA-121 did not exacerbate the severity of L-DOPA-induced dyskinesia or psychosis-like behaviours [368, 369, 697, 699].

UWA-122 is the S-enantiomer of UWA-101 and exhibits affinity for SERT, but not for DAT, NET (Table 1) or any of the 5-HT_1A_, 5-HT_2A_, 5-HT_2C_, 5-HT_1B_, or 5-HT_1D_ receptors. In the MPTP-lesioned common marmoset primed to exhibit dyskinesia, UWA-122 (1, 3, 10 mg/kg s.c.) did not alter the anti-Parkinsonian action of L-DOPA, mildly reduced the severity of dyskinesia, and had no effect on psychosis-like behaviours [368, 699].

The anti-Parkinsonian and antidyskinetic effects of UWA-101 and its two enantiomers as monotherapy have not been assessed yet.

### 9.2. DAT = SERT Inhibitors: Summary

The following DAT = SERT MAUIs have been used in studies in PD and/or related animal models: UWA-101 and UWA-121. Results of the studies involving DAT = SERT inhibitors in PD are summarised in Table 8.

In weighing evidence based on quality of data, we conclude that mixed DAT = SERT inhibitors

1. are promising agents to enhance L-DOPA anti-Parkinsonian action.

Indeed, both UWA-101 and its R-enantiomer significantly extended duration of L-DOPA-induced on-time, without exacerbating the severity of L-DOPA-induced dyskinesia, in the parkinsonian primate. However, DAT = SERT inhibitors might have a deleterious effect on psychiatric complications of L-DOPA therapy, whereas DAT > SERT inhibitors do not appear to have such potential adverse effect, suggesting that the DAT/SERT ratio is critical in order to enhance L-DOPA anti-Parkinsonian benefit without exacerbating motor and/or nonmotor complications of dopaminergic therapy. Although the anti-Parkinsonian effect of dual DAT = SERT inhibitors as monotherapy has not been formally assessed, they might exert a mild anti-Parkinsonian action. Indeed, as seen previously, simultaneous administration of the selective SERT inhibitor sertraline and the selective DAT inhibitor vanoxerine resulted in a weak, albeit significant, reversal of Parkinsonism. Of course, these findings are from preclinical studies performed in the monkey and require confirmation at the clinical level, but dual DAT = SERT inhibitors nevertheless appear promising agents for the treatment of wearing-off.

Mixed DAT/SERT inhibitors are the first class of MAUIs examined so far that enhances L-DOPA anti-Parkinsonian efficacy, suggesting that antagonising simultaneously DAT and SERT is necessary to achieve this benefit. Indeed, antagonising either DAT or SERT alone is no sufficient to enhance L-DOPA anti-Parkinsonian action, and blocking NET on top of either DAT or SERT does not help. Why might this be?

A potential explanation lies in the intimate relation between the serotonergic and dopaminergic systems within the striatum. Thus, serotonergic raphe-striatal fibres contain the AADC [701], can metabolise exogenous L-DOPA into dopamine [73–76, 702–704] and release dopamine [705]. Following dopamine release, 5-HT terminals also participate in its reuptake, via a SERT-mediated mechanism [81, 82]. Although the nonhuman primate model of PD is characterised by severe nigrostriatal degeneration, there are a few intact dopaminergic terminals in the striatum [706, 707]. Therefore, in the absence of DAT inhibition, for instance with selective SERT or mixed SERT/NET inhibition, the remaining dopaminergic fibres may participate in the reuptake of L-DOPA-derived dopamine, thereby preventing enhancement of L-DOPA anti-Parkinsonian action. In the absence of SERT inhibition, that is, in the case of selective DAT or mixed DAT/NET inhibition, raphe-striatal serotonergic fibres may play a similar role, again preventing additional anti-Parkinsonian benefit. Therefore, concomitant inhibition of DAT and SERT appears to be an effective combination to enhance, without exacerbating dyskinesia, L-DOPA anti-Parkinsonian efficacy.

## 10. DAT = NET = SERT Inhibitors

### 10.1. BTS 74,398

BTS 74,398 is a potent triple MAUI (Table 1). Very little is known about its pharmacology outside of the monoamine transporters. The chemical formula of BTS 74,398 is shown in Figure 7.

In the 6-OHDA-lesioned rat, after 21 days of priming with BTS-74,398 (4.7 mg/kg i.p. id), acute challenges of BTS-74,398 (1, 3, 5, and 10 mg/kg i.p.) dose-dependently induced rotations ipsilateral to the lesion that were not accompanied by AIMs. Accordingly, chronic BTS 74,398 treatment did not increase striatal ΔFosB phosphorylation [708], a molecular change associated with L-DOPA treatment and AIMs [709, 710]. Another study using the same dosing regimen found that chronic BTS 74,398 treatment did not change striatal levels of preproenkephalin (PPE)-A, PPE-B or preprotachykinin mRNA [711]. Pretreatment with dopamine antagonists reduced the number of BTS 74,398-induced ipsilateral rotations, whereas pretreatment with 5-HT and *α*_2_-adrenoceptor antagonists increased their number [224].

In the 6-OHDA-lesioned rat, simultaneous administration of BTS 74,398 (4.7 mg/kg i.p.) and L-DOPA (7.4 mg/kg i.p.), led to ipsilateral rotations (BTS 74,398-induced) at the beginning and the end of the behavioural observation period, whereas L-DOPA-induced contralateral rotations were present for the rest of the time. When higher doses of L-DOPA (12.3 and 20.3 mg/kg i.p.) were administered with BTS-74,398 (4.7 mg/kg i.p.), BTS-74,398-induced ipsilateral rotations disappeared and the rotational behaviour observed was exclusively contralateral. BTS 74,398 (3.2 and 6.7 mg/kg i.p.) did not enhance L-DOPA-induced rotational behaviour. Although AIMs were not formally assessed in the study, monotherapy with BTS-74,398 did not elicit AIMs and BTS-74,398 did not seem to exacerbate L-DOPA-induced AIMs severity [712].

In the MPTP-lesioned common marmoset not primed to exhibit dyskinesia, BTS 74,398 (5, 10, or 20 mg/kg p.o.) significantly increased motor activity counts and reversed Parkinsonism when administered as monotherapy [291, 587]. In the MPTP-lesioned common marmoset primed with L-DOPA to exhibit dyskinesia, BTS 74,398 (2.5, 5, 10, or 20 mg/kg p.o.) as monotherapy increased motor activity counts and improved parkinsonian disability, without eliciting dyskinesia. The addition of a low dose of L-DOPA (2.5 mg/kg p.o.) did not lead to further improvement of Parkinsonism or to induction of dyskinesia. When BTS 74,398 (5 mg/kg p.o.) was combined to a submaximal dose of L-DOPA (12.5 mg/kg p.o.), it did not provide additional anti-Parkinsonian benefit compared to the same dose of L-DOPA as monotherapy. The severity of dyskinesia was also unchanged by the addition of BTS 74,398 to L-DOPA [713, 714].

### 10.2. MDMA, R-MDMA, and S-MDMA

Racemic MDMA and its S-enantiomer are nonselective, triple MAUIs, whereas R-MDMA mainly binds to SERT (Table 1). After binding to the monoamine transporters, MDMA blocks monoamine reuptake and reverses transporter gradient, thereby enhancing monoamine release [347, 350, 715]. MDMA also binds to several neurotransmitter receptors. Thus, MDMA exhibits moderate/weak affinity for *α*_2_, 5-HT_2A_, H_1_, M_1_, M_2_, *α*_1_, *β*, D_2_, and D_1_ receptors (Kd of 3.6, 5.1, 5.7, 5.8, 15, 18, 19, 95, and 148 *μ*M, resp.) [352]. MDMA and its metabolite 3,4-methylenedioxyamphetamine (MDA) both exhibit affinity for 5-HT_2B_ receptors (Kd of 500 and 100 nM, resp.) [716]. MDMA is also claimed to bind to 5-HT_1A_ receptors; however, studies demonstrating this used [^3^H]-5-HT as the radioligand and 5-HT as the nonspecific displacer, making the assays nonspecific for 5-HT_1A_ receptors [352, 717]. MDMA is also a weak MAO-A and B inhibitor (EC_50_ of 44 and 370 *μ*M, resp.) [718]. R-MDMA binds to 5-HT_2A_ receptors, at which it acts as a partial agonist [719, 720], as well as to D_2_ receptors (Kd of 3.3 and 25 *μ*M, resp.) [717]. The chemical formulae of MDMA and S-MDMA are presented in Figure 7, whereas R-MDMA is depicted in Figure 1.

Several studies have demonstrated that MDMA is toxic to serotonergic neurons [173, 721–723]. In mice, MDMA potentiates microglial and astroglial activations in the striatum and SN pars compacta following MPTP administration [724]. It was claimed, in one case-report, that MDMA ingestion could cause PD [725]. However, this report was questioned by some scientists [726, 727], because MDMA has not been demonstrated to be toxic to dopaminergic neurons, the unique study claiming such toxicity now being retracted [728, 729]. The patient himself expressed concerns about some of the statements made in the case-report article [727]. In another case-report, juvenile PD developed in a 19-year-old man with a positive family history of PD. PD appeared two months after his last MDMA exposure, which had happened fortnightly for 6 months [730]. Parkinsonism was also reported in a 38-year old man without family history who had used MDMA, cocaine, and lysergic acid diethylamide [731]. A [^18^F]-DOPA PET scan performed in ex-MDMA users showed reduced uptake, up to 3 years after last intake, suggesting that MDMA use may impair nigrostriatal function [732]. Evidence for a potential role of MDMA in the aetiology of PD currently appears tenuous and further studies are needed before concluding that MDMA exposure indeed predisposes to the emergence of PD [733, 734].

At odds with the studies cited in the previous paragraph, there was an anecdotal case-report presented by the BBC about a PD patient who benefited from MDMA intake, MDMA alleviating the severity of L-DOPA-induced dyskinesia whilst prolonging duration of L-DOPA anti-Parkinsonian action [735].

Following this report, several studies were undertaken in animal models of PD. In the rat, racemic MDMA (1, 2.5, and 5 mg/kg s.c.) and each of its enantiomers (2.5 mg/kg s.c.) effectively counteracted haloperidol-induced catalepsy [736–738]. In the 6-OHDA-lesioned rat, racemic MDMA (2.5 and 5 mg/kg s.c.) and its S-enantiomer (5 mg/kg s.c.) elicited rotations ipsilateral to the lesioned side, whereas R-MDMA (5 mg/kg s.c.) did not trigger rotational behaviour [738–740]. Interestingly, administration of citalopram (10 mg/kg s.c.) resulted in a reduction of racemic MDMA-induced rotations [740]. In the 6-OHDA-lesioned rat, racemic MDMA (2.5 mg/kg i.p., but not 0.25 mg/kg i.p.) significantly alleviated L-DOPA-induced AIMs severity but had no effect on L-DOPA-induced rotations [175]. In another study, racemic MDMA (10 mg/kg s.c.) significantly alleviated L-DOPA-induced AIMs and established that the antidyskinetic efficacy of racemic MDMA was not related to striatal dopamine levels, as dopamine levels in the striatum were higher in animals treated with MDMA and L-DOPA than in animals treated with L-DOPA alone [741]. Heterozygous and homozygous parkin knockout mice are more likely to develop MDMA-induced hyperthermia (30 mg/kg i.p.) than wild-type parkin mice [742].

In the MPTP-lesioned common marmoset, when given as monotherapy, MDMA (3, 6, 12 mg/kg p.o.) transiently alleviated Parkinsonism. When administered in combination with L-DOPA or pramipexole, MDMA (3, 12 mg/kg p.o.) significantly decreased the severity of dyskinesia, without altering the anti-Parkinsonian benefit [743]. In the MPTP-lesioned common marmoset, R-MDMA (3, 10 mg/kg s.c.) alleviated L-DOPA-induced dyskinesia, without impairing L-DOPA anti-Parkinsonian efficacy, whereas S-MDMA extended duration of L-DOPA anti-Parkinsonian action, but exacerbated dyskinesia severity [353, 697, 720, 744], thereby establishing that the antidyskinetic effect of racemic MDMA is mediated by its R-enantiomer, whereas the anti-Parkinsonian effect of racemic MDMA is mediated by its S-enantiomer. R-MDMA (3, 10 mg/kg s.c.) also had a beneficial effect on L-DOPA-induced psychosis-like behaviours [697, 720, 744].

In the MPTP-lesioned macaque, administration of MDMA (dose not mentioned) to primed animals exhibiting dyskinesia led to a reduction of dyskinesia after the end of MDMA treatment, even in the absence of MDMA administration. MDMA-induced lesion of the 5-HT system might be the underlying mechanism of this sustained reduction of dyskinesia [745].

### 10.3. Nefazodone

Nefazodone is a triple MAUI (Table 1). However, as for mianserin, mirtazapine, and trazodone, nefazodone exhibits higher affinity at several receptors, which are likely to mediate several, if not the majority, of its biological effects [746]. Thus, nefazodone binds to *α*_1_, 5-HT_2A_, H_1_, 5-HT_1A_, and *α*_2_ receptors with high affinity (Kd of 5.5, 7.1, 30, 52, and 84 nM, resp.) and binds with moderate affinity to M receptors (Kd of 4.6 *μ*M) [288]. The chemical formula of nefazodone is depicted in Figure 7.

In a randomised, single-blind, 3-month study, nefazodone (200–500 mg p.o. id) was compared to fluoxetine (20–50 mg p.o. id) in 16 depressed PD patients. Over the course of the study, UPDRS parts II and III significantly improved in the nefazodone-treated group, whereas they remained unchanged in the fluoxetine group. Both treatments significantly improved the BDI and Clinical Global Impressions (CGI) scales. The effect of nefazodone on dyskinesia was not mentioned [747]. In a case-report, a 70-year-old depressed PD man in whom fluoxetine (20 mg p.o. id) was switched to nefazodone because of worsening of motor symptoms experienced deterioration of parkinsonism following the introduction of nefazodone (50 mg p.o. hs) [748].

An EBM review published by the MDS in 2011 stated that there was “insufficient evidence” regarding the efficacy of nefazodone for the treatment of depression in PD to make any recommendation [155].

### 10.4. S-MDMA

Detailed discussion about the pharmacology and behavioural effects of MDMA and its two enantiomers (R- and S-MDMA) is performed in the “MDMA, R-MDMA, and S-MDMA” subsection (see above). Briefly, S-MDMA extended the duration of L-DOPA anti-Parkinsonian action but had a deleterious effect on dyskinesia, in the MPTP-lesioned common marmoset. The chemical formula of S-MDMA is presented in Figure 7.

### 10.5. Tesofensine

Tesofensine (NS 2330) is a nonselective triple MAUI (Table 1). Tesofensine also stimulates cholinergic neurons of the prefrontal cortex and hippocampus [749]. The chemical formula of tesofensine is depicted in Figure 7.

In a 14-week randomised, double-blind, placebo-controlled proof of concept Phase II trial, tesofensine (0.25, 0.5, 1 mg p.o. id) was administered as monotherapy to 261 patients with PD for less than 5 years. Tesofensine (1 mg p.o. id) significantly improved the UPDRS part III subscore at 6 weeks, but the effect was not sustained. Adverse events were reported more frequently in the tesofensine than in the placebo group, but statistical significance was not provided [750].

In a 4-week randomised, double-blind, placebo-controlled pilot study, tesofensine (1.5 mg p.o. three times a week, preceded by a 1-week placebo treatment course, total tesofensine dose of 12 mg) was administered to 9 patients with advanced PD. Seven patients were in the tesofensine arm and two received placebo. After an overnight anti-Parkinsonian medication washout, patients were administered tesofensine and underwent clinical evaluation (UPDRS part III). Two additional evaluations were performed, that is, during and at the end of an i.v. L-DOPA infusion. Tesofensine did not improve UPDRS part III subscore when compared to baseline. Tesofensine did not increase the anti-Parkinsonian benefit provided by L-DOPA alone and did not worsen dyskinesia severity [751].

In a 14-week randomised, double-blind, placebo-controlled, parallel-group, pilot Phase II trial, tesofensine (0.125, 0.25, and 0.5, 1 mg p.o. id) was administered to 254 advanced PD patients with motor fluctuations. 184 patients completed the trial. No dose-response effect could be demonstrated. Tesofensine (0.25 mg p.o. id) significantly decreased total daily off-time when compared to placebo. A significantly greater proportion of tesofensine-treated than placebo-treated patients had a greater than 20% improvement in their UPDRS part II and III subscores. There was no change in duration of on-time without troublesome dyskinesia, but on-time with troublesome dyskinesia was significantly increased in tesofensine-treated patients (0.25 and 1 mg p.o. id). Although no statistical analysis was performed for the occurrence of adverse events, dyskinesia and insomnia tended to occur more frequently in the tesofensine-treated patients, whereas the prevalence of hallucinations was comparable in the tesofensine- and placebo-treated groups [752].

A meta-analysis incorporating two studies performed on Alzheimer's disease patients and the two 14-week studies cited above shown that tesofensine (0.25, 0.5, 1 mg p.o. id) causes significantly greater weight loss than placebo in both Alzheimer's disease and PD patients [753].

### 10.6. DAT = NET = SERT Inhibitors: Summary

The following DAT = NET = SERT MAUIs have been used in studies in PD and/or related animal models: BTS 74,398, MDMA, nefazodone, S-MDMA, and tesofensine. Results of the studies involving DAT = NET = SERT inhibitors in PD are summarised in Table 9.

In weighing evidence based on quality of data, we conclude that triple DAT = NET = SERT inhibitors

1. exert an anti-Parkinsonian effect when administered as monotherapy but this benefit may not be maintained with chronic administration;
2. probably enhance L-DOPA anti-Parkinsonian efficacy, but this adjunct effect may be compromised by an exacerbation of dyskinesia;
3. may exert a beneficial effect against depression.

As dual DAT = SERT inhibitors, triple DAT = NET = SERT inhibitors seem to enhance L-DOPA anti-Parkinsonian benefit. These data confirm that both DAT and SERT inhibition are needed in order to potentiate L-DOPA anti-Parkinsonian effect. However, unlike dual DAT/SERT inhibitors, the adjunct efficacy of DAT = NET = SERT inhibitors is marred by an exacerbation of dyskinesia.

A potential explanation may be that, as for DAT and SERT, NET participates in dopamine reuptake [86]. However, NET levels are very low within the striatum [754] and, in the parkinsonian state, inhibiting NET, either selectively or in combination with DAT, may not be enough to compensate for DAT/SERT- or SERT-mediated dopamine reuptake. It is therefore possible that simultaneous inhibition of DAT and NET is ineffective at enhancing L-DOPA anti-Parkinsonian efficacy because, in the absence of SERT blockade, synaptic dopamine is uptaken by SERT. However, in the context of dual DAT and SERT inhibition, further blockade of NET may result in too high striatal dopamine levels, the ultimate dopamine-buffering mechanism being neutralised, which might trigger or exacerbate dyskinesia. This is a possible explanation whereby triple DAT = NET = SERT, but not dual DAT/SERT inhibition might exacerbate dyskinesia severity.

Importantly, in clinical settings, the anti-Parkinsonian efficacy of tesofensine as monotherapy was not sustained after chronic administration. As seen above, tachyphylaxis was also encountered when the dual DAT = NET inhibitor brasofensine was administered as monotherapy. Therefore, tachyphylaxis was encountered with two different classes of MAUIs, dual DAT = NET and triple DAT = NET = SERT inhibitors. Would tachyphylaxis also compromise the anti-Parkinsonian benefit of other classes of MAUIs such as dual DAT = SERT inhibitors? Would tachyphylaxis also occur when MAUIs are administered in combination with L-DOPA or would it be limited to scenarios in which MAUIs are administered as monotherapy? These issues need to be addressed, as the occurrence of tachyphylaxis might well seal the fate of MAUIs in PD. However, MAUIs are currently used in clinical settings, both in PD patients and subjects without PD, for instance as antidepressants or wake-enhancing agents, contexts in which tachyphylaxis does not occur. It therefore remains to be established if tachyphylaxis, should it occur, in PD, with every class of MAUIs, would specifically affect the motor aspects of the disease. If such a pessimistic scenario was proven to be true, MAUIs would be practically useless as L-DOPA-sparing agents in early PD or as adjuncts to L-DOPA in advanced PD, but not necessarily as therapies to address nonmotor manifestations of the disease.

## 11. SERT Enhancer

### 11.1. Tianeptine

The mechanism of action of tianeptine is unique when compared to the other compounds discussed in this review article. The affinity of tianeptine for the three monoamine transporters is low (Table 1). Tianeptine does not inhibit but rather enhances 5-HT reuptake* in vivo* [366, 755, 756]. Additionally, tianeptine increases dopamine levels in the rat frontal cortex and nucleus accumbens [757], modulates activity at *α*-amino-3-hydroxy-5-methyl-4-isoxazolepropionic acid (AMPA), and *N*-methyl-D-aspartate (NMDA) receptors [758–762], and could increase BDNF levels [763]. The chemical formula of tianeptine is illustrated in Figure 8.

In an open-label, nonrandomised, uncontrolled, add-on study, tianeptine (12.5 mg p.o. tid) was administered to 18 depressed PD patients. In one and 3 months after the beginning of therapy, the BDI and the HDRS were significantly improved when compared to baseline. The author reported a trend towards an improvement of the UPDRS part III subscore, but data were not provided [764, 765].

### 11.2. SERT Enhancer: Summary

Tianeptine is the only compound with this specific binding profile that was studied in PD. Results of the study involving tianeptine in PD are summarised in Table 10. According to that unique study, tianeptine appears effective against depressive symptoms in PD, but further studies are needed to confirm that finding.

## 12. Concluding Remarks

The first studies with MAUIs in PD were performed in the 1930s. More than 80 years later, and despite numerous preclinical and clinical studies, their use in PD remains minimal and essentially focused on depression. However, as discussed above, the potential uses of MAUIs in PD extend way beyond depression and anxiety and, based on the data collected in this review, it may be possible to tailor the use of MAUIs with a specific profile for a particular manifestation of PD. After presenting more than 700 articles and abstracts discussing more than 50 MAUIs in PD and animal models of PD, we propose the following.

- Selective DAT, mixed DAT = NET, and triple DAT = NET = SERT inhibitors may be used as monotherapy early in PD, as L-DOPA-sparing agents, in order to delay the emergence of dyskinesia. However, tachyphylaxis was encountered when agents with this particular pharmacological profile were used as monotherapy, somewhat dampening the enthusiasm about potential clinical use of these agents. At present, there are no data to justify the use of selective DAT or dual DAT = NET inhibitors as adjunct therapy to L-DOPA and, if triple DAT = NET = SERT inhibitors may effectively extend duration of L-DOPA anti-Parkinsonian effect, the additional on-time appears to be associated with an exacerbation of dyskinesia, somewhat off-setting the favourable effect on on-time duration. An additional benefit of mixed DAT = NET inhibitors is that they may relieve apathy and depression.
- Mixed DAT = SERT inhibitors appear as promising agents to enhance L-DOPA anti-parkinsonian action and could possibly exert a mild anti-Parkinsonian action when administered as monotherapy. However, the experience with these agents is limited to preclinical studies performed in the parkinsonian nonhuman primate and their efficacy needs to be demonstrated in clinical settings.
- Selective SERT, selective NET, and mixed SERT = NET inhibitors appear to exert a favourable effect on nonmotor aspects of the disease, such as anxiety and depression. The antidepressant effect of selective NET and mixed SERT = NET inhibitors may start more rapidly than the antidepressant effect of selective SERT inhibitors. Selective NET inhibitors may also exert favourable effects on cognition, attention, motivation, and daytime sleepiness. Although exacerbation of Parkinsonism has been reported with the use of selective SERT inhibitors, it is relatively infrequent and can be alleviated by increasing dopaminergic medication. SERT-selective inhibitors could also alleviate L-DOPA-induced dyskinesia, but more studies are needed to confirm this potential antidyskinetic benefit.

MAUIs therefore appear as promising molecules in the treatment of PD, as they can potentially address motor and nonmotor manifestations of the disease as well as motor and nonmotor treatment-related complications. However, several issues have been raised in the current review, such as the possibility of tachyphylaxis upon chronic administration. It is also important to bear in mind that, despite their obvious theoretical potential, no MAUI has demonstrated clear effectiveness in the context of Phase III clinical trials in PD, except for a few studies performed in depression. However, it is our hope that reviews such as this one, by summarizing the state of knowledge, generating hypotheses, and addressing unresolved issues, will help in designing better preclinical and clinical studies that will hopefully lead to effective therapeutics for PD.

## Figures and Tables

**Table 1:** Affinity of the monoamine reuptake inhibitors studied in idiopathic PD and animal models of PD.

	DAT	NET	SERT	References
Amineptine	1,400 (EC_50_)–1,800 (EC_50_)	10,000 (EC_50_)	>10,000 (EC_50_)	[327, 328]
Amitriptyline	2,300–8,600	8.6–139	2.8–84	[288, 329–337]
amoxapine	1,900–4,310	4.4–16	58–470	[332, 334]
Amphetamine	560–640	70–120	2,382–3,846	[338]
Armodafinil	n/a	n/a	n/a	n/a
atomoxetine	1,080–1,600	0.7–5	8.9–750	[331, 334, 339, 340]
Benztropine	72 (Kd), 213 (EC_50_)	150 (EC_50_)–667 (EC_50_)	13,000 (EC_50_)	[332, 336, 341]
Brasofensine (NS 2214)	0.79 (EC_50_)	3.13 (EC_50_)	18.0 (EC_50_)	[342]
BTS 74,398	4.2	6.9	19	[343]
Bupropion	520 (Kd), 2,500 (EC_50_)	940 (EC_50_), 52,000 (Kd)	9,100 (Kd), 19,000 (EC_50_)	[331, 332, 334, 336, 339, 341, 344]
Citalopram	20,485–>100,000	4,000–30,285	0.65–19	[128, 288, 329, 332–337, 345]
Clomipramine	1,800–6,200	21 (EC_50_)–13,500	0.05–40	[288, 329, 330, 332–336, 346]
Cocaine	3.03 (EC_50_)–690 (EC_50_)	0.60 (EC_50_), 1,420 (Kd)	180–740	[171, 332, 334, 336, 338, 344, 347, 348]
Desipramine	3,190–11,000	0.31–8.3	17.6 (Kd), 585 (EC_50_)	[288, 329–336, 339, 347, 349, 350]
D-Amphetamine	34 (Kd), 9,600 (EC_50_)	38.9–530	1,840–>100,000	[330, 332, 334, 344, 347]
Dimepramine	n/a	n/a	n/a	n/a
Duloxetine	230–439	1.17–20	0.07–4.6	[165, 337, 351]
Escitalopram	27,410–>100,000	6,514–7,841	1.1–2.5	[128, 345]
Fenfluramine	>10,000	1,987	269	[171, 347]
Fluoxetine	1,600–15,000	143–10,000	0.81–52	[128, 288, 329, 331–336, 339, 345–347, 349, 350]
Fluvoxamine	5,000 (Kd), 42,000 (EC_50_)	500–4,743	1.5–14	[128, 288, 329, 332–335, 345]
Imipramine	5,110–18,000	11–24,000	1.3–200	[288, 329–336, 339, 346, 349]
L-Amphetamine	380 (Kd), 2,900 (EC_50_)	0.14 (EC_50_), 90 (Kd)	10,000	[330, 332, 344]
Maprotiline	2,900 (Kd), 99,000 (EC_50_)	7.4–11.1	3,000 (EC_50_), 5,800 (Kd)	[332, 334–336]
Mazindol	6.5 (EC_50_), 93 (EC_50_)	0.45 (Kd), 8 (EC_50_)	30 (EC_50_), 272 (Kd)	[288, 330, 334, 336, 344, 347, 350]
MDMA	1,572–15,800	462 (Kd), 27,700 (EC_50_)	238 (Kd), 15,900 (EC_50_)	[338, 347, 350, 352, 353]
(−)-methamphetamine	114 (EC_50_)	234 (EC_50_)	2,137 (EC_50_)	[347]
(+)-methamphetamine	4,840 (EC_50_)	48 (EC_50_)	14,000 (EC_50_)	[347]
Methamphetamine	114–470	48–190	2,137–31,740	[338, 354]
Methylphenidate	24 (Kd), 500 (EC_50_)	26.5 (EC_50_), 339 (Kd)	>10,000–132,430	[332, 334, 338, 339, 341, 344]
Mianserin	9,400 (Kd)–40,000 (EC_50_)	22 (EC_50_)–410 (EC_50_)	1,100 (EC_50_)–4,000 (Kd)	[330, 332, 334–336]
Milnacipran	>100,000	22 (Kd)–100 (EC_50_)	8.44 (Kd)–203 (EC_50_)	[337, 355]
Mirtazapine (Org 3770)	>10,000–>100,000	2,511–4,600	>10,000–>100,000	[334, 356]
Modafinil	3,190 (EC_50_)–6,390 (EC_50_)	35,600 (EC_50_)	>500,000 (EC_50_)	[341, 357, 358]
Nefazodone	360–2,380	360–713	137–549	[288, 331, 334]
Nisoxetine (LY-94,939)	200–360	1–180	1,000	[340, 359, 360]
Nomifensine (HOE 984)	0.36 (EC_50_)–269 (EC_50_)	0.11–29	830 (EC_50_), 4,872 (Kd)	[332, 334, 336, 339, 344, 348]
Nortriptyline	1,140–5,000	0.99–820	15–3,600	[288, 329–337, 346]
Paroxetine	268 (Kd), 5,900 (EC_50_)	33–328	0.05–0.73	[128, 288, 329, 331, 333–336, 345]
Propylhexedrine	n/a	n/a	n/a	
Reboxetine	>10,000	1.1–11	129 (Kd), 1,070 (EC_50_)	[339, 349]
R-MDMA	19,300 (EC_50_), >50,000 (Kd)	>20,000 (EC_50_), >50,000 (Kd)	4,740 (EC_50_), 24,500 (Kd)	[350, 353]
R,R-Hydroxybupropion	>10,000	>10,000	n/a	[361, 362]
SEP-226,330	n/a	n/a	n/a	n/a
SEP-228,791	13.5	83	>10,000	[363]
Sertraline	22–315	160 (EC_50_), 1,716 (Kd)	0.047–3.4	[128, 288, 329, 331, 333–335, 345]
S-MDMA	394 (EC_50_), 3,300 (Kd)	136 (EC_50_), 10,930 (Kd)	210 (EC_50_), 514 (Kd)	[350, 353]
S,S-Hydroxybupropion	790 (EC_50_), 1,295 (Kd)	520 (EC_50_), 3,870 (Kd)	>10,000 (Kd)	[361, 362]
Tesofensine (NS 2330)	8 (EC_50_)–65 (EC_50_)	1.7 (EC_50_)–3.2 (EC_50_)	11 (EC_50_)	[364, 365]
Tianeptine	>10,000 (EC_50_)	>10,000 (EC_50_)	>10,000 (EC_50_)	[366]
Trazodone	7,400–37,419	5,000 (Kd), 34,000 (EC_50_)	160–690	[288, 330–332, 334, 336]
Trimipramine	3,780	2,450	149	[321]
UWA-101	1,270 (Kd), 3,600 (EC_50_)	>10,000	470 (Kd), 2,300 (EC_50_)	[367, 368]
UWA-121	307–592	>50,000	3,830–4,640	[368, 369]
UWA-122	>80,000	>50,000	120–340	[368, 369]
Vanoxerine (GBR-12,909)	1 (EC_50_)–51 (EC_50_)	79.2 (Kd), 2,600 (EC_50_)	73.2 (Kd), 170 (EC_50_)	[344, 347, 370]
Venlafaxine	3,070–9,300	210–2,480	7.5–145	[165, 288, 331, 334, 337]

D-: *dextro*; DAT: dopamine transporter; EC_50_: half-maximal effective concentration; Kd: dissociation constant; L-: *levo*; MDMA: 3,4-methylenedioxymethamphetamine; n/a: not available/not assessed; NET: noradrenaline transporter; PD: Parkinson's disease; R-: *rectus*; S-: *sinister*; SERT: serotonin transporter.

Affinity is provided as the Kd or EC_50_ (nM) and is the results of receptor binding and monoamine uptake assays. When not specified, the values provided refer to the Kd. For each compound, only the values reflecting the highest and lowest affinities encountered in the cited literature are mentioned and are provided as the extremes of a range. When data from literature are presented as both the Kd and EC_50_, we have used the symbol “,” instead of “–”, to indicate that the units of measure are different and that data presented should not be interpreted as being a range. The majority of the studies report the affinity as the arithmetic mean, but a few report it as the geometric mean.

**Table 2:** Selectivity profile of the monoamine reuptake inhibitors studied in idiopathic PD and animal models of PD.

Transporter	Compound
SERT	Citalopram, clomipramine, duloxetine, escitalopram, fenfluramine, fluoxetine, fluvoxamine, imipramine, paroxetine, R-MDMA, sertraline, trazodone, trimipramine, UWA-122, and venlafaxine

SERT = NET	Amitriptyline, milnacipran

NET	Amoxapine, amphetamine, atomoxetine, desipramine, L-amphetamine, maprotiline, mazindol, mianserin, mirtazapine, nisoxetine, nortriptyline, and reboxetine

DAT	Amineptine, modafinil, SEP-228,791, and vanoxerine

DAT = NET	Benztropine, brasofensine, bupropion, cocaine, D-amphetamine, methamphetamine, methylphenidate, nomifensine, and S,S-hydroxybupropion

DAT = SERT	UWA-101, UWA-121

DAT = SERT = NET	BTS 74,398, MDMA, nefazodone, S-MDMA, and tesofensine

SERT enhancer	Tianeptine

D-: *dextro*; DAT: dopamine transporter; L-: *levo*; MDMA: 3,4-methylenedioxymethamphetamine; NET: noradrenaline transporter; PD: Parkinson's disease; R-: *rectus*; S-: *sinister*; SERT: serotonin transporter.

In this table, all of the compounds were attributed a primary affinity based on the highest potency value displayed in Table 1. Compounds with more than 5-fold selectivity for a monoamine transporter were considered selective for this transporter (see Section 2).

**Table 3:** Summary of the effects of SERT inhibitors in idiopathic PD and animal models of PD.

	Animal models			Idiopathic PD			Other
	MPTP mouse	6-OHDA rat	MPTP NHP	Anxiety/depression	Parkinsonism	Dyskinesia	
Citalopram	↓ REM sleep duration	↓ AIMs severity after chronic treatment	n/a	Beneficial effect on depression and anxiety in the majority of studies	Inconsistent, but usually no/minor deterioration	n/a	Alters cerebral blood flow

Clomipramine	n/a	n/a	n/a	Possible beneficial effect on depression	No effect	n/a	n/a

Duloxetine	n/a	n/a	n/a	Possible beneficial effect on depression	Possible deterioration of tremor	n/a	Analgesic effect

Escitalopram	n/a	n/a	n/a	Possible beneficial effect on depression	No effect	n/a	Could trigger confusion and hallucinations

Fenfluramine	n/a	Induces bidirectional rotations; ↓ AIMs severity	n/a	n/a	No effect	n/a	↑ prolactin secretion

Fluoxetine	Possible protective effect against MPTP toxicity	↓ inhibition of neuronal firing in the LC; induces rotations ipsilateral to the lesion side; ↑ cellular proliferation in the SGZ; ↓ striatal dopamine levels after L-DOPA administration	n/a	Possible beneficial effect on depression	Inconsistent, but usually no/minor deterioration; improvement sometimes reported	↓ severity of apomorphine-induced dyskinesia	Improvement of Porsolt test in the VMAT_2_-deficient mouse; alters cerebral blood flow in human; possibly beneficial against orthostatic hypotension

Fluvoxamine	n/a	Does not induce rotations but ↑ rotations vanoxerine-induced rotations	n/a	Possible beneficial effect on depression	Inconsistent	n/a	n/a

Imipramine	n/a	n/a	n/a	Beneficial effect on depression in the majority of studies	Beneficial effect as monotherapy in the majority of studies	n/a	Interferes with gastrointestinal absorption of L-DOPA

Paroxetine	Possible protective effect against MPTP toxicity	n/a	n/a	Possible beneficial effect on depression and anxiety	No/minor deterioration	n/a	n/a

R-MDMA	n/a	No significant effect on rotational behaviour	Antidyskinetic effect in combination with L-DOPA; no effect on L-DOPA anti-Parkinsonian action	n/a	n/a	n/a	Counteracts haloperidol-induced catalepsy in the rat

Sertraline	n/a	n/a	Worsens Parkinsonism at high dose as monotherapy; improves parkinsonism at low dose as monotherapy; partially reverses the anti-Parkinsonian effect of vanoxerine	Possible beneficial effect on depression and anxiety	No effect	n/a	n/a

Trazodone	n/a	n/a	n/a	Possible beneficial effect on depression	No effect	Possible antidyskinetic effect	n/a

Trimipramine	n/a	n/a	n/a	No effect	n/a	n/a	n/a

UWA-122	n/a	n/a	No effect on L-DOPA anti-Parkinsonian action; possible antidyskinetic effect in combination with L-DOPA; no effect on L-DOPA-induced psychosis-like behaviours	n/a	n/a	n/a	n/a

Venlafaxine	n/a	n/a	n/a	Possible beneficial effect on depression	No effect	n/a	n/a

6-OHDA: 6-hydroxydopamine; AIMs: abnormal involuntary movements; L-: *levo*; LC: locus coeruleus; L-DOPA: L-3,4-dihydroxyphenylalanine; MDMA: 3,4-methylenedioxymethamphetamine; MPTP: 1-methyl-4-phenyl-1,2,3,6-tetrahydropyridine; n/a: not available/not assessed; NHP: nonhuman primate; PD: Parkinson's disease; R-: *rectus*; REM: rapid-eye movements; SGZ: subgranular zone VMAT_2_: vesicular monoaminergic transporter type 2.

**Table 4:** Summary of the effects of SERT = NET inhibitors in idiopathic PD and animal models of PD.

	Animal models			Idiopathic PD			Other
	MPTP mouse	6-OHDA rat	MPTP NHP	Anxiety/depression	Parkinsonism	Dyskinesia	
Amitriptyline	n/a	May protect against 6-OHDA toxicity; ↑ levels of BDNF and GDNF in the SN	n/a	Possible beneficial effect on depression; possibly more effective than fluoxetine	No effect/minor deterioration	n/a	Alleviates muscle-contraction headache

Milnacipran	n/a	n/a	n/a	Possible beneficial effect on depression	No effect	n/a	Possible beneficial effect on overactive bladder

6-OHDA: 6-hydroxydopamine; BDNF: brain-derived neurotrophic factor; GDNF: glial cell-line derived neurotrophic factor; MPTP: 1-methyl-4-phenyl-1,2,3,6-tetrahydropyridine; n/a: not available/not assessed; NHP: nonhuman primate; PD: Parkinson's disease; SN: substantia nigra.

**Table 5:** Summary of the effects of NET inhibitors in idiopathic PD and animal models of PD.

	Animal models			Idiopathic PD			Other
	MPTP mouse	6-OHDA rat	MPTP NHP	Anxiety/depression	Parkinsonism	Dyskinesia	
Amoxapine	n/a	n/a	n/a	n/a	Possible deterioration	Unclear	Possible ↓ of VHs

Amphetamine, methamphetamine, and propylhexedrine	↑ motor activity	Induces rotations ipsilateral to the lesioned side; alters striatal gene expression	n/a	Possible beneficial effect on depression	Inconsistent	Possible exacerbation	↓ of striatal dopamine levels in NHP and human; possible risk factor for PD; acute challenge ↑ dopamine release in PD patients

Atomoxetine	n/a	n/a	n/a	No effect on depression	Unclear	Possible exacerbation	Possibly enhances cognition; possibly reduces daytime sleepiness

Desipramine	↓ REM sleep duration	n/a	n/a	Possible beneficial effect on depression; onset of antidepressant effect, possibly quicker than citalopram	Inconsistent	Unclear	n/a

Maprotiline	n/a	n/a	n/a	n/a	Possible anti-Parkinsonian effect	n/a	Possibly enhances cognition and motivation

Mazindol	Protective effect against MPTP toxicity	n/a	n/a	n/a	Possible anti-Parkinsonian effect	n/a	n/a

Mianserin	n/a	n/a	n/a	n/a	Inconsistent	n/a	Possibly enhances attention and memory; possible antipsychotic effect

Mirtazapine	n/a	n/a	n/a	n/a	Possible benefit against tremor	Possible antidyskinetic effect	Could trigger psychotic symptoms and RBD

Nisoxetine	n/a	No effect on rotational behaviour	mild anti-Parkinsonian effect as monotherapy or in combination with sertraline; ↓ the anti-Parkinsonian effect of vanoxerine	n/a	n/a	n/a	n/a

Nortriptyline	n/a	n/a	n/a	Possible beneficial effect on depression	No effect	No effect	No effect

Reboxetine	n/a	↓ spontaneous firing of LC neurons; inhibitory effect on DRN neurons	n/a	Possible beneficial effect on depression and anxiety	No effect/minor deterioration	n/a	n/a

6-OHDA: 6-hydroxydopamine; DRN: dorsal raphe nucleus; LC: locus coeruleus; MPTP: 1-methyl-4-phenyl-1,2,3,6-tetrahydropyridine; n/a: not available/not assessed; NHP: nonhuman primate; PD: Parkinson's disease; RBD: REM-sleep behaviour disorder; REM: rapid-eye movements; VHs: visual hallucinations.

**Table 6:** Summary of the effects of DAT inhibitors in idiopathic PD and animal models of PD.

	Animal models			Idiopathic PD			Other
	MPTP mouse	6-OHDA rat	MPTP NHP	Anxiety/depression	Parkinsonism	Dyskinesia	
Amineptine	n/a	Protective effect against 6-OHDA toxicity	n/a	Possible beneficial effect on depression	n/a	n/a	n/a

Modafinil	Protective effect against MPTP toxicity	n/a	Protective effect against MPTP toxicity; anti-Parkinsonian effect as monotherapy	No effect on depression or anxiety	No effect	n/a	Possible beneficial effect on daytime sleepiness

SEP-228,791	n/a	n/a	Anti-Parkinsonian action as monotherapy, without eliciting dyskinesia; no effect on L-DOPA anti-Parkinsonian action or L-DOPA-induced dyskinesia	n/a	n/a	n/a	n/a

Vanoxerine	↓ REM and slow-wave sleep duration	Induces rotations ipsilateral to the lesion; these rotations are ↑ by fluvoxamine	Anti-Parkinsonian action as monotherapy, without eliciting dyskinesia	n/a	n/a	n/a	n/a

6-OHDA: 6-hydroxydopamine; L-: *levo*; L-DOPA: L-3,4-dihydroxyphenylalanine; MPTP: 1-methyl-4-phenyl-1,2,3,6-tetrahydropyridine; n/a: not available/not assessed; NHP: nonhuman primate; PD: Parkinson's disease; REM: rapid-eye movements.

**Table 7:** Summary of the effects of DAT = NET inhibitors in idiopathic PD and animal models of PD.

	Animal models			Idiopathic PD			Other
	MPTP mouse	6-OHDA rat	MPTP NHP	Anxiety/depression	Parkinsonism	Dyskinesia	
Brasofensine	n/a	n/a	Anti-Parkinsonian action as monotherapy, without eliciting dyskinesia; ↑ the anti-Parkinsonian action of low dose L-DOPA	n/a	Transient anti-Parkinsonian action as monotherapy; no effect on L-DOPA anti-Parkinsonian action	No effect	n/a

Bupropion	n/a	Protective effect against 6-OHDA toxicity; no effect on motor activity as monotherapy in bilateral 6-OHDA-lesioned rats	Inconsistent anti-Parkinsonian action as monotherapy	Possible beneficial effect on depression and anxiety	Inconsistent but possible beneficial effect	Unclear	n/a

Methylphenidate	n/a	Protective effect against 6-OHDA toxicity; induces rotations ipsilateral to the lesion	no effect in a delayed response task test	Possible beneficial effect on apathy; possible mood-enhancing effect	Inconsistent effects on gait; inconsistent effects on tremor; no effect on on-time duration	Unclear	No effect on striatal dopamine release; no effect on L-DOPA plasma levels; possible beneficial effect on daytime sleepiness; possible beneficial effect on attention; possible beneficial effect on pain

Nomifensine	n/a	n/a	Protective effect against MPTP toxicity; anti-Parkinsonian action as monotherapy	n/a	Possible anti-Parkinsonian benefit as monotherapy; inconsistent effect as add-on therapy	Possible deleterious effect	n/a

S,S-Hydroxybupropion	n/a	n/a	Anti-Parkinsonian action as monotherapy	n/a	n/a	n/a	n/a

6-OHDA: 6-hydroxydopamine; L-: *levo*; L-DOPA: L-3,4-dihydroxyphenylalanine; MPTP: 1-methyl-4-phenyl-1,2,3,6-tetrahydropyridine; n/a: not available/not assessed; NHP: nonhuman primate; PD: Parkinson's disease.

R,R-Hydroxybupropion does not exhibit significant affinity for any of the 3 monoamine transporters and, in the MPTP-lesioned marmoset, had no effect on motor activity or Parkinsonian disability.

**Table 8:** Summary of the effects of DAT = SERT inhibitors in idiopathic PD and animal models of PD.

	Animal models			Idiopathic PD			Other
	MPTP mouse	6-OHDA rat	MPTP NHP	Anxiety/depression	Parkinsonism	Dyskinesia	
UWA-101/121	n/a	n/a	↑ duration of on-time and “good quality on-time” in combination with L-DOPA; no effect on L-DOPA-induced dyskinesia; not tested as monotherapy	n/a	n/a	n/a	↑ L-DOPA-induced horizontal activity in the reserpine-treated rat

6-OHDA: 6-hydroxydopamine; L-: *levo*; L-DOPA: L-3,4-dihydroxyphenylalanine; MPTP: 1-methyl-4-phenyl-1,2,3,6-tetrahydropyridine; n/a: not available/not assessed; NHP: nonhuman primate; PD: Parkinson's disease.

**Table 9:** Summary of the effects of DAT = NET = SERT inhibitors in idiopathic PD and animal models of PD.

	Animal models			Idiopathic PD			Other
	MPTP mouse	6-OHDA rat	MPTP NHP	Anxiety/depression	Parkinsonism	Dyskinesia	
BTS 74,398	n/a	Induces rotations ipsilateral to the lesioned side; does not induce AIMs	Anti-Parkinsonian action as monotherapy; no effect on L-DOPA anti-Parkinsonian action; no effect on dyskinesia induced by a submaximal dose of L-DOPA	n/a	n/a	n/a	n/a

MDMA	n/a	Induces rotations ipsilateral to the lesioned side	Transient anti-Parkinsonian effect as monotherapy; antidyskinetic effect in combination with L-DOPA or pramipexole	n/a	↑ duration of on-time (anecdotal case-report)	Antidyskinetic effect (anecdotal case-report)	Counteracts haloperidol-induced catalepsy in the rat

Nefazodone	n/a	n/a	n/a	Possible beneficial effect on depression	Inconsistent, but possible beneficial effect	n/a	n/a

S-MDMA	n/a	Induces rotations ipsilateral to the lesioned side	↑ duration of L-DOPA anti-Parkinsonian action; ↑ dyskinesia severity	n/a	n/a	n/a	Counteracts haloperidol-induced catalepsy in the rat

Tesofensine	n/a	n/a	n/a	n/a	Nonsustained anti-Parkinsonian effect as monotherapy; possible ↓ of daily off-time in combination with L-DOPA	Possible ↑ of dyskinesia severity	Causes weight loss

6-OHDA: 6-hydroxydopamine; AIMs: abnormal involuntary movements; L-: *levo*; L-DOPA: L-3,4-dihydroxyphenylalanine; MDMA: 3,4-methylenedioxymethamphetamine; MPTP: 1-methyl-4-phenyl-1,2,3,6-tetrahydropyridine; n/a: not available/not assessed; NHP: nonhuman primate; PD: Parkinson's disease; S-: *sinister*.

**Table 10:** Summary of the effects of SERT enhancer in idiopathic PD and animal models of PD.

	Animal models			Idiopathic PD			Other
	MPTP mouse	6-OHDA rat	MPTP NHP	Anxiety/depression	Parkinsonism	Dyskinesia	
Tianeptine	n/a	n/a	n/a	Possible beneficial effect on depression	Possible beneficial effect	n/a	n/a

6-OHDA: 6-hydroxydopamine; MPTP: 1-methyl-4-phenyl-1,2,3,6-tetrahydropyridine; n/a: not available/not assessed; NHP: nonhuman primate; PD: Parkinson's disease.

## Abbreviations

*α*: Alpha-adrenergic receptors
*β*: Beta-adrenergic receptors
*σ*: Sigma receptors
*μ*M: Micromolar
[^3^H]: Labelled with tritium
5-HIAA: 5-Hydroxyindoleacetic acid
5-HT: Serotonin
6-OHDA: 6-Hydroxydopamine
[^11^C]: Labelled with radioactive carbon 11
AADC: Aromatic L-amino acid decarboxylase
AAN: American Academy of Neurology
AAV: Adeno-associated virus
ACTH: Adrenocorticotropic hormone
AIMs: Abnormal involuntary movements
AIMS: Abnormal Involuntary Movement Scale
am: Before noon
AMPA: *α*-Amino-3-hydroxy-5-methyl-4-isoxazolepropionic acid
BAI: Beck Anxiety Inventory
BBC: British Broadcasting Corporation
BDI: Beck Depression Inventory
BDNF: Brain-derived neurotrophic factor
bid: Twice a day
CGI: Clinical Global Impressions
Cl^−^: Chloride anion
Cmax: Maximal concentration
CREB: cAMP response element-binding
CRF: Corticotropin-releasing factor
D: Dopaminergic receptors
D-: Dextro-
D-amphetamine: Dextroamphetamine
DAT: Dopamine transporter
DOPAC: Dihydroxyphenylacetic acid
DRN: Dorsal raphe nucleus
EBM: Evidence based medicine
EC_50_: Half-maximal effective concentration
EDS: Excessive daytime sleepiness
ELLDOPA: Earlier versus Later Levodopa Therapy in Parkinson's Disease
ESS: Epworth Sleepiness Scale
Fos: FBJ murine osteosarcoma viral oncogene homolog
FSI: Fatigue Severity Index
FS1: Futility Study I
FS-TOO: Futility Study II
GABA: Gamma-aminobutyric acid
GDNF: Glial cell line-derived neurotrophic factor
GP: Globus pallidus
H: Histaminergic receptors
HADS: Hospital Anxiety and Depression Scale
HARS: Hamilton Anxiety Rating Scale
HDRS: Hamilton Depression Rating Scale
hs: * hora somni* (at bedtime)
HVA: Homovanillic acid
id: Once a day
i.m.: Intramuscular, intramuscularly
i.p.: Intraperitoneal, intraperitoneally
i.v.: Intravenous, intravenously
kb: Kilobase
kg: Kilogram
Kd: Dissociation constant
LC: Locus coeruleus
L-: Levo-
L-DOPA: L-3,4-Dihydroxyphenylalanine (levodopa)
L-amphetamine: Levoamphetamine
LRRK2: Leucine-rich repeat kinase 2
M: Muscarinic receptors
MADRS: Montgomery Asberg Depression Rating Scale
MAO: Monoamine oxidase
MDMA: 3,4-Methylenedioxymethamphetamine
MDS: Movement Disorders Society
mg: Milligram
MHPG, MOPEG: 3-Methoxy-4-hydroxyphenylglycol
MPP+: 1-Methyl-4-phenyl-pyridinium
MPTP: 1-Methyl-4-phenyl-1,2,3,6-tetrahydropyridine
MTP: 1-Methyl-1,2,3,6-tetrahydropyridine
Na^+^: Sodium cation
NAA: *N*-Acetyl-aspartate
NET: Norepinephrine transporter
nM: Nanomolar
NMDA: *N*-Methyl-D-aspartate
Nurr1: Nuclear receptor related 1 protein
PD: Parkinson's disease
PDZ: Postsynaptic density protein (PSD_95_), drosophila disc large tumour suppressor (DlgA), and zonula occludens-1 protein (zo-1)
PET: Positron emission tomography
PICK_1_: PRCKA-binding protein
PKC: Protein kinase C
p.o.: * per os* (orally)
PP_2A_: Protein phosphatase 2A
PPE: Preproenkephalin
PRECEPT: Parkinson Research Examination of CEP1348 Trial
QE2: Effects of CoEnzyme Q10 in Early Parkinson's Disease
qid: Four times a day
R-: Rectus
RBD: REM-sleep behaviour disorder
rCBF: Regional cerebral blood flow
REM: Rapid-eye-movement
S-: Sinister
SAD-PD: Study of Antidepressants in Parkinson's Disease
s.c.: Subcutaneous, subcutaneously
SERT: Serotonin transporter
SGZ: Subgranular zone
SN: Substantia nigra
SPECT: Single-photon emission computed tomography
SSRI: Selective serotonin reuptake inhibitor
STN: Subthalamic nucleus
TCA: Tricyclic antidepressant
TEMPO: TVP-1012 in Early Monotherapy for Parkinson's Disease Outpatients
tid: Three times a day
UPDRS: Unified Parkinson's Disease Rating Scale
UWA: University of Western Australia
VAS: Visual Analogue Scale
VMAT: Vesicular monoaminergic transporter.
